# Melatonin Ameliorates Depressive‐Like Behaviors in Ovariectomized Mice by Improving Tryptophan Metabolism via Inhibition of Gut Microbe *Alistipes Inops*

**DOI:** 10.1002/advs.202309473

**Published:** 2024-07-08

**Authors:** Kai‐Yu Zheng, Bo Gao, Hua‐Jie Wang, Jin‐Gang He, Hong‐Sheng Chen, Zhuang‐Li Hu, Li‐Hong Long, Jian‐Guo Chen, Fang Wang

**Affiliations:** ^1^ State Key Laboratory for Diagnosis and Treatment of Severe Zoonotic Infectious Diseases Department of Pharmacology School of Basic Medicine Tongji Medical College Huazhong University of Science and Technology Wuhan China; ^2^ The Research Center for Depression Tongji Medical College Huazhong University of Science Wuhan 430030 China; ^3^ The Key Laboratory for Drug Target Researches and Pharmacodynamic Evaluation of Hubei Province Wuhan 430030 China; ^4^ Hubei Shizhen Laboratory Wuhan 430030 China; ^5^ Laboratory of Neuropsychiatric Diseases The Institute of Brain Research Huazhong University of Science and Technology Wuhan 430030 China

**Keywords:** gut microbiota, melatonin, menopausal depression, tryptophans

## Abstract

Melatonin (*N*‐acetyl‐5‐methoxytryptamine) is reported to improve mood disorders in perimenopausal women and gut microbiome composition is altered during menopausal period. The possible role of microbiome in the treatment effect of melatonin on menopausal depression remains unknown. Here, it is shown that melatonin treatment reverses the gut microbiota dysbiosis and depressive‐like behaviors in ovariectomy (OVX) operated mice. This effect of melatonin is prevented by antibiotic cocktails (ABX) treatment. Transferring microbiota harvested from adolescent female mice to OVX‐operated mice is sufficient to ameliorate depressive‐like behaviors. Conversely, microbiota transplantation from OVX‐operated mice or melatonin‐treated OVX‐operated mice to naïve recipient mice exhibits similar phenotypes to donors. The colonization of *Alistipes Inops*, which is abundant in OVX‐operated mice, confers the recipient with depressive‐like behaviors. Further investigation indicates that the expansion of *Alistipes Inops* induced by OVX leads to the degradation of intestinal tryptophan, which destroys systemic tryptophan availability. Melatonin supplementation restores systemic tryptophan metabolic disorders by suppressing the growth of *Alistipes Inops*, which ameliorates depressive‐like behaviors. These results highlight the previously unrecognized role of *Alistipes Inops* in the modulation of OVX‐induced behavioral disorders and suggest that the application of melatonin to inhibit *Alistipes Inops* may serve as a potential strategy for preventing menopausal depressive symptoms.

## Graphical abstract

The study identifies the previously unrecognized role of *Alistipes Inops*‐mediated tryptophan‐5‐HT metabolism disorders in ovariectomized mice. Melatonin prevents the disturbance of tryptophan‐5‐HT metabolism by inhibition of *Alistipes Inops* and thus ameliorates depressive‐like behaviors in ovariectomized mice. Melatonin or other drugs that can induce inhibition of *Alistipes Inops* can provide promising new therapeutic possibilities for menopausal depression.

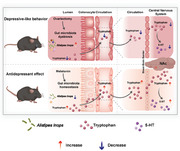

## 1 Introduction

Major depressive disorder (MDD) has become one of the leading causes of disability worldwide, affecting over 10% of the general population.^[^ ^1^ ^]^ Epidemiological studies have shown that the lifetime prevalence of MDD in women is almost twice that in men.^[^ ^2^ ^]^ Women in the menopausal transition are at increased risk of MDD,^[^ ^3^, ^4^ ^]^ possibly due to the significant decline of gonadal hormones, such as estrogens and progestins.^[^ ^5^ ^]^ Accumulating evidence has implicated gonadal hormones in alleviating depressive symptoms in mouse models of menopause.^[^ ^6^, ^7^ ^]^ However, gonadal hormone replacement therapy for menopausal depressive symptoms remains controversial.

The association of chronologic aging with reproductive aging is more prominent in midlife women than in men. Specifically, perturbations in gonadal hormones lead to sleep disturbances and disorders during the menopause period.^[^ ^8^ ^]^ Melatonin (N‐acetyl‐5‐methoxytryptamine), a hormone that assists in the governing of sleep and circadian rhythms, is synthesized in the pineal gland and gastrointestinal tract.^[^ ^9^ ^]^ Aging is related to the decreased secretion of melatonin and disturbance in the circadian system, leading to an alteration in sleep architecture. In addition, similar to gonadal hormone therapy, melatonin treatment can improve sleep quality in menopausal women and facilitate metabolic health.^[^ ^10^, ^11^ ^]^ Melatonin has comparable efficacy to gonadal hormone at improving menopause‐related symptoms. It has been demonstrated that melatonin supplementation relieves psychosomatic symptoms in postmenopausal women.^[^ ^12^ ^]^ Another clinical trial shows that perimenopausal women who receive melatonin administration improve mood and relieve morning depression.^[^ ^13^ ^]^ Agomelatine, a synthetic analog of melatonin, exerts an antidepressant effect mainly by mimicking the action of melatonin in the brain.^[^ ^14^ ^]^ It has been reported that exogenous administration of melatonin promotes neurogenesis and relieves chronic corticosterone‐induced depressive behaviors.^[^ ^15^ ^]^ However, the role of melatonin in menopausal depression remains obscure.

Previous studies demonstrate the antioxidant effects of melatonin and its metabolites, including the capacity to reduce oxidative stress and inhibit inflammation and apoptosis.^[^ ^16^ ^]^ Recently, melatonin has been considered to regulate brain functions by alteration of commensal microorganisms in the gut. In addition, melatonin has been shown to reshape gut microbiota and improve several neuropsychiatric disorders, including cognitive impairment, autism, and weanling stress.^[^ ^17^, ^18^, ^19^ ^]^ Emerging evidence shows that gut microbiota and their metabolites impact the pathophysiology of depression. For example, germ‐free mice transplanted with fecal microbiota from depressed patients show depressive‐like phenotypes.^[^ ^20^ ^]^ Furthermore, a recent study shows that mice receiving microbiota from depressed patients exhibit emotionally impaired phenotypes and alteration of microbial metabolites, such as an increase in proline metabolism.^[^ ^21^ ^]^ A clinical trial reveals the differences in gut microbiota composition between postmenopausal women and premenopausal women.^[^ ^22^ ^]^ However, it is poorly understood whether and how the microbiome plays a role in menopausal depression.

In this study, by using the mouse model of menopausal depression, we revealed that exogenous administration of melatonin reshaped the composition and structure of gut microbiota, leading to the alleviation of depressive‐like behaviors induced by ovariectomy (OVX). We found that OVX‐induced perturbations of gut microbiota, and melatonin treatment produced antidepressant effects through normalizing tryptophan metabolism. Our results highlighted a previously unrecognized role of *Alistipes Inops* in the modulation of OVX‐induced behavioral disorders and identified the application of melatonin in inhibiting *Alistipes Inops* as a potential strategy for preventing menopausal depressive symptoms.

## 2 Results

### 2.1 Exogenous Supplementation of Melatonin Ameliorates OVX‐Induced Depressive‐Like Behaviors in Mice

**Figure 1:**
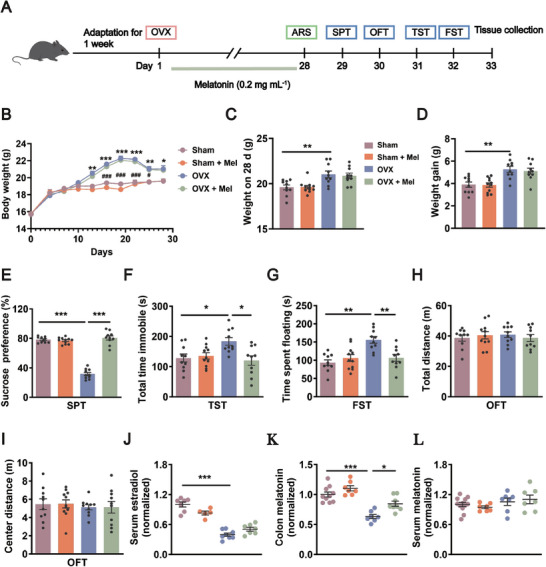
Exogenous supplementation of melatonin ameliorates OVX‐induced depressive‐like behaviors in mice. A) Schematic illustration of the experimental design. B) Body weight was monitored. *n* = 10–11 per group. C) Body weight on day 28. *n* = 10–11 per group. D) Weight gain from OVX operation until day 28. *n* = 10–11 per group. E) The sucrose‐in‐water ratio during SPT of Sham mice, Sham + Mel mice, OVX mice, and OVX + Mel mice. *n* = 10–11 per group. F) Total time immobile in the TST. *n* = 10–11 per group. G) The floating time in the FST. *n* = 10–11 per group. H) Total distance during OFT. *n* = 10–11 per group. I) Distance spent in the center during OFT. *n* = 10–11 per group. J) Serum level of estradiol was analyzed by ELISA. *n* = 5–8 per group. K) ELISA analysis of colonic melatonin protein normalized to controls. *n* = 7–10 per group. L) Melatonin level in serum was measured by ELISA. *n* = 7–12 per group. Data are the mean ± SEM and analyzed by three‐way ANOVA (B) or two‐way ANOVA (C–L) with Bonferroni's post‐hoc test, *p < 0.05, **p < 0.01, ***p < 0.001 Sham versus OVX; ^#^ *p <* 0.05, ^###^ *p <* 0.001 Sham versus OVX + Mel; Mel: melatonin, ARS: acute restraint stress, SPT: sucrose preference test, OFT: open field test, TST: tail suspension test, FST: forced swim test, Sham: sham‐operated mice, Sham + Mel: melatonin treated sham‐operated mice, OVX: ovariectomy‐operated mice, OVX + Mel: melatonin treated ovariectomy‐operated mice.

To investigate the effect of melatonin on OVX‐induced depressive‐like behaviors, C57BL/6J female mice received OVX surgery followed by acute restraint stress 28 days after the surgery. During this period, melatonin was continuously administered in the drinking water at a dose of 0.05, 0.1, 0.2, and 0.4 mg mL^−1^ (**Figure** **1**A; Figure S1A, Supporting Information). It was shown that only 0.4 mg mL^−1^ melatonin impaired the locomotor activity of OVX‐operated mice (Figure S1B, Supporting Information). Compared with 0.05 mg mL^−1^ or 0.1 mg mL^−1^ melatonin, 0.2 mg mL^−1^ melatonin increased sucrose preference and reduced immobility time in the tail suspension test of OVX‐operated mice (Figure S1C,D, Supporting Information). Additionally, melatonin at concentrations of 0.05, 0.1, and 0.2 mg mL^−1^ exerted little influence on center distance in OVX‐operated mice as measured by the open field test (Figure S1E, Supporting Information). Therefore, 0.2 mg mL^−1^ melatonin was employed in the following experiments.

It was found that the body weight of OVX‐operated mice was increased on day 13, which was much higher than that in the sham group on day 28 (Figure 1B,C). In addition, the OVX‐induced weight gain was much higher than that of control mice (Figure 1D). However, melatonin at 0.2 mg mL^−1^ did not affect the changes in body weight induced by OVX (Figure 1B–D).

The behavioral results showed that OVX‐operated mice displayed decreased sucrose preference in the sucrose preference test, and increased immobility time in the tail suspension test and forced swim test compared with that in the sham group, demonstrating that OVX induces depressive‐like behaviors in female mice. However, the depressive‐like behaviors induced by OVX were reversed by continuous administration of 0.2 mg mL^−1^ melatonin in the drinking for 28 days (Figure 1E–G), without effect on locomotor activity and center distance as determined by the open field test (Figure 1H,I). The above results suggest that daily treatment with 0.2 mg mL^−1^ melatonin prevents depressive‐like phenotypes induced by OVX without affecting body weight.

Consistent with previous studies that OVX reduces the level of serum estradiol,^[^ ^23^ ^]^ we observed that the level of estradiol was decreased in the serum of OVX‐operated mice compared with that in the sham group. However, 0.2 mg mL^−1^ melatonin did not reverse the reduction of serum estradiol‐induced by OVX (Figure 1J), indicating that exogenous melatonin‐produced antidepressant effect is independent of the level of serum estrogen. Considering that the nocturnal melatonin concentration was decreased in the serum of postmenopausal women,^[^ ^24^ ^]^ and melatonin levels in the gastrointestinal tract are 400 times higher than that in the pineal,^[^ ^9^ ^]^ we asked whether OVX affects the level of melatonin in the colon. It was found that OVX decreased the level of melatonin in the colon compared with that in the sham group, which was reversed by daily treatment with 0.2 mg mL^−1^ melatonin (Figure 1K). However, there was no difference in the level of serum melatonin among the four groups (Figure 1L), suggesting that exogenous administration of melatonin affects the melatonin level in the colon, but not in the serum, which may be relevant to the antidepressant effect of melatonin.

Previous studies demonstrate that chronic restraint stress (CRS) disrupts the colonic mucosal barrier and induces systemic inflammation,^[^ ^25^ ^]^ and OVX leads to a decline in the expression of tight junction proteins in the colon and an increase in lipopolysaccharide level in the serum.^[^ ^26^ ^]^ Therefore, we wondered whether the deficiency of intestinal mucosal barrier was involved in the OVX‐induced depressive‐like behaviors. Contrary to previous studies, the results showed no changes in epithelial crypt architecture of colon among the three groups (Figure S2A, Supporting Information). In addition, the mRNA levels of tight junction‐related proteins, including claudin‐1, occludin, and ZO‐1 were not affected by OVX and acute restraint stress (Figure S2B–D, Supporting Information). Similarly, the mRNA levels of the proinflammatory cytokines, including IL‐1β, IL‐6, and TNF‐α in the colon were not altered (Figure S2E–G, Supporting Information), indicating that OVX‐induced depressive‐like behaviors are not related to the alteration in colon mucosal barrier.

### 2.2 Melatonin Produces Antidepressant Effects in a Microbiota‐Dependent Manner

**Figure 2:**
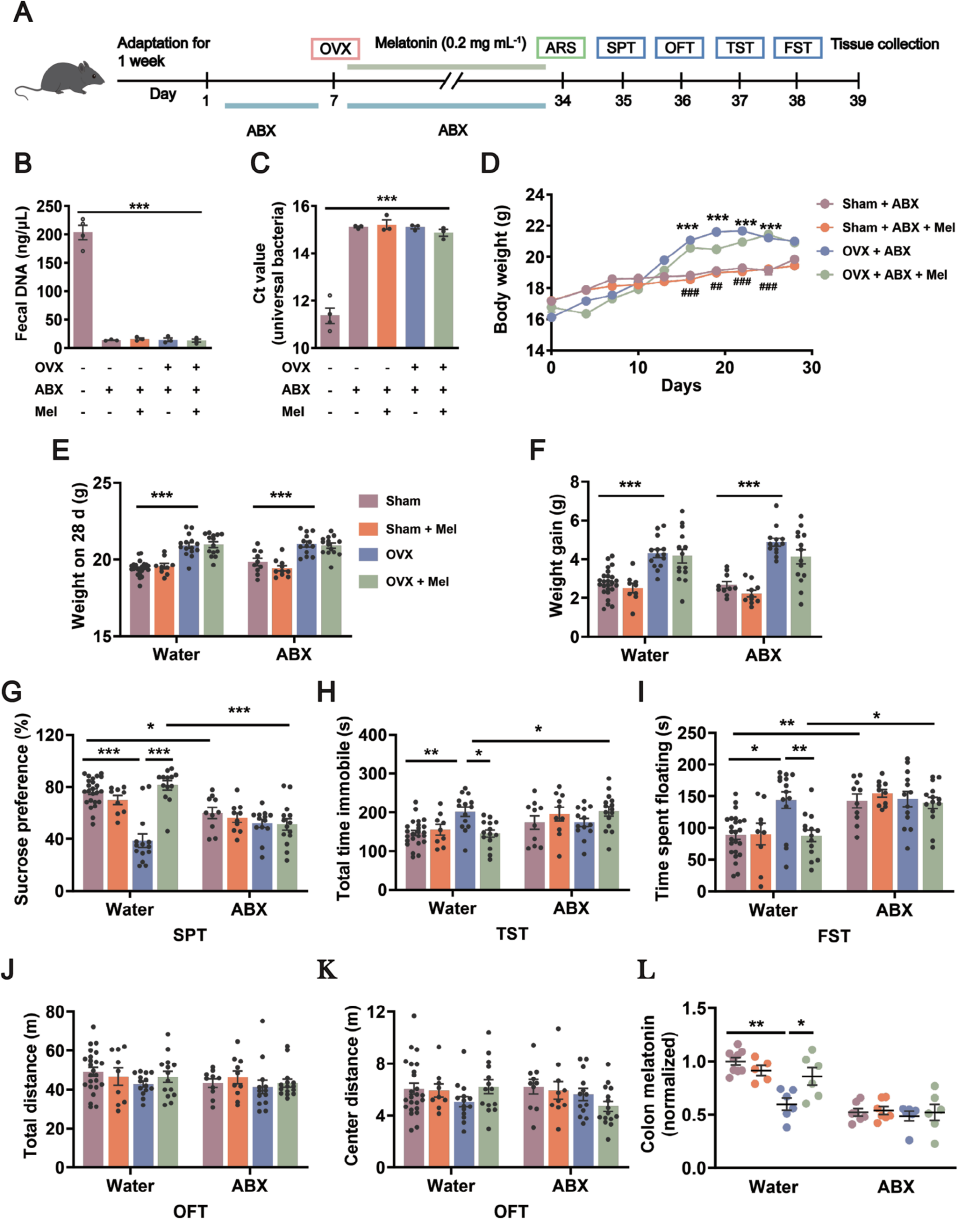
Melatonin produces antidepressant effects in a microbiota‐dependent manner. Sham mice, Sham + Mel mice, OVX mice, and OVX + Mel mice were treated with ABX or without ABX (Water). A) Schematic illustration of experimental design for depletion of the gut microbiota. B, C) Fecal DNA was isolated from ABX‐treated or no‐treated mice. B) DNA concentration was measured. *n* = 3–4 per group. C) 30 ng of DNA was used for quantification of the universal bacterial 16S rDNA gene by qRT‐PCR. *n* = 3–4 per group. Ct values of qRT‐PCR reflect relative bacterial burden in different treatment groups. D) Body weight was monitored after ABX treatment. *n* = 10–14 per group. E) Body weight on day 28 after OVX operation with water treatment (left) or ABX treatment (right). *n* = 9–25 per group. F) Weight gain during water treatment(left) or ABX treatment (right). *n* = 9–25 per group. G) The sucrose‐in‐water ratio during SPT after water treatment (left) or ABX treatment (right). *n* = 9–24 per group. H) Total time immobile in the TST after water treatment (left) or ABX treatment (right). *n* = 9–24 per group. I) The floating time in the FST after water treatment (left) or ABX treatment (right). *n* = 9–24 per group. J) Total distance during OFT after water treatment (left) or ABX treatment (right). *n* = 9–24 per group. K) Distance spent in the center zone during OFT after water treatment (left) or ABX treatment (right). *n* = 9–24 per group. L) ELISA quantification of melatonin after water treatment (left) or ABX treatment (right). *n* = 5–9 per group. All of the data are the mean ± SEM and analyzed by one‐way ANOVA (B–C) or three‐way ANOVA (D–L) with Bonferroni's post‐hoc test, *p < 0.05, **p < 0.01, ***p < 0.001 Sham + ABX versus OVX + ABX; ^##^ *p <* 0.01, ^###^ *p <* 0.001 Sham + ABX versus OVX + ABX + Mel.

Previous studies have shown that disruption of the gut microbiome leads to behavioral abnormalities in mice, such as social deficits,^[^ ^27^, ^28^ ^]^ and the change in the gut microbiome is involved in the antioxidant and anti‐inflammatory effects of melatonin.^[^ ^29^, ^30^ ^]^ Given exogenous administration of melatonin affects the melatonin level in the colon, but not in the serum in the OVX‐treated mice, we proposed the hypothesis that the antidepressant effects of melatonin were dependent on the gut microbiota. To determine the requirement of microbiota in OVX‐induced depressive‐like behaviors and the role of microbiota in the antidepressant effects of melatonin, female mice were given antibiotic cocktails (ABX) ad libitum via drinking water, and then subjected to OVX surgery and fed with melatonin (**Figure** **2**A). Our results showed that ABX treatment reduced the concentration of fecal bacterial DNA and increased the cycle threshold value of universal bacterial 16S rDNA gene compared with that in mice without ABX treatment, suggesting ABX treatment depletes microbial load in the gut (Figure 2B,C). After ABX treatment, although the body weight of OVX‐operated mice increased compared with that in the sham group, melatonin treatment failed to affect the body weight of OVX‐operated mice (Figure 2D,E). Furthermore, the OVX‐induced weight gain was much higher than that of sham mice with or without ABX. Nevertheless, weight gain in OVX‐operated mice and melatonin‐treated OVX‐operated mice were comparable in the context of ABX or water (Figure 2F). The above results suggest that ABX or melatonin supplementation does not impact body weight and weight gain.

As shown in Figure 2G, ABX treatment prevented OVX‐induced decrease in sucrose preference and blocked melatonin‐induced reverse in the deficit of sucrose preference caused by OVX. Compared with water‐treated mice, ABX treatment decreased sucrose preference and increased immobility time in the forced swim test of sham mice. However, no statistical differences were observed in immobility time in tail suspension test between sham mice with or without ABX treatment. Additionally, ABX treatment abolished the differences in immobility time in both tail suspension tests and forced swim tests between OVX groups treated with or without melatonin (Figure 2H,I). Furthermore, compared with water‐treated mice, ABX treatment prevented the melatonin‐induced decrease in immobility time in both tail suspension test and forced swim test of OVX‐operated mice, indicating that gut microbiota contributes to melatonin‐mediated improvement of despair behaviors induced by OVX. Meanwhile, ABX administration did not alter the locomotor activity and the center distance among all the groups in the open field test (Figure 2J,K).

Considering that in our study, the level of melatonin in the colon was reduced in OVX‐operated mice, and previous study demonstrates that the gut microbiota produces melatonin in vitro,^[^ ^31^ ^]^ we asked whether the gut microbiota was involved in the changes in the level of colonic melatonin induced by OVX. As shown in Figure 2L, there were no significant differences in the levels of colon melatonin in all the groups after treatment with ABX, indicating that both the antidepressant effects of exogenous melatonin and the level of colonic melatonin are dependent on the gut microbiota.

### 2.3 Fecal Microbiota Transplantation from Either Adolescent Female Mice or Melatonin‐Treated OVX Mice are Sufficient to Relieve OVX‐Induced Depressive‐Like Behaviors

**Figure 3:**
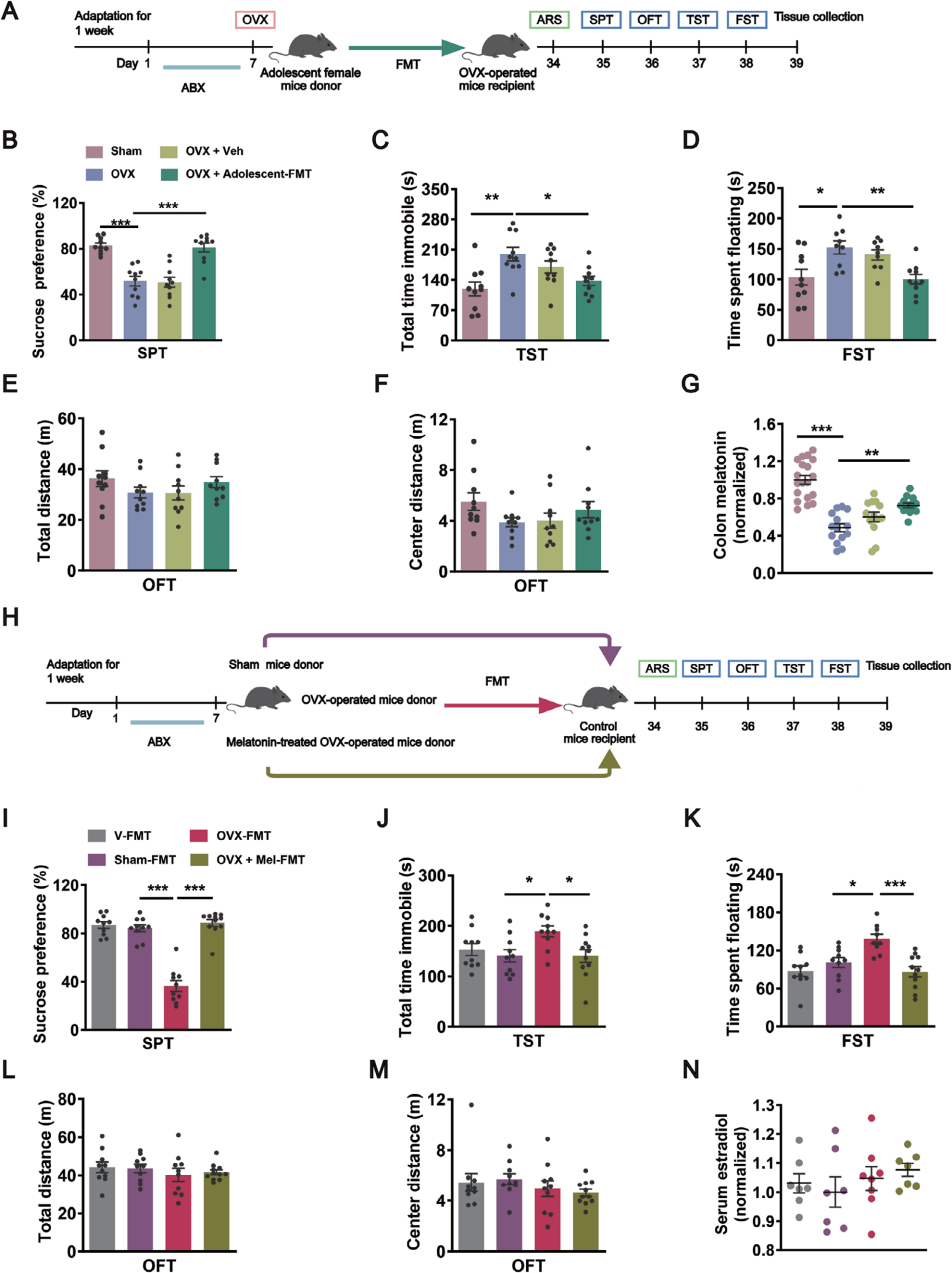
FMT from either adolescent female mice or melatonin‐treated OVX mice is sufficient to relieve OVX‐induced depressive‐like behaviors. A) Schematic illustration of experimental design for assessing the effect of microbiota from adolescent female mice to OVX‐operated mice. B) The sucrose‐in‐water ratio during SPT of Sham mice, OVX mice, OVX + Vehicle mice, and OVX + Adolescent‐FMT mice. *n* = 10 per group. C) Total time immobile in the TST. *n* = 10 per group. D) The floating time in the FST. *n* = 9–10 per group. E) Total distance during OFT. *n* = 10 per group. F) Distance spent in the center zone during OFT. *n* = 10 per group. G) ELISA quantification of melatonin protein level in the colon normalized to controls. *n* = 13–19 per group. H) Schematic of the experimental design for assessing the effect of microbiota from OVX‐operated mice and melatonin‐treated OVX‐operated mice to recipient mice that were pretreated with ABX. I) The sucrose‐in‐water ratio during SPT of V‐FMT mice, Sham‐FMT mice, OVX‐FMT mice, and OVX + Mel‐FMT mice. *n* = 10–11 per group. J) Total time immobile in the TST. *n* = 10–11 per group. K) The floating time in the FST. *n* = 9–11 per group. L) Total distance during OFT. *n* = 10–11 per group. M) Distance spent in the center zone during OFT. *n* = 10–11 per group. N) Serum level of estradiol was analyzed by ELISA. *n* = 7–8 per group. All of the data are the mean ± SEM and analyzed by one‐way ANOVA (B–G and I–N) with Bonferroni's post‐hoc test, *p < 0.05, **p < 0.01, ***p < 0.001. Sham: sham‐operated mice, OVX: ovariectomy‐operated mice, OVX + Veh: ovariectomy‐operated mice treated with sterile saline, OVX + Adolescent‐FMT mice: ovariectomy‐operated mice received microbiota from adolescent female donor mice, V‐FMT: naïve female mice received sterile saline treatment, Sham‐FMT: naïve female mice received microbiota from sham‐operated donor mice, OVX‐FMT: naïve female mice received microbiota from ovariectomy‐operated donor mice, OVX + Mel‐FMT: naïve female mice received microbiota from melatonin‐treated ovariectomy‐operated donor mice.

Considering that transfer microbiota of depressed premenopausal women into mice has been reported to induce depression‐like behaviors,^[^ ^32^ ^]^ we assessed the effects of fecal microbiota transplantation (FMT) from adolescent female mice into OVX‐operated mice on depressive‐like behaviors (**Figure** **3**A). Four weeks after FMT, recipient mice that received microbiota from adolescent mice exhibited increased sucrose preference in the sucrose preference test, and shorter immobility time in both the tail suspension test and forced swim test compared with that in OVX‐operated mice (Figure 3B–D), with no effects on locomotor activity and the center distance in the open field test (Figure 3E,F), suggesting that the gut microbiota from adolescent female mice alleviates depressive‐like behaviors induced by OVX. Furthermore, we observed an increase in the level of colonic melatonin after FMT from adolescent female mice in OVX‐operated mice compared with that in OVX‐operated mice (Figure 3G), which was similar to the above results that exogenous melatonin supplementation increased the colonic melatonin levels in OVX‐operated mice. These results suggest that the gut microbiota in adolescent female mice elevates the level of intestinal melatonin.

To further determine the role of gut microbiota in the antidepressant‐like effects of melatonin, the recipient female mice were pretreated with ABX, followed by FMT harvested from sham (Sham‐FMT), OVX‐operated (OVX‐FMT) and melatonin + OVX‐treated mice (OVX + Mel‐FMT) (Figure 3H). It was shown that OVX‐FMT mice produced depressive‐like phenotypes, which displayed lower sucrose preference and increased immobility time in both tail suspension test and forced swim test. However, OVX‐FMT‐induced depressive‐like behaviors were ameliorated by the FMT harvested from melatonin + OVX‐treated mice (Figure 3I–K). There were no changes in locomotor activity and center distance among the groups (Figure 3L–M).

It has been reported that gut microbiota from premenopausal women with depression reduce serum estradiol in mice and induce depression‐like behaviors.^[^ ^32^ ^]^ Given that the degradation of estradiol was mediated by gut microbes, the level of serum estradiol was measured. As shown in Figure 3N, there were no differences in the level of serum estradiol among these groups, indicating gut microbiota exerted no effect on serum estradiol in OVX or melatonin‐treated OVX mice. These results indicate that FMT from adolescent female mice reverses depressive‐like behaviors induced by OVX and the necessity of gut microbiota in the antidepressant effects of melatonin on OVX model of depression.

### 2.4 Exogenous Melatonin Supplementation Reshapes the Gut Microbiota in Ovariectomized Mice

**Figure 4:**
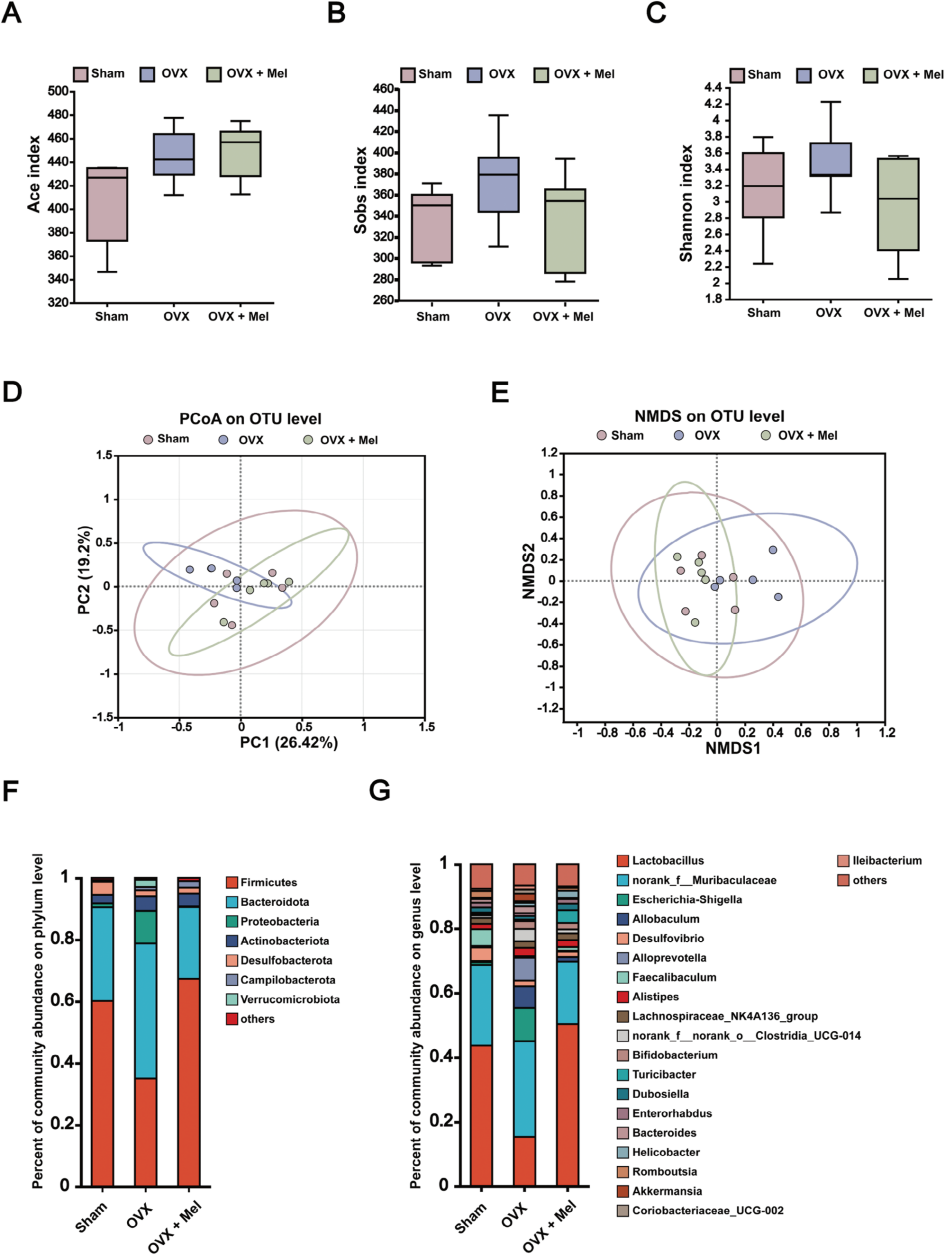
Exogenous melatonin supplementation reshapes the gut microbiota in ovariectomized mice. A–H) WT female mice were submitted to OVX and fed with 0.2 mg mL^−1^ melatonin in drinking water for 28 days. Stool samples were collected, and genomic DNA was isolated and sequenced for 16S rDNA gene at the V3‐V4 region. αDiversity was calculated by Ace index A), Sobs index B), and Shannon index C). *n* = 5 per group. D,E) β‐Diversity analysis of gut microbiota of different groups based on the OTU using the PCoA D) and NMDS E). *n* = 5 per group. F,G) Percent of community abundance on the phylum level F) and the genus level G). *n* = 5 per group.

Previous studies have reported that melatonin supplementation reshapes the gut microbiota in rodent animal models, such as weanling stress, high‐fat diet, and autism.^[^ ^17^, ^18^, ^19^ ^]^ However, less is known about the ability of exogenous melatonin to modulate gut microbiota under OVX conditions. Therefore, the fecal samples were collected from sham, OVX‐operated, and melatonin + OVX‐treated mice. 16S rDNA sequencing was used to measure the changes in the abundance of bacterial DNA in fecal samples. By analyzing the alpha‐diversity via Ace, Sobs, and Shannon's index, we did not observe any significant differences in bacterial diversity or richness among the groups (**Figure** **4**A–C), indicating that OVX or melatonin‐treated OVX exerted no effect on the richness and uniformity of intestinal flora in mice. As reflected by distinct clustering patterns on principal coordinate analysis (PCoA) plots and non‐metric multidimensional scaling (NMDS), there was a separation in the structure and the community composition between the sham and OVX groups. In addition, melatonin + OVX‐treated mice showed a similar microbial population to sham mice (Figure 4D,E). These results suggest that melatonin reverses the alteration of gut microbiota composition induced by OVX.

Next, we characterized the relative abundance of different bacterial taxa in all groups. On the phylum level, the composition of the gut microbiota was altered in ovariectomized mice compared with that in the sham group and melatonin‐treated mice, such as an expansion of *Bacteroidota* and *Proteobacteria* and a contraction of *Firmicutes* (Figure 4F). On the genus level, compared with that in the other two groups, ovariectomized mice showed a higher abundance of *norank_f_Muribaculaceae*, *Escherichia‐Shigella*, *Allobaculum*, *Alloprevptella*, *Alistipes*, and *Akkermansia*, but lower abundance of *Lactobacillus* (Figure 4G). Together, these findings imply that melatonin reshapes the gut microbiota in ovariectomized mice, which is characterized by a variation in dominant species, as well as an alteration in species abundance.

### 2.5 Melatonin Selectively Suppresses the Proliferation of *Alistipes Inops* in Ovariectomized Mice

**Figure 5:**
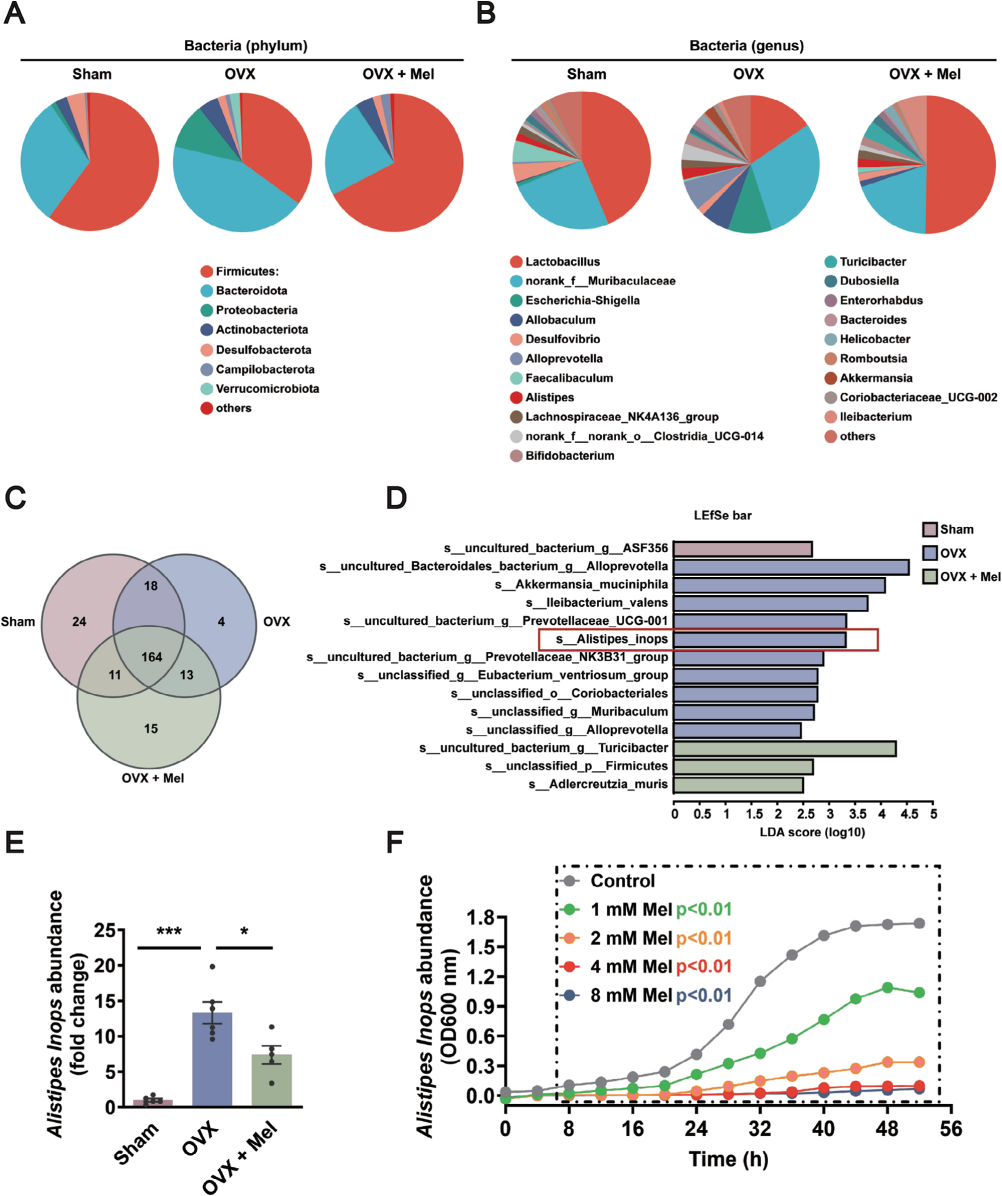
Melatonin selectively suppresses the proliferation of *Alistipes Inops* in ovariectomized mice. A, B) Relative abundance of fecal bacteria of Sham, OVX, and OVX + Mel mice on the phylum level A) and the genus level B). *n* = 5 per group. C) Venn diagrams illustrating the number of shared and exclusive bacteria at the species level under different conditions. *n* = 5 per group. D) Differences in microbial taxa at the species level between different groups were calculated by LEfSe. Kruskal‐Wallis test was used with a statistical significance cut‐off of *p* < 0.01 and LDA score > 2.00. E) Relative abundance of *Alistipes Inops* in the feces of Sham, OVX, and OVX + Mel mice. *n* = 5–6 per group. F) The growth curves of *Alistipes Inops* under melatonin treatment with various dosages in vitro (1, 2, 4, and 8 mm) OD 600 nm. *n* = 3 per group. Data are the mean ± SEM and calculated by one‐way ANOVA E) or two‐way ANOVA F) with Bonferroni's post‐hoc test, *p < 0.05, ***p < 0.001.

The bacteria community analysis pie plot was used to further confirm the effects of melatonin on alteration in gut bacterial abundance of ovariectomized mice. On the phylum level, melatonin treatment reduced the *Bacteroidota* and *Proteobacteria*, but increased *Firmicutes* in ovariectomized mice (**Figure** **5**A). In addition, on the genus level, relative abundances of *Lactobacillus* were increased, whereas *Allobaculum*, *Alloprevptella*, *Alistipes*, and *Akkermansia* were reduced in OVX‐operated mice after treatment with melatonin (Figure 5B). Venn diagrams were used to illustrate the number of shared and exclusive bacteria on the species level among the three groups. The results showed that 164 bacteria were co‐occurrence in all groups. Furthermore, 24 and 4 bacteria were only presented in sham mice and OVX‐operated mice, while the ovariectomized mice treated with melatonin showed the unique presence of 15 bacteria (Figure 5C), highlighting that melatonin treatment reverses OVX‐induced intestinal dysbacteriosis.

To identify the specific bacteria associated with the melatonin treatment, the linear discriminant analysis effect size (LEfSe) algorithm was used to perform linear discriminant analysis (LDA) and identify operational microbial taxa that were differentially abundant with melatonin treatment. As shown in Figure 5D, on the species level, sham mice exhibited a higher abundance of *uncultured bacterium ASF356*, but *Akkermansia muciniphila*, *Ileibacterium valens*, and *Alistipes Inops* were among the most abundant bacteria in OVX‐operated mice, and melatonin administration increased the abundance of *Adlercreutzia muris*. Earlier research found that patients with major depressive disorder show increased levels of *Alistipes* in their feces.^[^ ^33^, ^34^, ^35^ ^]^ Animal studies have also demonstrated consistent findings that the abundance of *Alistipes* is increased in mouse models of depression.^[^ ^36^, ^37^ ^]^ Those results prompted us to examine whether *Alistipes Inops* was involved in the antidepressant effects of melatonin. As shown in Figure 5E, compared with sham mice, OVX‐operated mice showed a higher abundance of *Alistipes Inops*, which was prevented by melatonin administration, indicating that melatonin has a negative effect on *Alistipes Inops* in OVX‐operated mice. To further assess whether melatonin inhibited the growth of *Alistipes Inops*, the growth curve with different concentrations of melatonin at 1, 2, 4, and 8 mm was measured in vitro. Consistent with the in vivo results, we observed that cultivation of melatonin inhibited the proliferation of *Alistipes Inops* in a dose‐dependent manner (Figure 5F). The above results suggest that melatonin reshapes the gut microbiota in ovariectomized mice, especially inducing a reduction in the abundance of *Alistipes Inops*.

### 2.6 Melatonin Attenuates OVX‐Induced Disturbance of Tryptophan‐5‐HT Metabolism in a Microbiota‐Dependent Manner

**Figure 6:**
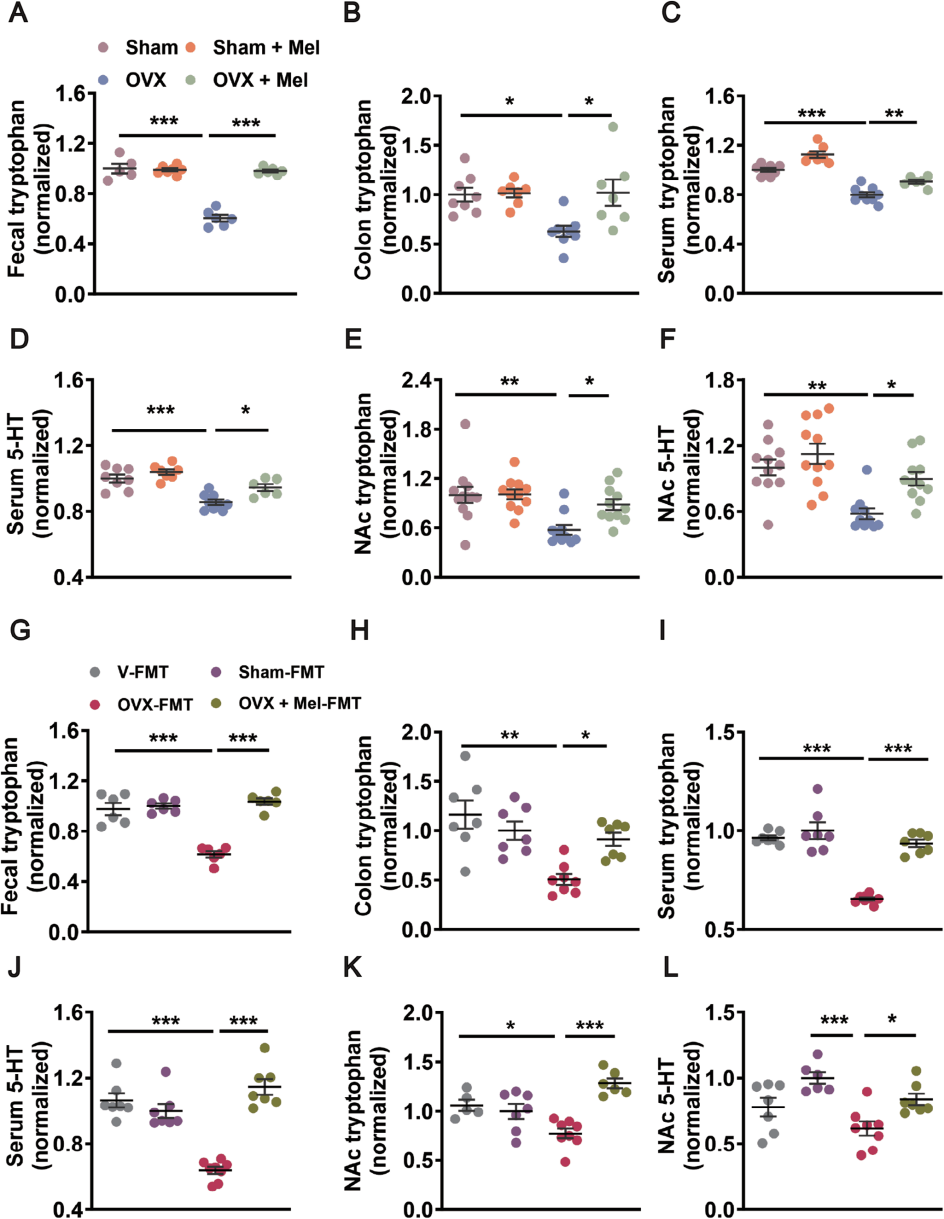
Melatonin attenuates OVX‐induced disturbance of tryptophan‐5‐HT metabolism in a microbiota‐dependent manner. A) The expression of fecal tryptophan was measured by ELISA of Sham mice, Sham + Mel mice, OVX mice, and OVX + Mel mice. *n* = 5–7 per group. B) The expression of colonic tryptophan was measured by ELISA. *n* = 7–8 per group. C) Serum level of tryptophan was analyzed by ELISA. *n* = 6–9 per group. D) Serum level of 5‐HT was analyzed by ELISA. *n* = 6–9 per group. E) ELISA quantification of tryptophan protein level in NAc normalized to controls. *n* = 10–12 per group. F) ELISA quantification of 5‐HT protein level in NAc normalized to controls. *n* = 10–11 per group. G) The expression of fecal tryptophan was measured by ELISA of V‐FMT mice, Sham‐FMT mice, OVX‐FMT mice, and OVX + Mel‐FMT mice. *n* = 6 per group. H) The expression of colonic tryptophan was measured by ELISA. *n* = 7–8 per group. I) Serum level of tryptophan was analyzed by ELISA. *n* = 7–8 per group. J) Serum level of 5‐HT was analyzed by ELISA. *n* = 7–8 per group. K) The level of tryptophan in the NAc was measured by ELISA. *n* = 5–8 per group. L) ELISA quantification of 5‐HT protein level in the NAc normalized to controls. *n* = 6–8 per group. All of the data are the mean ± SEM and analyzed by two‐way ANOVA (A–F) or one‐way ANOVA (G–L) with Bonferroni's post‐hoc test, *p < 0.05, **p < 0.01, ***p < 0.001.

*Alistipes Inops* has been demonstrated to degrade tryptophan and produce indole in vitro.^[^ ^38^ ^]^ Serotonin, also known as 5‐hydroxytryptamine (5‐HT), is a metabolite of tryptophan and is reported to modulate mood, anxiety, behavior, and other important functions of the central nervous system.^[^ ^39^ ^]^ Given that OVX induced an increase in the abundance of *Alistipes Inops*, we asked whether the tryptophan‐5‐HT pathway was involved in the antidepressant effects of melatonin on the OVX model of depression. The enzyme‐linked immunosorbent assay (ELISA) results showed that exposure to OVX decreased the level of tryptophan in the feces and colon, which were blocked by exogenous melatonin administration (**Figure** **6**A,B). Since microbes and their products can translocate across the gut barrier into circulation, the serum levels of tryptophan and 5‐HT were measured. The results showed that the levels of tryptophan and 5‐HT were decreased in the serum of OVX‐operated mice, which were reversed by melatonin treatment (Figure 6C,D), suggesting that supplementation with melatonin restored OVX‐induced impairment of peripheral tryptophan‐5‐HT metabolism. Considering that tryptophan transports across the blood‐brain barrier and converts to 5‐HT, the levels of tryptophan and 5‐HT were measured in the brain. It was found that when exposed to OVX, the levels of tryptophan and 5‐HT proteins were decreased in the nucleus accumbens (NAc), which was reversed by melatonin treatment (Figure 6E,F). Recent studies have found that CRS and unpredictable chronic mild stress induce tryptophan metabolic disorder in the medial prefrontal cortex (mPFC) and hippocampus.^[^ ^40^, ^41^ ^]^ We next measured the levels of tryptophan and 5‐HT in these brain regions that contribute to depression symptomatology. The levels of tryptophan and 5‐HT were statistically unchanged in the mPFC and hippocampus following OVX operation (Figure S3A–D, Supporting Information). Similarly, melatonin supplementation failed to affect these levels. The tryptophan‐kynurenine pathway has been shown to be activated, which is associated with psychiatric diseases such as depression.^[^ ^42^ ^]^ However, we failed to observe statistically significant differences in kynurenine levels of serum, mPFC, hippocampus, and NAc among the four groups (Figure S4A–D, Supporting Information). The results indicate that OVX impairs systemic tryptophan‐5‐HT metabolism, which is reversed by the exogenous administration of melatonin.

Chronic exposure to stress, including adolescent stress, chronic unpredictable mild stress (CUMS), CRS, and chronic social defeated stress (CSDS), reduces synaptic plasticity markers and dendritic spine density.^[^ ^43^, ^44^ ^]^ In our study, no significant differences were observed in postsynaptic density protein 95 (PSD95) protein levels in the mPFC in any group. Ovariectomized mice exhibited a slight decline in brain‐derived neurotrophic factor (BDNF) protein levels in the mPFC compared with that in the sham group (Figure S5A, Supporting Information). No statistical differences were observed in PSD95 and BDNF protein levels in the hippocampus among the four groups (Figure S5B, Supporting Information). Total dendritic spines were slightly reduced in the mPFC of ovariectomized mice (Figure S5C,D, Supporting Information). Stubby spines and mushroom spines were comparable among the four groups, while a slight decrease in thin spines was found (Figure S5E–G, Supporting Information). In the hippocampus, we observed no significant morphological differences in the dendritic spines between the sham and OVX groups with or without melatonin treatment (Figure S5H, Supporting Information). In addition, the densities of the total and three primary spine classes (stubby, mushroom, and thin) were comparable among the four groups (Figure S5I–L, Supporting Information). Collectively, OVX slightly impairs dendritic spine density in the mPFC but not in the hippocampus.

To identify whether the role of melatonin on tryptophan‐5‐HT metabolism in ovariectomized mice was associated with the gut microbiome, fecal samples were profiled after FMT. It was shown that after transplantation of microbiota from OVX‐operated mice, the levels of tryptophan were reduced in the feces and colon, which was rescued by FMT from sham or melatonin‐treated OVX‐operated mice (Figure 6G,H). The changes in serum tryptophan and 5‐HT levels were similar to those in the feces and colon (Figure 6I,J). We found that the levels of tryptophan and 5‐HT were decreased in the NAc of mice receiving FMT from ovariectomized donors, which were reversed in the NAc of mice receiving FMT from ovariectomized donors treated with melatonin (Figure 6K,L). These data indicate that melatonin reverses tryptophan‐5‐HT metabolic disorders in ovariectomized mice through a gut microbiome‐dependent mechanism.

### 2.7 Melatonin Produces Antidepressant Action in *Alistipes Inops*‐Colonized Mice by Restoring Tryptophan Metabolism

**Figure 7:**
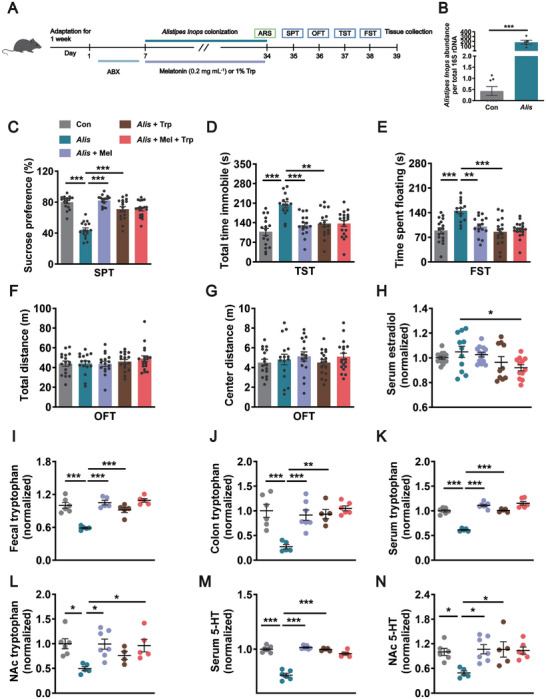
Melatonin produces antidepressant action in *Alistipes Inops*‐colonized mice by restoring tryptophan‐5‐HT metabolism. A) Schematic representation of *Alistipes Inops* colonization paradigm for female mice. B) The graph represents the *Alistipes Inops* levels in the fecal samples collected from the colon of Con and *Alis* groups of mice. *n* = 5–7 per group. C) The sucrose‐in‐water ratio in the SPT of Con mice, *Alis* mice, *Alis* + Mel mice, *Alis* + Trp mice, and *Alis* + Mel + Trp mice. *n* = 16–19 per group. D) Total time immobile in the TST. *n* = 14–19 per group. E) The floating time in the FST. *n* = 14–19 per group. F) Total distance during OFT. *n* = 16–19 per group. G) Distance spent in the center zone during OFT. *n* = 16–19 per group. H) Serum level of estradiol was analyzed by ELISA. *n* = 10–13 per group. I) Fecal level of tryptophan was analyzed by ELISA. *n* = 5–6 per group. J) Colonic level of tryptophan was analyzed by ELISA *n* = 5–7 per group. K) Serum level of tryptophan was analyzed by ELISA *n* = 5–7 per group. L) The level of tryptophan in the NAc was measured by ELISA. *n* = 4–7 per group. M) Serum level of 5‐HT was analyzed by ELISA. *n* = 5–7 per group. N) ELISA quantification of 5‐HT protein level in the NAc normalized to controls. *n* = 5–7 per group. All of the data are the mean ± SEM and analyzed by Student's *t*‐test (B) or one‐way ANOVA (C–N) with Bonferroni's post‐hoc test, *p < 0.05, **p < 0.01, ***p < 0.001. Con: naïve female mice received sterile saline treatment, *Ali*: *Alistipes Inops*‐colonized mice, *Ali* + Mel: *Alistipes Inops*‐colonized mice treated with melatonin, *Ali* + Trp: *Alistipes Inops*‐colonized mice treated with tryptophan, *Ali* + Mel + Trp: *Alistipes Inops*‐colonized mice treated with melatonin and tryptophan.

Considering that OVX increased the abundance of *Alistipes Inops*, we asked whether the alteration of *Alistipes Inops* was involved in OVX‐induced depressive‐like behaviors. After ABX treatment, female mice were administrated by gavage with *Alistipes Inops* or vehicle. Then, *Alistipes Inops*‐colonized mice were given melatonin or fed synthetic chows containing 1% tryptophan (**Figure** **7**A). We observed that the abundance of *Alistipes Inops* was increased by gavage administration of *Alistipes Inops* (Figure 7B). The *Alistipes Inops*‐colonized mice exhibited less sucrose consumption in the sucrose preference test than that in control mice, which was reversed by melatonin supplementation or tryptophan treatment (Figure 7C). Meanwhile, administration with *Alistipes Inops* induced depressive‐like behaviors in naïve mice, as shown by increased immobility time in the tail suspension test and forced swim test (Figure 7D,E). Melatonin supplementation or tryptophan treatment reversed *Alistipes Inops* colonization‐induced depressive‐like behaviors, with a decreased immobility time in both the tail suspension test and forced swim test (Figure 7D,E). No statistical differences were shown in locomotor activity and center distance in the open field test among the five groups (Figure 7F,G).

To exclude the effect of *Alistipes Inops* colonization on estradiol, we next measured the level of serum estradiol after *Alistipes Inops* colonization. There were no differences in the level of serum estradiol among control and *Alistipes Inops‐*colonized mice and the mice supplemented with tryptophan or melatonin alone. However, dietary tryptophan combined with melatonin reduced serum estradiol levels in *Alistipes Inops*‐colonized mice (Figure 7H), indicating that the combination of tryptophan and melatonin produces an impact on the level of estradiol. These results suggest that *Alistipes Inops* colonization induces depressive‐like behaviors, and melatonin supplementation or tryptophan treatment is sufficient to ameliorate depressive‐like behaviors induced by *Alistipes Inops* colonization independent of estrogen.

To understand the role of tryptophan‐5‐HT metabolism in the context of *Alistipes Inops* colonization as well as melatonin supplementation or dietary tryptophan treatment, the levels of tryptophan in feces, colon, serum, and NAc were measured. The levels of tryptophan were decreased in feces and colon of *Alistipes Inops*‐colonized mice. When *Alistipes Inops*‐colonized mice were treated with either melatonin or tryptophan, the levels of tryptophan were recovered to normal levels (Figure 7I,J). Similar results were observed in the serum and NAc (Figure 7K,L). Accordingly, the levels of 5‐HT were decreased in the serum and NAc of *Alistipes Inops*‐colonized mice, which was rescued by melatonin or dietary tryptophan treatment (Figure 7M,N). However, there were no significant synergistic effects on tryptophan metabolism when combination of melatonin and tryptophan in *Alistipes Inops*‐colonized mice. Collectively, these results indicate that melatonin reverses the disturbance of tryptophan‐5‐HT metabolism induced by *Alistipes Inops* colonization, which contributes to the antidepressant effects.

## 3 Discussion

Our study provided evidence that melatonin exhibited antidepressant effects in OVX‐operated mice by restoring the homeostasis of gut microbes and normalized systemic tryptophan‐5‐HT metabolism. Specifically, we found that melatonin protected against OVX‐induced depressive‐like behaviors by inhibiting the growth of *Alistipes Inops* in the gut. Furthermore, melatonin prevented the disturbance of tryptophan‐5‐HT metabolism and depressive‐like behavior induced by *Alistipes Inops*. Our results identified the previously unrecognized role of *Alistipes Inops* in OVX‐induced behavioral abnormality. Overall, the present study highlights the antidepressant effects of melatonin on ovariectomized mice, possibly associated with the inhibition of *Alistipes Inops*. Accordingly, melatonin or other therapies targeting the specific gut commensal bacterium *Alistipes Inops* may pave the way for the treatment of menopausal depression.

Most studies have shown that menopausal depression is associated with lower basal hormone concentrations.^[^ ^45^, ^46^ ^]^ Recent findings support another plausible mechanism that the hypothalamic‐pituitary‐adrenal axis dysfunction induced by fluctuations in ovarian hormones increases sensitivity to stress during the menopause transition.^[^ ^47^ ^]^ Emerging evidence hints that inflammation and gut microbiota may be involved in the modulation of depressive‐like behaviors in mouse models of menopausal depression.^[^ ^6^, ^32^ ^]^ However, due to the complex hormonal milieu of the menopause transition, the mechanisms of menopausal depression are poorly understood. Here, we used OVX operation, which mimics estrogen deficiency, to induce depressive‐like behaviors in mice. In addition, we found a decline in serum estradiol and dysbacteriosis in OVX‐operated mice. The impairment of the gut barrier was observed in OVX‐rat or mice under CRS,^[^ ^25^, ^26^ ^]^ prompting that the disruption of the gut barrier may contribute to OVX‐induced behavioral despair. However, there were no changes in the level of tight junction proteins and inflammatory cytokines in OVX‐operated mice, suggesting that the disruption of the gut barrier, at least in our study, is not the cause of OVX‐induced depressive‐like behaviors. Several studies have proved the beneficial effects of *Akkermansia muciniphila* on the gut barrier integrity and function.^[^ ^48^, ^49^, ^50^ ^]^ In our study, considering the higher abundance of *Akkermansia muciniphila* in OVX‐operated mice, it is possible that *Akkermansia muciniphila* and its membrane protein maintain the gut barrier integrity. Additional studies are required to comprehend the complete mechanisms underlying the gut barrier integrity in the context of OVX. Studies have reported that synaptic plasticity markers and dendritic spine density are decreased in mouse models of depression, such as CSDS, CRS, and chronic mild stress.^[^ ^44^, ^51^, ^52^ ^]^ Consistent with the above studies, we observed a slight decrease in the BDNF protein level in mPFC of OVX‐operated mice. Furthermore, exposure to OVX induced an insignificant decrease in the density of total dendritic spines in mPFC, which mainly resulted from the reduction in the density of thin spines. Nevertheless, the stress‐induced decreased dendritic spine density in the hippocampus was not observed under the OVX condition. This may be related to differences in paradigms of menopausal depression or to the higher abundance of *Akkermansia muciniphila* in OVX‐operated mice that prevents synaptic loss in the hippocampus.^[^ ^53^ ^]^ Our results prove that the plasticity of dendritic spine structure is unlikely to be a major component in the cause of OVX‐induced behavioral disorders.

For menopausal depression, gonadal hormone replacement therapy is the most commonly used treatment. However, the effects of gonadal hormone replacement therapy for women with menopausal depression remain controversial. Several studies show that 17β‐estradiol fails to relieve depressive symptoms in postmenopausal women,^[^ ^54^ ^]^ while a recent clinical trial found that a 12‐month administration of transdermal 17β‐estradiol prevented depressive symptoms among euthymic perimenopausal and early postmenopausal women.^[^ ^55^ ^]^ Meanwhile, gonadal hormone replacement therapy may increase the risk of cardiovascular and cerebrovascular diseases and breast cancer in menopausal women.^[^ ^56^ ^]^ Except for hormone therapy, selective serotonin reuptake inhibitors (SSRIs) treatment and cognitive behavioral therapy are also widely recommended.^[^ ^57^ ^]^ However, SSRIs might be effective in the short term,^[^ ^58^ ^]^ and the effects of cognitive behavioral therapy vary with different individuals.^[^ ^59^ ^]^ Thus, it urgently needs to discover an effective therapy for menopausal depression. Melatonin, a derivative of the amino acid tryptophan, has been shown to improve the mood of perimenopausal women.^[^ ^13^ ^]^ In addition, melatonin produces antidepressant effect in chronic corticosterone‐ and lipopolysaccharide‐treated mice.^[^ ^15^, ^60^ ^]^ Our results showed that a 28‐day oral administration with melatonin (0.2 mg mL^−1^) rescued behavioral abnormalities in ovariectomized mice without affecting weight gain. Although the antidepressant effects of other pharmaceuticals on ovariectomized animals are believed to raise the level of estradiol, our data suggest that melatonin produces antidepressant effects that do not rely on the restoration of estradiol. Intriguingly, the colonic level of melatonin seems to decrease in the context of OVX, which could be reversed by exogenous melatonin supplementation. The host colonic chromaffin cells and gut microbiota contribute to the production of intestinal melatonin.^[^ ^61^, ^62^ ^]^ In addition, depletion of gut microbiota in naïve female mice led to lower colonic levels of melatonin, suggesting the gut microbiota directly impact the local mucosal melatonin. We next found that FMT from adolescent female mice rescued the decline in colonic melatonin expression induced by OVX. Thus, microbiota under steady‐state conditions may either promote melatonin synthesis in colonic chromaffin cells or possess the capacity to produce melatonin. The precise role of local mucosal melatonin that is altered with the gut microbiota in the pathogenesis of menopausal depression should be investigated in future studies. On the other hand, exogenous melatonin treatment did not affect the level of serum melatonin, indicating that the antidepressant effects of exogenous melatonin are independent of direct impacts on the brain.

Emerging evidence has indicated that the gut microbiota may be involved in the pathology of depressive disorders through the “microbiota‐gut‐brain axis”.^[^ ^20^, ^63^ ^]^ The gut microbiota, as a potential contributor to anxiety‐like behavior, was previously implicated in several studies.^[^ ^64^ ^]^

In addition, ABX‐induced microbiome depletion contributes to resilience to anhedonia in mice subjected to CSDS.^[^ ^65^ ^]^ On the contrary, Sudo et al. demonstrated that mice absent with gut microbiome show an enhanced response to stress.^[^ ^66^ ^]^ Studies also found an increased immobility time of the tail suspension test and forced swim test or a reduction in social activity of specific pathogen‐free mice following ABX treatment.^[^ ^67^, ^68^ ^]^ Although no statistical differences were shown in immobility time in the tail suspension test of sham mice treated with or without ABX, we observed that ABX treatment reduced sucrose preference and increased immobility time in the forced swim test of sham mice. It seems that ABX treatment leads to anhedonia and behavioral despair in sham mice. CSDS mice treated with ABX exhibit higher levels of species *Lactobacillus murinus*, which is the crucial producer of short‐chain fatty acid (SCFA).^[^ ^65^ ^]^ The higher levels of SCFA may contribute to the resilient effects of ABX on CSDS model. However, ABX treatment decreases *Lachnospiraceae* and *Ruminococcaceae*, which are fiber‐degrading and SCFA producers.^[^ ^67^, ^69^ ^]^ ABX‐mediated dysbiotic microbiota could exert opposite effects on depressive symptoms, which depends on the dominant bacteria and its metabolite. Thus, the reduction in SCFA level induced by decreased SCFA‐producing bacteria may lead to behavioral despair in sham mice. It may be necessary to employ germ‐free mice to avoid ABX‐induced microbiota perturbation to result in behavioral alterations. Additionally, recent publications have shown that ABX treatment abolishes the beneficial effects of melatonin in some diseases, such as sleep restriction and liver inflammation.^[^ ^29^, ^70^ ^]^ Consistent with the above findings, our study demonstrated that depletion of gut microbiota led to abrogation of the antidepressant effects of melatonin. FMT has been regarded as a potential strategy to relieve symptoms of psychiatric illnesses.^[^ ^71^ ^]^ We confirmed that recipient mice receiving gut microbiota from OVX‐operated donor mice or melatonin‐treated OVX‐operated donor mice exhibited similar phenotypes to donors, respectively. The findings further suggest that the antidepressant effects of melatonin depend on the gut microbiota. Indeed, we found that the gut microbiota from adolescent female mice ameliorated depressive‐like behaviors induced by OVX, indicating FMT from adolescent women donors may be developed into a potential therapy for menopausal depression. Based on our 16S rDNA sequencing, the higher abundance of *Alistipes Inops* was observed in OVX‐operated mice, aligning with previous studies that the higher abundance of *Alistipes* is associated with mental signs of depression.^[^ ^33^ ^]^ We, therefore, reasoned that *Alistipes Inops* may have the capacity to induce depressive‐like behaviors. Consistent with our hypothesis, naïve female mice inoculated with *Alistipes Inops* exhibited depressive‐like behaviors. Furthermore, our in vivo and in vitro experiments revealed that melatonin produced antidepressant effects by selectively inhibiting the proliferation of *Alistipes Inops*. Besides *Alistipes Inops*, some other bacterial species, including *Akkermansia muciniphila*, are also highly abundant in ovariectomized mice. An increase in the abundance of *Akkermansia muciniphila* can possibly result from acute restraint stress.^[^ ^40^ ^]^ Thus, reducing the abundance of *Alistipes Inops* by melatonin treatment or other pharmacological interventions may be helpful for treating menopausal depression.

Several studies have demonstrated that chronic stress disrupts the gut microbiota and the related metabolites, especially tryptophan.^[^ ^41^, ^72^ ^]^ The promotion of tryptophan hydroxylase by bacterial colonization has been proven to ameliorate depressive‐like behaviors induced by CRS.^[^ ^73^ ^]^ Furthermore, the utilization of tryptophan improves neuroinflammation in mouse models of CUMS.^[^ ^74^ ^]^ However, the precise mechanisms by which microbiota‐associated tryptophan metabolite modulates depressive‐like behaviors induced by OVX remain unclear. We observed that OVX induced the deficiency of tryptophan metabolism and confirmed the antidepressant effects of tryptophan on behavior abnormality induced by bacterial colonization. Since the translocation of tryptophan from intestine to circulation is required for 5‐HT availability in the brain,^[^ ^75^ ^]^ and various chronic stress exposure leads to tryptophan‐5‐HT metabolic disorder,^[^ ^41^, ^76^ ^]^ we assumed that reduced systemic tryptophan would lead to lower 5‐HT level in the brain. We found a decline of tryptophan and 5‐HT in NAc of ovariectomized mice. However, the levels of tryptophan and 5‐HT altered neither in mPFC nor in the hippocampus. These results may be due to the reduced blood‐brain barrier integrity in NAc under stress.^[^ ^77^ ^]^ Previous studies highlight the role of kynurenine metabolic pathway in depressive‐like behaviors.^[^ ^40^, ^78^ ^]^ The systemic kynurenine pathway is activated in mouse models of CRS. Nevertheless, we did not detect statistically significant alteration of kynurenine level in serum, mPFC, hippocampus, or NAc. Our data demonstrate an impairment of 5‐HT availability induced by OVX, pointing toward a depression mechanism that occurs in a kynurenine pathway‐independent approach but is likely dependent on the 5‐HT pathway. We revealed that a decline of systemic tryptophan in ovariectomized mice may be largely induced by the increased abundance of *Alistipes Inops*, a species within the Bacteroidetes. A previous study has reported that *Alistipes Inops* is an indole‐positive organism,^[^ ^38^ ^]^ and thus decreases serotonin availability by degraded tryptophan. We also provided evidence that the administration of tryptophan or melatonin was sufficient to rescue the deficiency of tryptophan metabolism in *Alistipes Inops*‐colonized mice. Recent studies have provided evidence that indole, a tryptophan catabolite, can produce indole‐3‐carbinol (I3C).^[^ ^79^ ^]^ In a study of 17 subjects, a lower level of estradiol was observed after I3C treatment.^[^ ^80^ ^]^ In our study, *Alistipes Inops*‐colonized mice exhibited a decline in serum estradiol after combining treatment with melatonin and tryptophan. The decline of serum estradiol is possibly due to the production of I3C by gut microbiota after melatonin and tryptophan combined treatment. Consistent with our hypothesis, we found that *Alistipes Inops* colonization was sufficient to reduce serum I3C level, whereas melatonin treatment combined with tryptophan reversed the serum I3C level in *Alistipes Inops*‐colonized mice (Figure S6A, Supporting Information). It supports that lower serum estradiol level is associated with higher serum I3C level in the context of *Alistipes Inops* colonization. Further studies will need to uncover the mechanism underlying the down‐regulation effect of I3C on estradiol.

Due to the contribution of multiple microbial strains to the homeostasis of tryptophan metabolism, it is possible that the colonization of tryptophan‐producing microbes may reverse OVX‐induced behavioral phenotypes and normalize the deficiency of tryptophan metabolism. In our study, *Alistipes Inops* has been verified as the pathogenic microbiota. We proposed that the administration of tryptophan or melatonin, instead of other tryptophan‐producing microbes, maybe a convenient treatment for relieving depression‐related symptoms. Although *Alistipes Inops* is positive for indole production, the enzyme encoded by *Alistipes Inops* is required to identify it. Engineered phages targeting *Alistipes Inops* or the specific enzyme inhibitor may provide therapeutic potential for menopausal depression. However, further studies are necessary to determine whether microbe‐based treatments can be effective and safe for the clinical alleviation of depressive symptoms during menopause.

## 4 Conclusion

**Figure 8:**
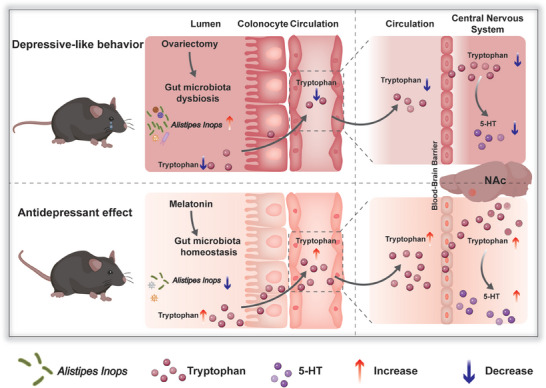
Schematic illustration of the antidepressant effect of melatonin on OVX model of depression. An increase in the abundance of *Alisipes Inops* led to the degradation of intestinal tryptophan, which destroyed the systemic tryptophan‐5‐HT availability and thus induced depressive‐like behaviors. Melatonin supplementation restored systemic tryptophan‐5‐HT metabolic disorders by suppressing the growth of *Alistipes Inops*, which ameliorated depressive‐like behaviors.

Collectively, our findings highlight the previously unrecognized role of *Alistipes Inops*‐mediated tryptophan metabolism in gonadal hormone‐related psychiatric disorders. Melatonin or other drugs that can induce inhibition of *Alistipes Inops* could provide promising new therapeutic possibilities for menopausal depression (**Figure** **8**). Future research will be necessary to aim at determining the optimal dose of melatonin in patients with menopausal depression and at developing novel therapeutic agents to inhibit *Alistipes Inops*.

## 5 Experimental Section

### Animals

Female C57BL/6J mice were obtained from the Animal Center of Hunan SilaikeJingda Laboratory Animal Corporation Ltd (Changsha, Hunan, China) and were kept under specific pathogen‐free conditions, with 24 h access to irradiated food and sterile water at temperatures between 20 and 24 °C, humidity between 20%–40%, and a 12 h light‐dark cycle. Animal work was carried out under the National Institutes of Health Guide for the Care and Use of Laboratory Animals, with local ethical review by the Review Committee for the Use of Human or Animal Subjects of Huazhong University of Science and Technology

### Animal Model

Seven to eight‐week‐old female C57BL/6J mice were randomly assigned to the sham or OVX group. Mice were anesthetized with pentobarbital sodium (45 mg kg^−1^, intraperitoneal injection). A midline, longitudinal incision was made in the skin of the lower lumbar region, and transverse muscle incisions were made directly over both ovaries. Sham surgery consisted of a mid‐ventral abdominal incision through the skin and removal of the same amount of adipose tissue. The mice were exposed to acute restraint stress after OVX for 4 weeks, in which the mice were placed inside 50 mL cylindrical plastic tubes, with small holes in both sides for breathing for 5 h.

### Drug Administration

Melatonin (# 211835; AJ&K Scientific, Beijing, China) was dissolved in sterile water containing 0.065% absolute ethanol. After OVX, mice were administrated with melatonin (0.05, 0.1, 0.2, or 0.4 mg mL^−1^) for 28 consecutive days. The melatonin solution was replaced twice a week. antibiotic cocktails (ABX) were used to deplete gut microbiota. Mice were given ABX containing ampicillin (0.5 g L^−1^; # MB1378; Meilunbio, Dalian, China), streptomycin (1 g L^−1^; # MB1275; Meilunbio, Dalian, China), gentamicin (1 g L^−1^; # MB1331; Meilunbio, Dalian, China) and vancomycin (0.5 g L^−1^; # MB1260; Meilunbio, Dalian, China) in the drinking water for seven consecutive days.

### Fecal Microbiota Transplantation (FMT)

For FMT from adolescent female mice, recipient mice were pre‐treated with ABX for 7 consecutive days and subjected to OVX. Before FMT, the ABX was replaced by sterile tap water. Fresh fecal pellets were collected directly from the adolescent female mice. Feces (100 mg) were homogenized in 1 mL of sterile saline using a vortex followed by centrifugation for 5 min at 1000 rpm at 4 °C. The supernatant was transferred into a new tube and then centrifuged for 5 min at 12 000 rpm at 4 °C. Finally, the bacterial pellet was resuspended in 1 mL of sterile saline. Recipient mice were colonized with microbiota by oral gavage at a volume of 200 µL. The control group received an equal volume of sterile saline. FMT was carried out for three consecutive days weekly for 4 weeks.

To investigate the role of gut microbiota in the antidepressant effect of melatonin, mice were randomly divided into donor or recipient groups. Donor mice were further divided into the sham, OVX, and OVX supplemented with melatonin (0.2 mg mL^−1^) (OVX + Mel) groups. To deplete microbiota, recipient mice were treated with ABX, as described above. The donor mice were placed into a clean cage, and feces were collected using sterile forceps. Then, feces were suspended in sterile saline, centrifuged twice, and resuspended in sterile saline. Each recipient mouse was then administrated by oral gavage with 200 µL of diluted fecal contents for three consecutive days per week. Four weeks later, mice were subjected to acute restraint stress.

### *Alistipes Inops* Culture and Treatment In Vitro

*Alistipes Inops* (DSM28863) lyophilized powder was dissolved in a compound solution under a sterile environment. The bacterial suspension was prepared and cultured in an anaerobic cabinet (10% CO_2_, 10% H_2_, and 80% N_2_) at 37 °C in Brain Heart Infusion Agar (BHI; HOPEBIO, Qingdao, China) containing 10% fetal bovine serum (FBS; Zhejiang Tianhang Biotechnology Corp, Huzhou, China) for 72 h. The single colony was transferred into BHI containing 10% FBS and anaerobically incubated at 37 °C for 72 h. Finally, the abundance of *Alistipes Inops* was determined by measurement of OD value at 600 nm. 16S rDNA identified DSM28863 as *Alistipes Inops*. To evaluate the effect of melatonin on *Alistipes Inops*, *Alistipes Inops* was treated with melatonin at 1, 2, 4, and 8 mm, respectively. About 52 h later, the growth rate of *Alistipes Inops* entered the stationary phase. Then, the absorbance of OD600 at different time points was measured within 52 h, and the growth curve of *Alistipes Inops* was plotted.

### *Alistipes Inops* Colonization

Mice were pretreated with ABX for 7 days as described above. *Alistipes Inops* colony‐forming units were diluted with sterile saline to adjust the concentration to 1 × 10^10^ colony‐forming unit mL^−1^. Each mouse was administrated by gavage with 200 µL suspension for three consecutive days per week for 4 weeks before other experiments or analyses. Control mice were administrated by gavage with sterile saline.

### Tryptophan Dietary Experiments

After *Alistipes Inops* colonization, tryptophan‐modified synthetic chows (7% fat content, 64.7% carbohydrate content, 18.8% protein content, and 1% tryptophan content) were fed to mice for 4 weeks.

### Open Field Test

The open‐field test was performed as described in the previous paper.^[^ ^81^ ^]^ The mouse was gently placed in the center of an open field cage (45 cm × 45 cm × 45 cm) and left to explore freely for 10 min. The total distance and the distance in the center zone (35 cm × 35 cm) were recorded by Anymaze software (Stoelting Co, Wood Dale, IL, USA). The total distance was measured to assess exploratory behavior and the general activity of animals. The open field test was calculated as the distance in the center zone. The box was cleaned with 75% ethanol after each test to remove olfactory cues.

### Sucrose Preference Test

The sucrose preference test was carried out as described in the published protocol with small modifications.^[^ ^81^ ^]^ Mice were singly housed before the test and were habituated to two 50 mL tubes filled with either normal drinking water or 1% sucrose solution. During the habituation period, the positions of two bottles were switched daily to avoid position preference. After habituation for 48 h, mice were deprived of water for 12 h. The bottle containing 1% sucrose solution and the bottle containing drinking water were placed for 2 h, and the solution consumed from each bottle was measured, respectively. Sucrose preference (%) was calculated as 100 × (sucrose solution consumption/total solution consumption), and the total solution consumption was calculated as sucrose solution consumption + water solution consumption.

### Tail Suspension Test

The tail suspension test was carried out as previously described.^[^ ^81^ ^]^ Briefly, mice were suspended by the tail using adhesive tape affixed 1 cm from the origin of the tail and suspended 20 cm above the floor under dim light conditions. The total time immobile of the animals was recorded by a video camera during a 6 min period. The total immobility time was evaluated in a blind manner.

### Forced Swim Test

The forced swim test was tested using a previously published protocol.^[^ ^81^ ^]^ Mice were placed individually into transparent plastic cylinders (20 cm diameter, 30 cm deep) filled to a depth of 20 cm with fresh water (25 ± 1 °C) under dim light conditions for 6 min, and the behavior of mice was recorded by Anymaze software (Stoelting Co, Wood Dale, IL, USA). The floating time during the last 4 min was evaluated in a blind manner.

### Tissue Collection

Mice were deeply anesthetized with pentobarbital sodium (45 mg kg^−1^, intraperitoneal injection). Approximately 0.5 mL of blood was taken via eyeball extraction. The blood was allowed to coagulate for 2 h at room temperature and then centrifuged at 2000 rpm for 10 min for serum collection. The mice were then decapitated, and the whole brain was removed. The nucleus accumbens (NAc), medial prefrontal cortex (mPFC), and hippocampus were dissected with a dissecting microscope. Isolated colonic tissues and the fecal samples were placed directly into an ice‐cold tube, respectively. All the tissues were immediately frozen in liquid nitrogen, and stored at −80 °C until further processing.

### Histological Staining

Sections of colon tissues were washed with phosphate‐buffered saline, fixed with 4% paraformaldehyde, and embedded with paraffin. Tissues were then subjected to hematoxylin and eosin staining.

### Enzyme‐linked Immunosorbent assay (ELISA)

The frozen tissues were lysed using ice‐cold radio‐immunoprecipitation assay buffer (Beyotime Biotechnology, Haimen, China) with the protease and phosphatase inhibitors (50 mm Tris, 150 mm NaCl, 1% Triton X‐100, 1% sodium deoxycholate, 0.1% sodium dodecyl sulfonate, protease inhibitor mixture, pH 7.4). The lysates were allowed to centrifugation at 12 000 rpm for 20 min at 4 °C, and the supernatant was transferred into a new tube. Total protein concentrations were measured by the Bicinchoninic Acid Protein Assay (BOSTER, Wuhan, China). The levels of tryptophan (# RJ22298; RENJIEBIO, Shanghai, China), serotonin (5‐hydroxytryptamine, 5‐HT; # RJ16720; RENJIEBIO, Shanghai, China), melatonin (# RJ17664; RENJIEBIO, Shanghai, China), estradiol (# RJ17016; RENJIEBIO, Shanghai, China), kynurenine (# RJ17525; RENJIEBIO, Shanghai, China) and I3C (# RJ27567; RENJIEBIO, Shanghai, China) were assessed using commercially available ELISA kits according to the manufacturer's instructions. Data were normalized based on total protein concentrations.

### Western Blotting

Tissue total proteins were obtained using the same process as above. Following the addition of 3× loading buffer (Beyotime Biotechnology, Haimen, China), samples were vortexed and heated for 10 min at 95 °C. 20 µg of the final mixture was loaded per lane on a 12% sodium dodecyl sulfate‐polyacrylamide gel electrophoresis. Subsequently, the proteins were transferred to a nitrocellulose membrane (Merck Millipore, Billerica, USA). For immunodetection, the membranes were incubated overnight at 4 °C with primary antibodies: anti‐PSD95 (# ab18285, 1:1000; Abcam, Cambridge, MA, USA), anti‐BDNF (# ab108319, 1:1000; Abcam, Cambridge, MA, USA), β‐actin (# sc‐47778, 1:2000; Santa Cruz Biotechnology, California, USA). The membranes were washed with tris‐buffered saline and incubated with secondary antibodies (1:10000, LI‐COR, Lincoln, USA) for 2 h at room temperature. The protein stain image was visualized and quantified on the Odyssey Imaging System (LI‐COR Biosciences, Lincoln, USA).

### Quantitative Reverse Transcription‐Polymerase Chain Reaction (qRT‐PCR)

RNA was extracted with TRIzol reagent (Invitrogen, Carlsbad, CA), and quality was determined using the ultrafine ultraviolet spectrophotometer (NanoUV‐3000, Thermo Fisher Scientific, Massachusetts, USA). RNA was reverse‐transcribed to cDNA using the PrimeScript II 1st Strand cDNA Synthesis Kit (Takara Bio Inc, Kusatsu, Japan). For qRT‐PCR, the target genes were quantified and normalized to the housekeeping gene GAPDH using the 2^−ΔΔCT^ method. The primer sequences used in qRT‐PCR were listed in Table S1, Supporting Information.

### Microbial DNA Extraction and Amplification

The fresh stool samples from mice were collected and stored at −80 °C. Fecal DNA was extracted using the TIANamp Stool DNA kit (Tiangen, Beijing, China) according to the manufacturer's instructions. The fecal DNA was then subjected to quantification of *Alistipes Inops* using qRT‐PCR. Universal bacteria were used as an endogenous control to normalize the expression data using the 2^−ΔΔCT^ method. The primer sequences used in qRT‐PCR were listed in Table S1, Supporting Information.

### 16S rDNA Sequencing

Frozen fecal samples were kept in a freezer at −80 °C for further analyses. Total microbial genomic DNA was extracted from fecal samples using the E.Z.N.A. soil DNA Kit (Omega Bio‐tek, Norcross, GA, U.S.) according to the manufacturer's instructions. The V3‐V4 hypervariable region of 16S rDNA‐encoding genes was amplified with primer pairs 338F and 806R by an ABI GeneAmp 9700 PCR thermocycler (ABI, CA, USA). Extracted from a 2% agarose gel, the PCR product was purified using the AxyPrep DNA Gel Extraction Kit (Axygen Biosciences, Union City, CA, USA) and purified using the AxyPrep DNA Gel Extraction Kit (Axygen Biosciences, Union City, CA, USA). By Quantus Fluorometer (Promega, USA), the PCR product was purified. The purified amplicons were analyzed using an Illumina MiSeq PE300 platform (Illumina, San Diego, USA) with standard protocols of Majorbio Bio‐Pharm Technology Co. Ltd. (Shanghai, China). The taxonomy of each operational taxonomic unit's (OTU) representative sequence was assigned by RDP Classifier version 2.2 against the 16S rDNA gene database (SILVA138) using a confidence threshold of 0.7. Alpha diversity indices including observed OTUs, Abundance‐based Coverage Estimator (Ace) richness, Sobs index, and Shannon‐Wiener (Shannon) index were calculated with Mothur v1.30.1. Similarities among the microbial communities in different samples were determined by principal coordinates analysis (PCoA) and Non‐metric Multidimensional Scaling (NMDS) based on Bray‐Curtis dissimilarity using the Vegan v2.5‐3 package. Other analyses were conducted on the (https://cloud.majorbio.com) platform.

### Morphological Analysis of Dendritic Spine

Mice were intracranially injected with designated AAV (AAV‐GFP, Genechem Co., Ltd, Shanghai, China) into mPFC and hippocampus. One‐month post‐AAV injection, mice were anesthetized with pentobarbital sodium (45 mg kg^−1^, intraperitoneal injection) and immediately transcardially perfused with 0.9% saline followed by 4% paraformaldehyde in phosphate‐buffered saline. After perfusion, the brain was dissected out and subjected to post‐fixation for 24 h. Fixed brain tissues were further dehydrated using 10%–30% sucrose solution for 72 h at 4 °C. Coronal sections (55 µm) were prepared using a freezing microtome (CM1900, Leica Microsystems, Wetzlar, Germany). Each group contained three mice and three brain slices were randomly selected from each mouse. To analyze the dendritic spine, images were captured using a confocal microscope (FV1000, Olympus, Tokyo, Japan) and 100× objective lens in z‐stack mode at 0.5‐µm intervals. Spine density, spine length, and spine classification were analyzed with Imaris software (Bitplane). The rules for spine classification: 1) Stubby spines: the spine length was <1 µm long and head width/neck width was <1.5; 2) Mushroom spines: head width/neck width was over 1.5 and 3) Thin spine: the spine length was over 1 µm long and head width/neck width was <1.5. The number of spines was normalized based on per micrometer of dendritic length.

### Statistical Analysis

Statistical analysis was carried out using GraphPad Prism 9 software. The difference between the two groups was determined by a two‐tailed unpaired Student's *t*‐test. One‐way analysis of variance (ANOVA) or Kruskal‐Wallis test was used among three independent groups, and two‐way ANOVA or three‐way ANOVA was used for comparisons of multiple factors, with normality distribution assumed followed by Bonferroni's post‐hoc test. Results were graphed as mean ± SEM and the threshold level of significance was identified at *p* < 0.05. The sample size was based on similar, previously established experiments or was calculated using power analyses to estimate the number of replicates required. Details were provided in Table S4, Supporting Information.

## Conflict of Interest

The authors declare no conflict of interest.

## Author Contributions

K.Y.Z. and B.G. contributed equally. This project was conceived by K.Y.Z., F.W., and J.G.C; K.Y.Z., F.W., and J.G.C. designed the experiments; K.Y.Z. performed most of the experiments; B.G. collected the tissues and analyzed the data; H.J.W. assisted in collecting tissue. K.Y.Z. wrote the manuscript; J.G.H., H.S.C., Z.L.H. and L.H.L. provide technological guidance; F.W. and J.G.C. edited the manuscript.

## Supporting information

Supporting Information

## Data Availability

The data that support the findings of this study are available from the corresponding author upon reasonable request.
