# Supplementary material for: Melatonin Ameliorates Depressive‐Like Behaviors in Ovariectomized Mice by Improving Tryptophan Metabolism via Inhibition of Gut Microbe Alistipes Inops
Source: Adv Sci (Weinh). 2024 Jul 8;11(34):2309473. doi: 10.1002/advs.202309473 (PMC11425877; doi:10.1002/advs.202309473)
Supplement: Supplementary file 1 — Supporting Information [file ADVS-11-2309473-s001.docx]

**Supplementary Figures**


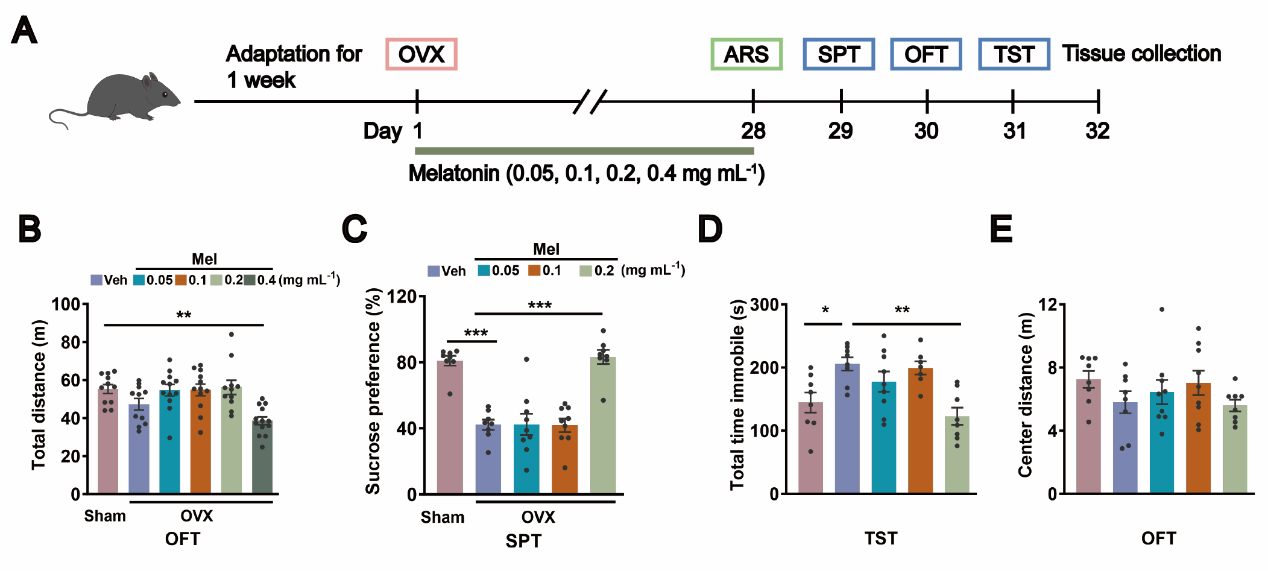


**Figure S1. The effects of different doses of melatonin on behavioral phenotypes induced by ovariectomy (OVX).** A) Schematic representation of different concentrations of melatonin administration. B) Effects of melatonin at concentrations of 0.05, 0.1, 0.2 and 0.4 mg mL^-1^ on total distance during open field test of OVX-operated mice. *n* = 11–13 per group. C) Effects of melatonin at concentrations of 0.05, 0.1, 0.2 mg mL^-1^ on the sucrose in water ratio in the sucrose preference test OVX-operated mice. *n* = 8–9 per group. D) Effects of melatonin at concentrations of 0.05, 0.1, 0.2 mg mL^-1^ on total time immobile in the tail suspension test OVX-operated mice. *n* = 7–9 per group. E) Effects of melatonin at concentrations of 0.05, 0.1, 0.2 mg mL^-1^ on distance spent in the center zone during open field test OVX-operated mice. *n* = 8–9 per group. All of data are the mean ± SEM and analyzed by one-way ANOVA (B–E) with Bonferroni’s post-hoc test, **P* < 0.05, ***P* < 0.01, ****P* < 0.001. Sham: sham-operated mice, OVX: ovariectomy-operated mice, Mel: melatonin, ARS: acute restraint stress, SPT: sucrose preference test, OFT: open field test, TST: tail suspension test. The statistical details can be found in Supplementary Table S4.

**
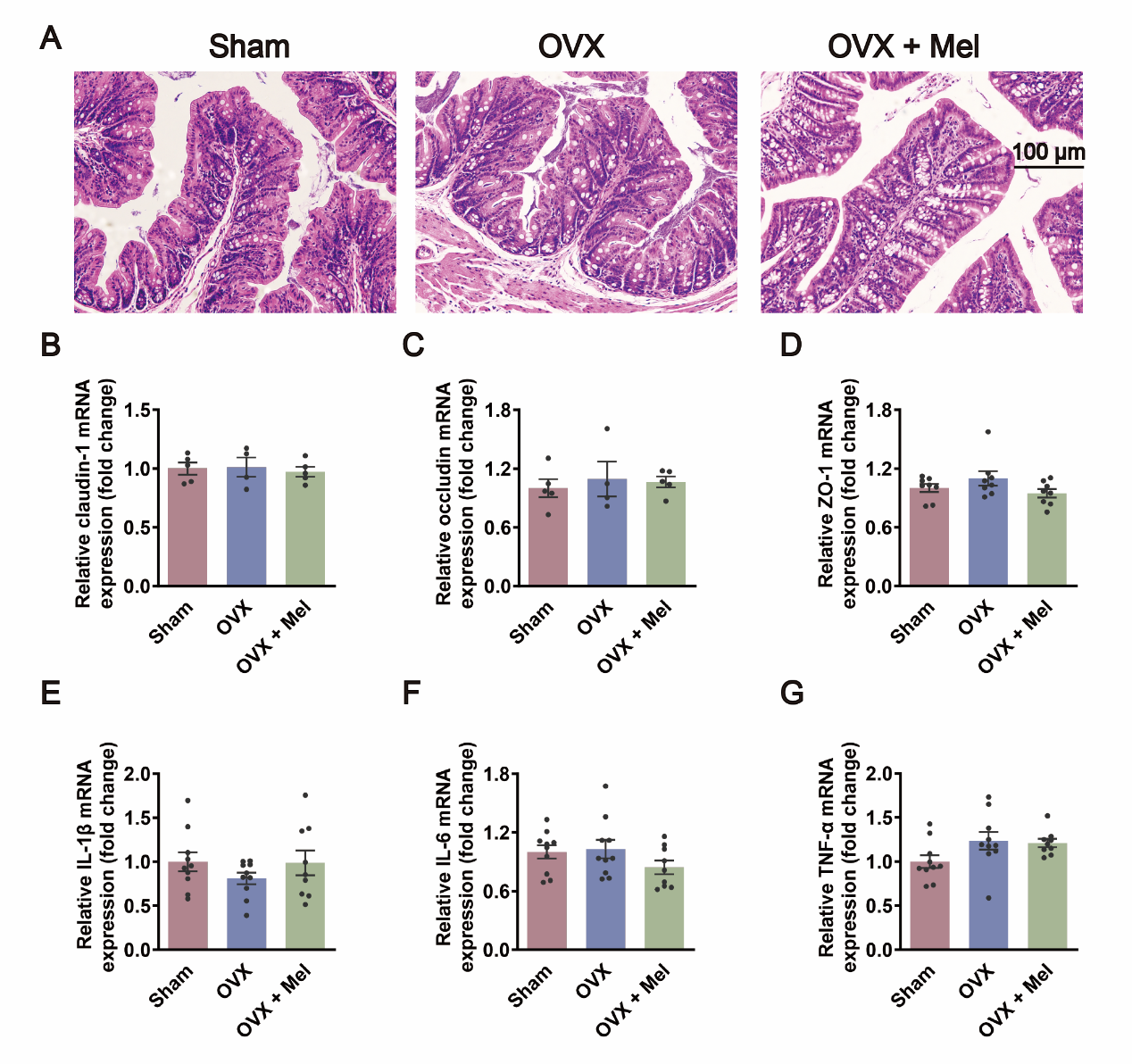
**

**Figure S2.** **OVX operation or melatonin-treated OVX operation exerts no effect on intestinal mucosal barrier function.** A) Colon tissues were collected and examined histologically. Representative images of the hematoxylin & eosin -stained colon sections. Images were taken at ×20 magnification (scale bars, 100 μm). B) The expression of claudin-1 was measured by quantitative reverse transcription-polymerase chain reaction (qRT-PCR). *n* = 4–5 per group. C) The expression of occludin was measured by qRT-PCR. *n* = 4–5 per group. D) The expression of ZO-1 was measured by qRT-PCR. *n* = 8 per group. E) The expression of IL-1β was measured by qRT-PCR. *n* = 9–10 per group. F) The expression of IL-6 was measured by qRT-PCR. *n* = 9–10 per group. G) The expression of TNF-α was measured by qRT-PCR. *n* = 9–10 per group. All of data are the mean ± SEM and analyzed by one-way ANOVA (B–G) with Bonferroni’s post-hoc test; ZO-1: zona occludens-1, IL-1β: interleukin-1β, IL-6: interleukin-6, TNF-α: tumor necrosis factor-α. The statistical details can be found in Supplementary Table S4.

**
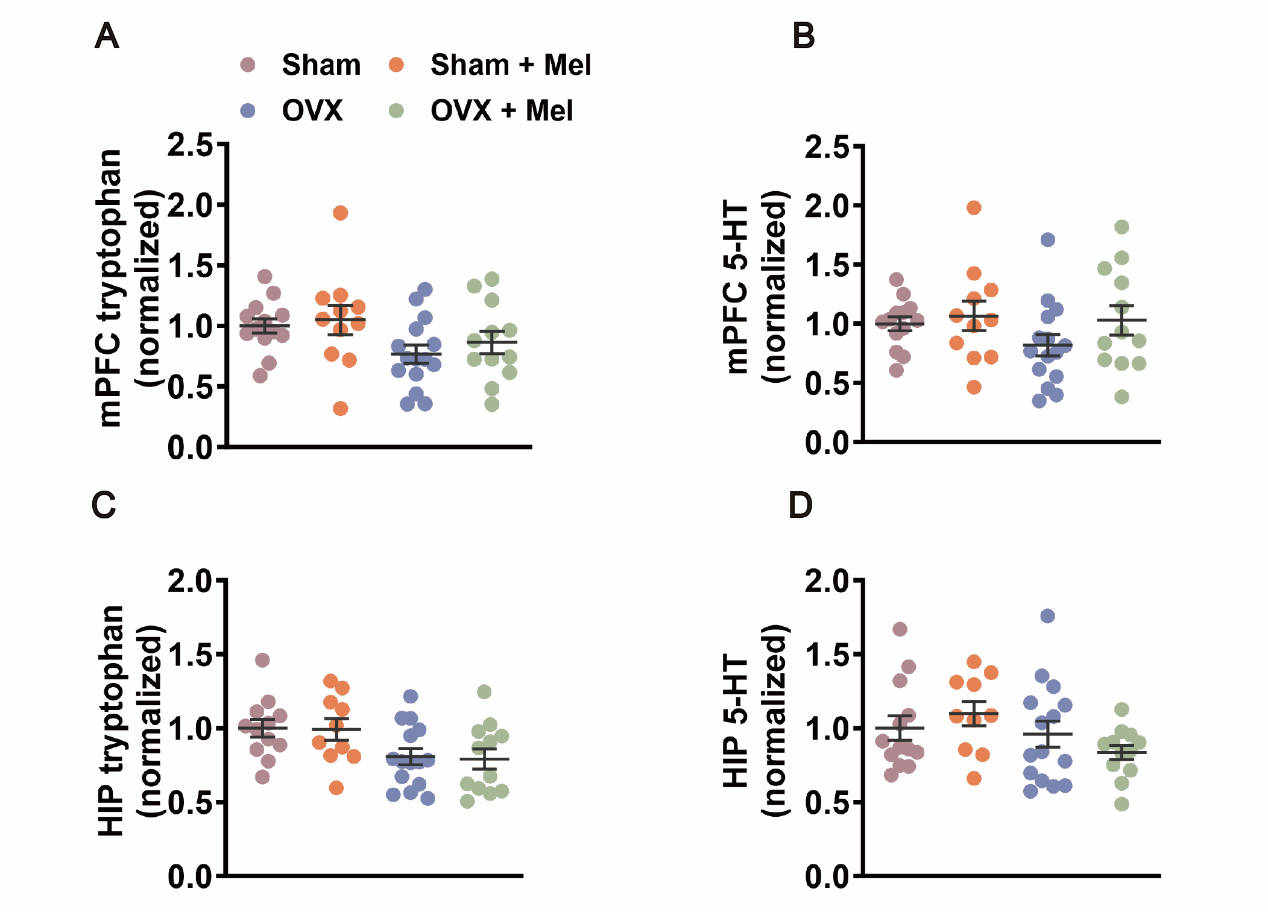
**

**Figure S3.** **OVX operation or melatonin treatment does not impact tryptophan-5-HT metabolism in the mPFC or HIP.** A) The expression of tryptophan in the mPFC was measured by ELISA of Sham mice, Sham + Mel mice, OVX mice and OVX + Mel mice. *n* = 11–15 per group. B) ELISA quantification of 5-HT protein level in the mPFC normalized to controls. *n* = 11–15 per group. C) ELISA quantification of tryptophan protein level in the HIP normalized to controls. *n* = 10–15 per group. D) ELISA quantification of 5-HT protein level in the HIP normalized to controls. *n* = 10–15 per group. All of data are the mean ± SEM and analyzed by two-way ANOVA (A–D) with Bonferroni’s post-hoc test; Sham: sham-operated mice, Sham + Mel: melatonin treated sham-operated mice, OVX: ovariectomy-operated mice, OVX + Mel: melatonin treated ovariectomy-operated mice, mPFC: medial prefrontal cortex, HIP: hippocampus. The statistical details can be found in Supplementary Table S4.

**
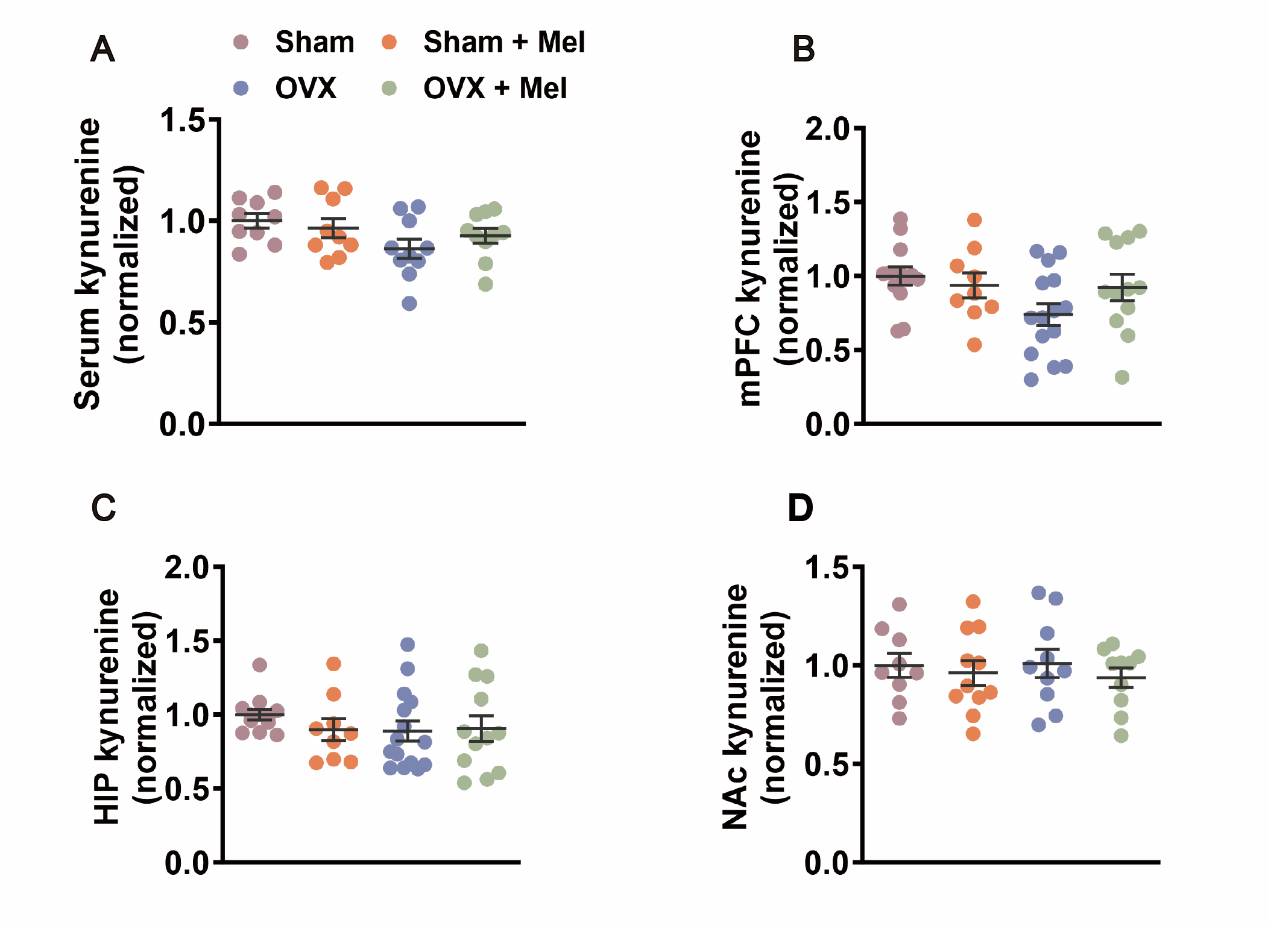
**

**Figure S4.** **OVX operation or melatonin-treated OVX operation exerts no effect on systemic tryptophan-kynurenic metabolism.** A) The expression of serum kynurenine was measured by ELISA of Sham mice, Sham + Mel mice, OVX mice and OVX + Mel mice. *n* = 9–10 per group. B) ELISA quantification of kynurenine protein level in the mPFC normalized to controls. *n* = 9–15 per group. C) ELISA quantification of kynurenine protein level in the HIP normalized to controls. *n* = 9–15 per group. D) ELISA quantification of kynurenine protein level in the NAc normalized to controls. *n* = 9–11 per group. All of data are the mean ± SEM and analyzed by two-way ANOVA (A–D) with Bonferroni’s post-hoc test; mPFC: medial prefrontal cortex, HIP: hippocampus, NAc: nucleus accumbens. The statistical details can be found in Supplementary Table S4.

**
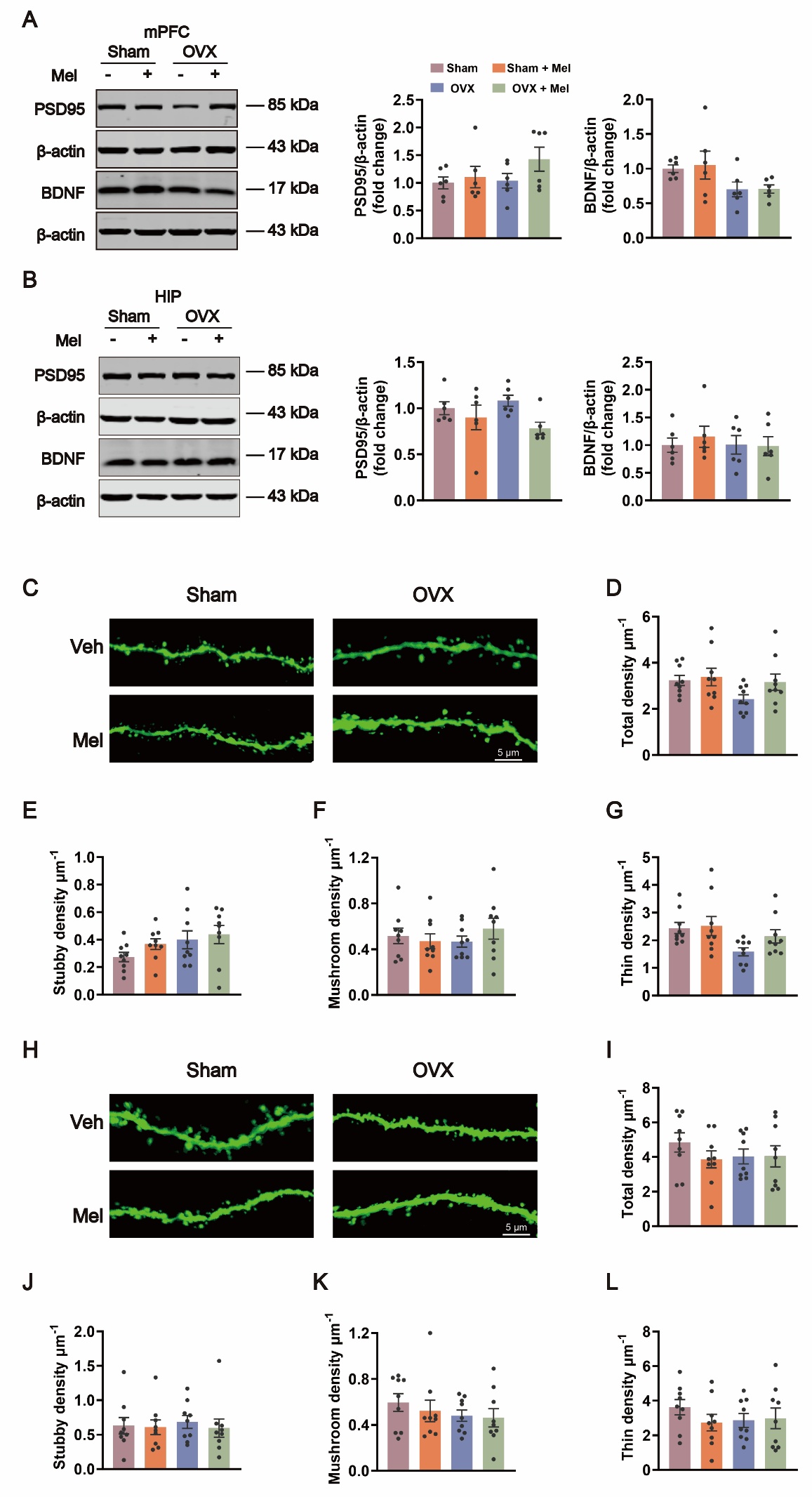
**

**Figure S5.** **Quantitative analysis of synaptic proteins and spine density following OVX operation and melatonin treatment.** A) The protein expression of PSD95, BDNF, and β-actin in the mPFC of Sham mice, Sham + Mel mice, OVX mice and OVX + Mel mice. *n* = 6 per group. B) The protein expression of PSD95, BDNF, and β-actin in HIP of Sham mice, Sham + Mel mice, OVX mice and OVX + Mel mice. *n* = 6 per group. C) Representative confocal images of spiny dendrites in the mPFC after AAV-GFP injection. D) Quantification analysis of total spine density in the mPFC. *n* = 9 per group. E) Quantification analysis of stubby spine density in the mPFC. *n* = 9 per group. F) Quantification analysis of mushroom spine density in the mPFC. *n* = 9 per group. G) Quantification analysis of thin spine density in the mPFC. *n* = 9 per group. H) Representative confocal images of spiny dendrites in the HIP after AAV-GFP injection. I) Quantification analysis of total spine density in the HIP. *n* = 9 per group. J) Quantification analysis of stubby spine density in the HIP. *n* = 9 per group. K) Quantification analysis of mushroom spine density in the HIP. *n* = 9 per group. L) Quantification analysis of thin spine density in the HIP. *n* = 9 per group. All of data are the mean ± SEM and analyzed by two-way ANOVA (A, B, D–G, I–L) with Bonferroni’s post-hoc test; mPFC: medial prefrontal cortex, HIP: hippocampus, Veh: vehicle, Mel: melatonin, PSD95: postsynaptic density protein 95, BDNF: brain-derived neurotrophic factor, AAV: adeno-associated virus, GFP: green fluorescent protein. The statistical details can be found in Supplementary Table S4.

**
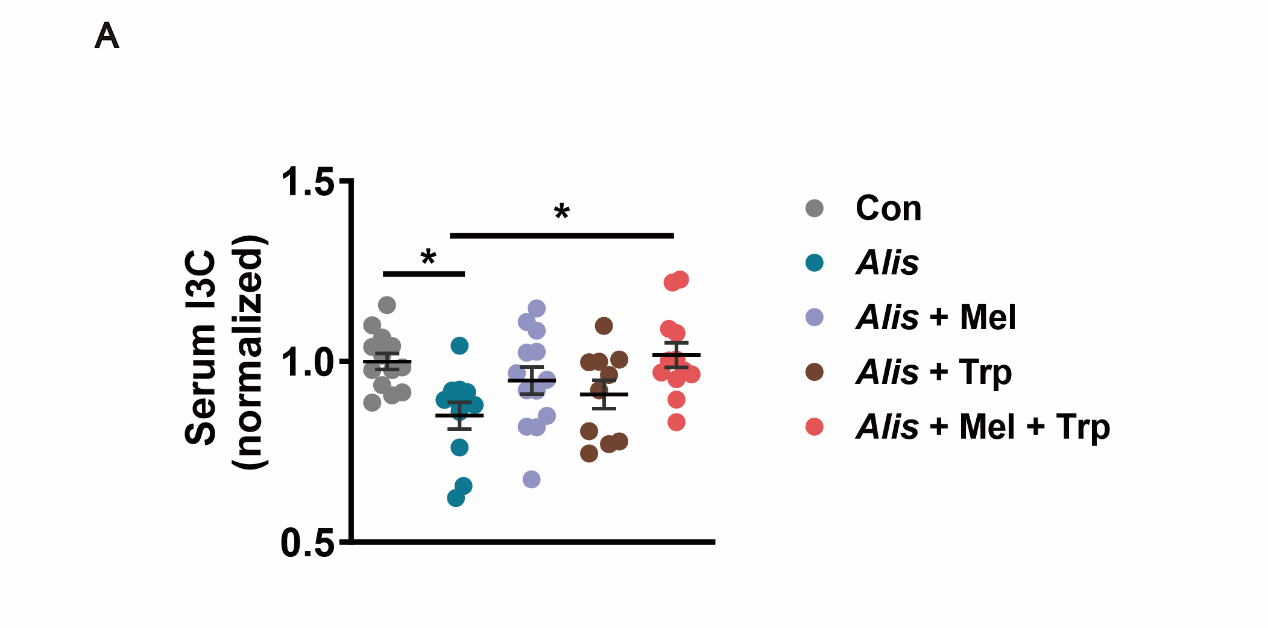
**

**Figure S6. *Alistipes Inops* colonization reduced the serum level of I3C.** A) The expression of serum I3C was measured by ELISA of Con mice, *Alis* mice, *Alis* + Mel mice, *Alis* + Trp mice and *Alis* + Mel + Trp mice. *n* = 10–13 per group. All of data are the mean ± SEM and analyzed by one-way ANOVA (A) with Bonferroni’s post-hoc test; Con: naïve female mice received sterile saline treatment, *Ali*: *Alistipes Inops*-colonized mice, *Ali* + Mel: *Alistipes Inops*-colonized mice treated with melatonin, *Ali* + Trp: *Alistipes Inops*-colonized mice treated with tryptophan, *Ali* + Mel + Trp: *Alistipes Inops*-colonized mice treated with melatonin and tryptophan, I3C: indole-3-carbinol. The statistical details can be found in Supplementary Table S4.

**Uncropped scans of key western blots**

**Figure S5A**


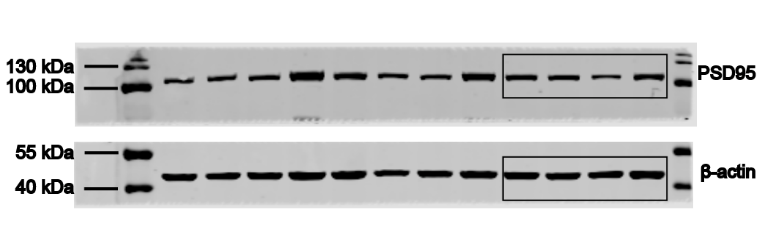

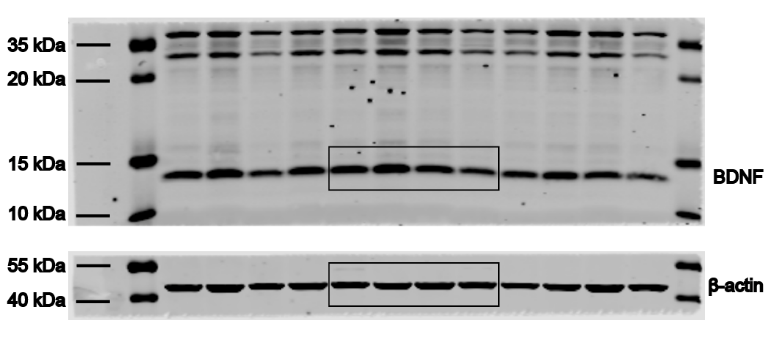


**Figure S5B**


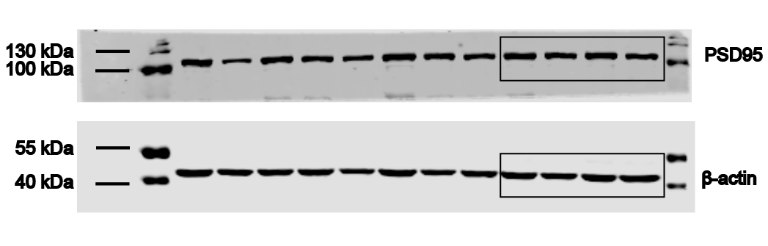


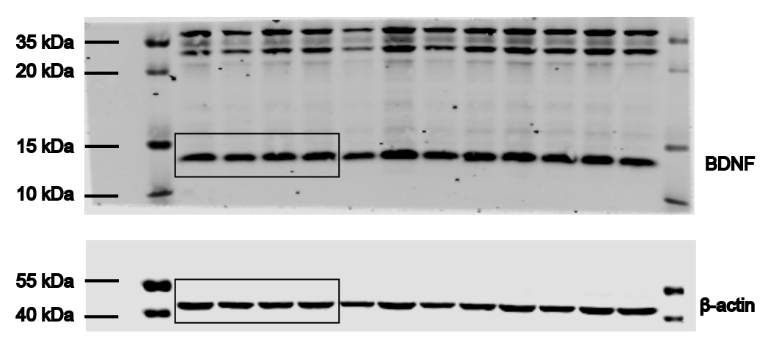


**Supplementary Table S1. All primers used.**

| **Names** | **Sequences (5' to 3')** |
| --- | --- |
| IL-1β-F: | GCAACTGTTCCTGAACTCAACT |
| IL-1β-R: | ATCTTTTGGGGTCCGTCAACT |
| IL-6-F: | ACCGCTATGAAGTTCCTCTC |
| IL-6-R: | CTCTGTGAAGTCTCCTCTCC |
| TNF-α-F: | CCCTCACACTCAGATCATCTTCT |
| TNF-α-R: | GCTACGACGTGGGCTACAG |
| occludin-F: | TGTGGGATAAGGAACACATTTATGA |
| occludin-R: | CAGACACATTTTTAACCCACTCTTCA |
| claudin-1-F: | CAACCACAATAGCGGCATC |
| claudin-1-R: | ATACAGGCTGCGGCAGATAC |
| ZO-1-F: | TGAACGCTCTCATAAGCTTCGTAA |
| ZO-1-R: | ACCGTACCAACCATCATTCATTG |
| GAPDH-F: | ATGGTGAAGGTCGGTGTG |
| GAPDH-R: | CATTCTCGGCCTTGACTG |
| *Alistipes Inops*-F: | CTACAGCATCTACTCGCTAG |
| *Alistipes Inops*-R: | GGTAATTTGCGGTACGTGTA |
| Universal bacteria-F: | ATGGYTGTCGTCAGCTCGTG |
| Universal bacteria-R: | GGGGGTTGCGCTCGTTGC |
| V3-V4 hypervariable region of 16S rDNA-encoding genes-338F: | ACTCCTACGGGAGGCAGCAG |
| V3-V4 hypervariable region of 16S rDNA-encoding genes-806R: | GGACTACHVGGGTWTCTAAT |

**Supplementary Table S2. All drugs and ELISA Kit used.**

| **Drugs** | **Source** | **Identifier** |
| --- | --- | --- |
| Melatonin | J&K Scientific | Cat# 211835 |
| Ampicillin | Meilunbio | Cat# MB1378 |
| Streptomycin | Meilunbio | Cat# MB1275 |
| Gentamicin | Meilunbio | Cat# MB1331 |
| Vancomycin | Meilunbio | Cat# MB1260 |
| Mouse tryptophan ELISA Kit | RENJIEBIO | Cat# RJ22298 |
| Mouse 5-HT ELISA Kit | RENJIEBIO | Cat# RJ16720 |
| Mouse melatonin ELISA Kit | RENJIEBIO | Cat# RJ17664 |
| Mouse estradiol ELISA Kit | RENJIEBIO | Cat# RJ17016 |
| Mouse kynurenine ELISA Kit | RENJIEBIO | Cat# RJ17525 |
| Mouse indole-3-carbinol ELISA Kit | RENJIEBIO | Cat# RJ27567 |

**Supplementary Table S3. All antibodies used.**

| **Antibodies** | **Source** | **Identifier** |
| --- | --- | --- |
| Anti-β-actin | Santa Cruz | sc-47778 |
| Anti-PSD95 | Abcam | ab18285 |
| Anti-BDNF | Abcam | ab108319 |
| 800CW Goat anti-Rabbit IgG | LI-COR | 926-32211 |
| 800CW Goat anti-Mouse IgG | LI-COR | 926-32210 |

**Supplementary Table S4. Statistical analysis for Figures 1-7 and Supplementary Figures S1-6.**

| **Figure and numbers of animals used** | **Statistical analysis** | | ***Post hoc tests*** | **Mean ± SEM.** |
| --- | --- | --- | --- | --- |
| **1B**: Body weight was monitored  Sham  (n = 10)  Sham + Mel  (n = 11)  OVX  (n = 10)  OVX + Mel  (n = 10) | Three-way ANOVA  Time:  F (9, 370) = 168.6,  p < 0.0001  OVX:  F (1, 370) = 286.9,  p < 0.0001  Drug:  F (1, 370) = 6.483,  p = 0.0113  Time × OVX:  F (9, 370) = 27.53,  p < 0.0001  Time × Drug:  F (9, 370) = 0.7493,  p = 0.6636  OVX × Drug:  F (1, 370) = 0.08447,  p = 0.7715  Time × OVX × Drug:  F (9, 370) = 0.1371,  p = 0.9986 | | Bonferroni’s multiple comparisons test  Row 1: Sham vs. Row 1: Sham + Mel,  p > 0.9999  Row 1: Sham vs. Row 1: OVX,  p > 0.9999  Row 1: Sham vs. Row 1: OVX + Mel,  p > 0.9999  Row 1: Sham + Mel vs. Row 1: OVX,  p > 0.9999  Row 1: Sham + Mel vs. Row 1: OVX + Mel,  p > 0.9999  Row 1: OVX vs. Row 1: OVX + Mel,  p > 0.9999  Row 2: Sham vs. Row 2: Sham + Mel,  p > 0.9999  Row 2: Sham vs. Row 2: OVX,  p > 0.9999  Row 2: Sham vs. Row 2: OVX + Mel,  p > 0.9999  Row 2: Sham + Mel vs. Row 2: OVX,  p > 0.9999  Row 2: Sham + Mel vs. Row 2: OVX + Mel,  p > 0.9999  Row 2: OVX vs. Row 2: OVX + Mel,  p > 0.9999  Row 3: Sham vs. Row 3: Sham + Mel,  p > 0.9999  Row 3: Sham vs. Row 3: OVX,  p > 0.9999  Row 3: Sham vs. Row 3: OVX + Mel,  p > 0.9999  Row 3: Sham + Mel vs. Row 3: OVX,  p > 0.9999  Row 3: Sham + Mel vs. Row 3: OVX + Mel,  p > 0.9999  Row 3: OVX vs. Row 3: OVX + Mel,  p > 0.9999  Row 4: Sham vs. Row 4: Sham + Mel,  p > 0.9999  Row 4: Sham vs. Row 4: OVX,  p > 0.9999  Row 4: Sham vs. Row 4: OVX + Mel,  p > 0.9999  Row 4: Sham + Mel vs. Row 4: OVX,  p > 0.9999  Row 4: Sham + Mel vs. Row 4: OVX + Mel,  p > 0.9999  Row 4: OVX vs. Row 4: OVX + Mel,  p > 0.9999  Row 5: Sham vs. Row 5: Sham + Mel,  p > 0.9999  Row 5: Sham vs. Row 5: OVX,  p = 0.0081  Row 5: Sham vs. Row 5: OVX + Mel,  p = 0.6161  Row 5: Sham + Mel vs. Row 5: OVX,  p < 0.0001  Row 5: Sham + Mel vs. Row 5: OVX + Mel,  p = 0.0033  Row 5: OVX vs. Row 5: OVX + Mel,  p > 0.9999  Row 6: Sham vs. Row 6: Sham + Mel,  p > 0.9999  Row 6: Sham vs. Row 6: OVX,  p < 0.0001  Row 6: Sham vs. Row 6: OVX + Mel,  p < 0.0001  Row 6: Sham + Mel vs. Row 6: OVX,  p < 0.0001  Row 6: Sham + Mel vs. Row 6: OVX + Mel,  p < 0.0001  Row 6: OVX vs. Row 6: OVX + Mel,  p > 0.9999  Row 7: Sham vs. Row 7: Sham + Mel,  p > 0.9999  Row 7: Sham vs. Row 7: OVX,  p < 0.0001  Row 7: Sham vs. Row 7: OVX + Mel,  p < 0.0001  Row 7: Sham + Mel vs. Row 7: OVX,  p < 0.0001  Row 7: Sham + Mel vs. Row 7: OVX + Mel,  p < 0.0001  Row 7: OVX vs. Row 7: OVX + Mel,  p > 0.9999  Row 8: Sham vs. Row 8: Sham + Mel,  p > 0.9999  Row 8: Sham vs. Row 8: OVX,  p < 0.0001  Row 8: Sham vs. Row 8: OVX + Mel,  p < 0.0001  Row 8: Sham + Mel vs. Row 8: OVX,  p < 0.0001  Row 8: Sham + Mel vs. Row 8: OVX + Mel,  p < 0.0001  Row 8: OVX vs. Row 8: OVX + Mel,  p > 0.9999  Row 9: Sham vs. Row 9: Sham + Mel,  p > 0.9999  Row 9: Sham vs. Row 9: OVX,  p = 0.0059  Row 9: Sham vs. Row 9: OVX + Mel,  p = 0.0152  Row 9: Sham + Mel vs. Row 9: OVX,  p = 0.0046  Row 9: Sham + Mel vs. Row 9: OVX + Mel,  p = 0.0121  Row 9: OVX vs. Row 9: OVX + Mel,  p > 0.9999  Row 10: Sham vs. Row 10: Sham + Mel,  p > 0.9999  Row 10: Sham vs. Row 10: OVX,  p = 0.0179  Row 10: Sham vs. Row 10: OVX + Mel,  p = 0.1219  Row 10: Sham + Mel vs. Row 10: OVX,  p = 0.0172  Row 10: Sham + Mel vs. Row 10: OVX + Mel,  p = 0.1227  Row 10: OVX vs. Row 10: OVX + Mel,  p > 0.9999 | Mean ± s.e.m.  Row 1:  Sham  15.69 ± 0.26  Sham + Mel  15.76 ± 0.14  OVX  15.76 ± 0.24  OVX + Mel  15.76 ± 0.13    Row 2:  Sham  18.18 ± 0.42  Sham + Mel  18.30 ± 0.25  OVX  17.89 ± 0.19  OVX + Mel  18.04 ± 0.16    Row 3:  Sham  18.72 ± 0.25  Sham + Mel  18.52 ± 0.28  OVX  18.51 ± 0.15  OVX + Mel  18.37 ± 0.13    Row 4:  Sham  19.02 ± 0.25  Sham + Mel  18.66 ± 0.24  OVX  19.43 ± 0.20  OVX + Mel  19.13 ± 0.12    Row 5:  Sham  19.00 ± 0.21  Sham + Mel  18.60 ± 0.22  OVX  20.51 ± 0.26  OVX + Mel  20.14 ± 0.15    Row 6:  Sham  19.39 ± 0.19  Sham + Mel  18.83 ± 0.24  OVX  21.59 ± 0.20  OVX + Mel  21.28 ± 0.18    Row 7:  Sham  19.26 ± 0.22  Sham + Mel  18.62 ± 0.21  OVX  22.27 ± 0.27  OVX + Mel  22.05 ± 0.26    Row 8:  Sham  19.44 ± 0.27  Sham + Mel  19.24 ± 0.21  OVX  22.16 ± 0.26  OVX + Mel  21.90 ± 0.23    Row 9:  Sham  19.49 ± 0.23  Sham + Mel  19.50 ± 0.22  OVX  21.02 ± 0.29  OVX + Mel  20.95 ± 0.26    Row 10:  Sham  19.59 ± 0.27  Sham + Mel  19.62 ± 0.19  OVX  21.03 ± 0.37  OVX + Mel  20.88 ± 0.29 |
| **1C**: Body weight on day 28  Sham  (n = 10)  Sham + Mel  (n = 11)  OVX  (n = 10)  OVX + Mel  (n = 10) | Two-way ANOVA  Interaction:  F (1, 37) = 0.1090,  p = 0.7431  Drug: F (1, 37) = 0.05030,  p = 0.8238  OVX: F (1, 37) = 22.51,  p < 0.0001 | | Bonferroni’s multiple comparisons test  Vehicle : Sham vs. Vehicle : OVX,  p = 0.0065  Vehicle : Sham vs. Melatonin : Sham,  p > 0.9999  Vehicle : Sham vs. Melatonin : OVX,  p = 0.0189  Vehicle : OVX vs. Melatonin : Sham,  p = 0.0063  Vehicle : OVX vs. Melatonin : OVX,  p > 0.9999  Melatonin : Sham vs. Melatonin : OVX,  p = 0.0189 | Mean ± s.e.m.  Sham  19.59 ± 0.27  Sham + Mel  19.62 ± 0.19  OVX  21.03 ± 0.37  OVX + Mel  20.88 ± 0.29 |
| **1D**: Weight gain  Sham  (n = 10)  Sham + Mel  (n = 11)  OVX  (n = 10)  OVX + Mel  (n = 10) | Two-way ANOVA  Interaction:  F (1, 37) = 0.06610,  p = 0.7985  Drug:  F (1, 37) = 0.1639,  p = 0.6879  OVX: F (1, 37) = 30.20,  p < 0.0001 | | Bonferroni’s multiple comparisons test  Vehicle : Sham vs. Vehicle : OVX,  p = 0.0016  Vehicle : Sham vs. Melatonin : Sham,  p > 0.9999  Vehicle : Sham vs. Melatonin : OVX,  p = 0.0063  Vehicle : OVX vs. Melatonin : Sham,  p = 0.0009  Vehicle : OVX vs. Melatonin : OVX,  p > 0.9999  Melatonin : Sham vs. Melatonin : OVX,  p = 0.0036 | Mean ± s.e.m.  Sham  3.90 ± 0.23  Sham + Mel  3.86 ± 0.19  OVX  5.27 ± 0.27  OVX + Mel  5.11 ± 0.26 |
| **1E**: Behavioral test of SPT  Sham  (n = 10)  Sham + Mel  (n = 11)  OVX  (n = 10)  OVX + Mel  (n = 10) | Two-way ANOVA  Interaction:  F (1, 37) = 167.6,  p < 0.0001  Drug:  F (1, 37) = 150.1,  p < 0.0001  OVX:  F (1, 37) = 120.9,  p < 0.0001 | | Bonferroni’s multiple comparisons test  Vehicle : Sham vs. Vehicle : OVX,  p < 0.0001  Vehicle : Sham vs. Melatonin : Sham,  p > 0.9999  Vehicle : Sham vs. Melatonin : OVX,  p > 0.9999  Vehicle : OVX vs. Melatonin : Sham,  p < 0.0001  Vehicle : OVX vs. Melatonin : OVX,  p < 0.0001  Melatonin : Sham vs. Melatonin : OVX,  p > 0.9999 | Mean ± s.e.m.  Sham  78.24 ± 1.23  Sham + Mel  76.89 ± 1.27  OVX  31.59 ± 2.16  OVX + Mel  80.69 ± 2.80 |
| **1F**: Behavioral test of TST  Sham  (n = 10)  Sham + Mel  (n = 11)  OVX  (n = 10)  OVX + Mel  (n = 10) | Two-way ANOVA  Interaction:  F (1, 37) = 7.307,  p = 0.0103  Drug:  F (1, 37) = 4.587,  p = 0.0389  OVX:  F (1, 37) = 2.329,  p = 0.1355 | | Bonferroni’s multiple comparisons test  Vehicle : Sham vs. Vehicle : OVX,  p = 0.0324  Vehicle : Sham vs. Melatonin : Sham,  p > 0.9999  Vehicle : Sham vs. Melatonin : OVX,  p > 0.9999  Vehicle : OVX vs. Melatonin : Sham,  p = 0.0753  Vehicle : OVX vs. Melatonin : OVX,  p = 0.0101  Melatonin : Sham vs. Melatonin : OVX,  p > 0.9999 | Mean ± s.e.m.  Sham  128.4 ± 13.3  Sham + Mel  135.7 ± 10.5  OVX  183.5 ± 12.5  OVX + Mel  120.3 ± 15.7 |
| **1G**: Behavioral test of FST  Sham  (n = 10)  Sham + Mel  (n = 11)  OVX  (n = 10)  OVX + Mel  (n = 10) | Two-way ANOVA  Interaction:  F (1, 37) = 11.90,  p = 0.0014  Drug:  F (1, 37) = 4.195,  p = 0.0477  OVX:  F (1, 37) = 12.34,  p = 0.0012 | | Bonferroni’s multiple comparisons test  Vehicle : Sham vs. Vehicle : OVX,  p < 0.0001  Vehicle : Sham vs. Melatonin : Sham,  p > 0.9999  Vehicle : Sham vs. Melatonin : OVX,  p > 0.9999  Vehicle : OVX vs. Melatonin : Sham,  p = 0.0019  Vehicle : OVX vs. Melatonin : OVX,  p = 0.0028  Melatonin : Sham vs. Melatonin : OVX,  p > 0.9999 | Mean ± s.e.m.  Sham  93.4 ± 7.8  Sham + Mel  106.0 ± 9.5  OVX  156.0 ± 9.1  OVX + Mel  106.6 ± 9.3 |
| **1H**: Behavioral test of OFT  Sham  (n = 10)  Sham + Mel  (n = 11)  OVX  (n = 10)  OVX + Mel  (n = 10) | Two-way ANOVA  Interaction:  F (1, 37) = 0.8526,  p = 0.3618  Drug:  F (1, 37) = 0.007724,  p = 0.9304  OVX:  F (1, 37) = 0.009546,  p = 0.9227 | | Bonferroni’s multiple comparisons test  Vehicle : Sham vs. Vehicle : OVX,  p > 0.9999  Vehicle : Sham vs. Melatonin : Sham,  p > 0.9999  Vehicle : Sham vs. Melatonin : OVX,  p > 0.9999  Vehicle : OVX vs. Melatonin : Sham,  p > 0.9999  Vehicle : OVX vs. Melatonin : OVX,  p > 0.9999  Melatonin : Sham vs. Melatonin : OVX,  p > 0.9999 | Mean ± s.e.m.  Sham  38.677 ± 2.051  Sham + Mel  40.489 ± 2.413  OVX  40.891 ± 1.838  OVX + Mel  38.698 ± 2.254 |
| **1I**: Behavioral test of OFT  Sham  (n = 10)  Sham + Mel  (n = 11)  OVX  (n = 10)  OVX + Mel  (n = 10) | Two-way ANOVA  Interaction:  F (1, 37) = 0.0008804,  p = 0.9765  Drug:  F (1, 37) = 0.002103,  p = 0.9637  OVX:  F (1, 37) = 0.5034,  p = 0.4825 | | Bonferroni’s multiple comparisons test  Vehicle : Sham vs. Vehicle : OVX,  p > 0.9999  Vehicle : Sham vs. Melatonin : Sham,  p > 0.9999  Vehicle : Sham vs. Melatonin : OVX,  p > 0.9999  Vehicle : OVX vs. Melatonin : Sham,  p > 0.9999  Vehicle : OVX vs. Melatonin : OVX,  p > 0.9999  Melatonin : Sham vs. Melatonin : OVX,  p > 0.9999 | Mean ± s.e.m.  Sham  5.466 ± 0.586  Sham + Mel  5.504 ± 0.444  OVX  5.122 ± 0.285  OVX + Mel  5.130 ± 0.643 |
| **1J**: Serum estradiol level  Sham  (n = 7)  Sham + Mel  (n = 5)  OVX  (n = 8)  OVX + Mel  (n = 7) | Two-way ANOVA  Interaction:  F (1, 23) = 11.39,  p = 0.0026  Drug:  F (1, 23) = 0.5493,  p = 0.4661  OVX:  F (1, 23) = 124.2,  p < 0.0001 | | Bonferroni’s multiple comparisons test  Vehicle : Sham vs. Vehicle : OVX,  p < 0.0001  Vehicle : Sham vs. Melatonin : Sham,  p = 0.0689  Vehicle : Sham vs. Melatonin : OVX,  p < 0.0001  Vehicle : OVX vs. Melatonin : Sham,  p < 0.0001  Vehicle : OVX vs. Melatonin : OVX,  p = 0.3527  Melatonin : Sham vs. Melatonin : OVX,  p = 0.0002 | Mean ± s.e.m.  Sham  1.0000 ± 0.0513  Sham + Mel  0.8286 ± 0.0395  OVX  0.3956 ± 0.0320  OVX + Mel  0.5052 ± 0.0385 |
| **1K**: colonic melatonin level  Sham  (n = 10)  Sham + Mel  (n = 7)  OVX  (n = 7)  OVX + Mel  (n = 8) | Two-way ANOVA  Interaction:  F (1, 28) = 1.666,  p = 0.2073  Drug:  F (1, 28) = 13.95,  p = 0.0009  OVX:  F (1, 28) = 54.43,  p < 0.0001 | | Bonferroni’s multiple comparisons test  Vehicle : Sham vs. Vehicle : OVX,  p < 0.0001  Vehicle : Sham vs. Melatonin : Sham,  p = 0.5234  Vehicle : Sham vs. Melatonin : OVX,  p = 0.0628  Vehicle : OVX vs. Melatonin : Sham,  p < 0.0001  Vehicle : OVX vs. Melatonin : OVX,  p = 0.0102  Melatonin : Sham vs. Melatonin : OVX,  p = 0.0015 | Mean ± s.e.m.  Sham  1.0000 ± 0.0421  Sham + Mel  1.1037 ± 0.0434  OVX  0.6322 ± 0.0346  OVX + Mel  0.8454 ± 0.0435 |
| **1L**: Serum melatonin level  Sham  (n = 12)  Sham + Mel  (n = 7)  OVX  (n = 7)  OVX + Mel  (n = 7) | Two-way ANOVA  Interaction:  F (1, 29) = 0.7721,  p = 0.3868  Drug:  F (1, 29) = 0.003771,  p = 0.9515  OVX:  F (1, 29) = 3.183,  p = 0.0849 | | Bonferroni’s multiple comparisons test  Vehicle : Sham vs. Vehicle : OVX,  p > 0.9999  Vehicle : Sham vs. Melatonin : Sham,  p > 0.9999  Vehicle : Sham vs. Melatonin : OVX,  p > 0.9999  Vehicle : OVX vs. Melatonin : Sham,  p > 0.9999  Vehicle : OVX vs. Melatonin : OVX,  p > 0.9999  Melatonin : Sham vs. Melatonin : OVX,  p = 0.5113 | Mean ± s.e.m.  Sham  1.0000 ± 0.0419  Sham + Mel  0.9439 ± 0.0287  OVX  1.0540 ± 0.0731  OVX + Mel  1.1027 ± 0.0865 |
| **2B**: Fecal DNA concentration  Sham  (n = 4)  Sham + ABX  (n = 3)  Sham + ABX + Mel  (n = 3)  OVX + ABX  (n = 3)  OVX + ABX + Mel  (n = 3) | One-way ANOVA  Treatment:  F (4, 11) = 145.9,  p < 0.0001 | | Bonferroni’s multiple comparisons test  Sham vs. Sham + ABX,  p < 0.0001  Sham vs. Sham + ABX + Mel,  p < 0.0001  Sham vs. OVX + ABX,  p < 0.0001  Sham vs. OVX + ABX + Mel,  p < 0.0001  Sham + ABX vs. Sham + ABX +Mel,  p > 0.9999  Sham + ABX vs. OVX + ABX,  p > 0.9999  Sham + ABX vs. OVX + ABX + Mel,  p > 0.9999  Sham + ABX +Mel vs. OVX + ABX,  p > 0.9999  Sham + ABX +Mel vs. OVX + ABX + Mel,  p > 0.9999  OVX + ABX vs. OVX + ABX + Mel,  p > 0.9999 | Mean ± s.e.m.  Sham  203.8 ± 12.5  Sham + ABX  13.5 ± 0.8  Sham + ABX + Mel  15.7 ± 2.0  OVX + ABX  14.3 ± 3.4  OVX + ABX + Mel  12.8 ± 3.0 |
| **2C**: Ct value  Sham  (n = 4)  Sham + ABX  (n = 3)  Sham + ABX + Mel  (n = 3)  OVX + ABX  (n = 3)  OVX + ABX + Mel  (n = 3) | One-way ANOVA  Treatment:  F (4, 11) = 68.84,  p < 0.0001 | | Bonferroni’s multiple comparisons test  Sham vs. Sham + ABX,  p < 0.0001  Sham vs. Sham + ABX +Mel,  p < 0.0001  Sham vs. OVX + ABX,  p < 0.0001  Sham vs. OVX + ABX + Mel,  p < 0.0001  Sham + ABX vs. Sham + ABX +Mel,  p > 0.9999  Sham + ABX vs. OVX + ABX,  p > 0.9999  Sham + ABX vs. OVX + ABX + Mel,  p > 0.9999  Sham + ABX +Mel vs. OVX + ABX,  p > 0.9999  Sham + ABX +Mel vs. OVX + ABX + Mel,  p > 0.9999  OVX + ABX vs. OVX + ABX + Mel,  p > 0.9999 | Mean ± s.e.m.  Sham  11.37 ± 0.32  Sham + ABX  15.11 ± 0.04  Sham + ABX + Mel  15.21 ± 0.20  OVX + ABX  15.10 ± 0.07  OVX + ABX + Mel  14.87 ± 0.14 |
| 2D: Body weight  Sham + ABX  (n = 10)  Sham + ABX + Mel  (n = 10)  OVX + ABX  (n = 13)  OVX + ABX + Mel  (n = 14) | Three-way ANOVA  Time:  F (9, 429) = 164.4,  p < 0.0001  OVX:  F (1, 429) = 135.8,  p < 0.0001  Drug:  F (1, 429) = 19.19,  p < 0.0001  Time × OVX:  F (9, 429) = 45.03,  p < 0.0001  Time × Drug:  F (9, 429) = 2.174,  p = 0.0228  OVX × Drug:  F (1, 429) = 1.448,  p = 0.2295  Time × OVX × Drug:  F (9, 429) = 1.508,  p = 0.1424 | | Bonferroni’s multiple comparisons test  Row 1: Sham + ABX vs. Row 1: Sham + ABX +Mel,  p > 0.9999  Row 1: Sham + ABX vs. Row 1: OVX + ABX,  p = 0.3513  Row 1: Sham + ABX vs. Row 1: OVX + ABX + Mel,  p > 0.9999  Row 1: Sham + ABX +Mel vs. Row 1: OVX + ABX,  p = 0.2168  Row 1: Sham + ABX +Mel vs. Row 1: OVX + ABX + Mel,  p > 0.9999  Row 1: OVX + ABX vs. Row 1: OVX + ABX + Mel,  p > 0.9999  Row 2: Sham + ABX vs. Row 2: Sham + ABX +Mel,  p > 0.9999  Row 2: Sham + ABX vs. Row 2: OVX + ABX,  p > 0.9999  Row 2: Sham + ABX vs. Row 2: OVX + ABX + Mel,  p = 0.0002  Row 2: Sham + ABX +Mel vs. Row 2: OVX + ABX,  p > 0.9999  Row 2: Sham + ABX +Mel vs. Row 2: OVX + ABX + Mel,  p = 0.0002  Row 2: OVX + ABX vs. Row 2: OVX + ABX + Mel,  p > 0.9999  Row 3: Sham + ABX vs. Row 3: Sham + ABX +Mel,  p > 0.9999  Row 3: Sham + ABX vs. Row 3: OVX + ABX,  p = 0.3826  Row 3: Sham + ABX vs. Row 3: OVX + ABX + Mel,  p = 0.0127  Row 3: Sham + ABX +Mel vs. Row 3: OVX + ABX,  p > 0.9999  Row 3: Sham + ABX +Mel vs. Row 3: OVX + ABX + Mel,  p > 0.9999  Row 3: OVX + ABX vs. Row 3: OVX + ABX + Mel,  p > 0.9999  Row 4: Sham + ABX vs. Row 4: Sham + ABX +Mel,  p > 0.9999  Row 4: Sham + ABX vs. Row 4: OVX + ABX,  p > 0.9999  Row 4: Sham + ABX vs. Row 4: OVX + ABX + Mel,  p > 0.9999  Row 4: Sham + ABX +Mel vs. Row 4: OVX + ABX,  p > 0.9999  Row 4: Sham + ABX +Mel vs. Row 4: OVX + ABX + Mel,  p > 0.9999  Row 4: OVX + ABX vs. Row 4: OVX + ABX + Mel,  p > 0.9999  Row 5: Sham + ABX vs. Row 5: Sham + ABX +Mel,  p > 0.9999  Row 5: Sham + ABX vs. Row 5: OVX + ABX,  p = 0.2863  Row 5: Sham + ABX vs. Row 5: OVX + ABX + Mel,  p > 0.9999  Row 5: Sham + ABX +Mel vs. Row 5: OVX + ABX,  p = 0.0026  Row 5: Sham + ABX +Mel vs. Row 5: OVX + ABX + Mel,  p > 0.9999  Row 5: OVX + ABX vs. Row 5: OVX + ABX + Mel,  p > 0.9999  Row 6: Sham + ABX vs. Row 6: Sham + ABX +Mel,  p > 0.9999  Row 6: Sham + ABX vs. Row 6: OVX + ABX,  p < 0.0001  Row 6: Sham + ABX vs. Row 6: OVX + ABX + Mel,  p < 0.0001  Row 6: Sham + ABX +Mel vs. Row 6: OVX + ABX,  p < 0.0001  Row 6: Sham + ABX +Mel vs. Row 6: OVX + ABX + Mel,  p < 0.0001  Row 6: OVX + ABX vs. Row 6: OVX + ABX + Mel,  p > 0.9999  Row 7: Sham + ABX vs. Row 7: Sham + ABX +Mel,  p > 0.9999  Row 7: Sham + ABX vs. Row 7: OVX + ABX,  p < 0.0001  Row 7: Sham + ABX vs. Row 7: OVX + ABX + Mel,  p = 0.0020  Row 7: Sham + ABX +Mel vs. Row 7: OVX + ABX,  p < 0.0001  Row 7: Sham + ABX +Mel vs. Row 7: OVX + ABX + Mel,  p = 0.0002  Row 7: OVX + ABX vs. Row 7: OVX + ABX + Mel,  p = 0.0350  Row 8: Sham + ABX vs. Row 8: Sham + ABX +Mel,  p > 0.9999  Row 8: Sham + ABX vs. Row 8: OVX + ABX,  p < 0.0001  Row 8: Sham + ABX vs. Row 8: OVX + ABX + Mel,  p < 0.0001  Row 8: Sham + ABX +Mel vs. Row 8: OVX + ABX,  p < 0.0001  Row 8: Sham + ABX +Mel vs. Row 8: OVX + ABX + Mel,  p < 0.0001  Row 8: OVX + ABX vs. Row 8: OVX + ABX + Mel,  p > 0.9999  Row 9: Sham + ABX vs. Row 9: Sham + ABX +Mel,  p > 0.9999  Row 9: Sham + ABX vs. Row 9: OVX + ABX,  p < 0.0001  Row 9: Sham + ABX vs. Row 9: OVX + ABX + Mel,  p < 0.0001  Row 9: Sham + ABX +Mel vs. Row 9: OVX + ABX,  p < 0.0001  Row 9: Sham + ABX +Mel vs. Row 9: OVX + ABX + Mel,  p < 0.0001  Row 9: OVX + ABX vs. Row 9: OVX + ABX + Mel,  p > 0.9999  Row 10: Sham + ABX vs. Row 10: Sham + ABX +Mel,  p > 0.9999  Row 10: Sham + ABX vs. Row 10: OVX + ABX,  p = 0.0687  Row 10: Sham + ABX vs. Row 10: OVX + ABX + Mel,  p = 0.2063  Row 10: Sham + ABX +Mel vs. Row 10: OVX + ABX,  p = 0.0001  Row 10: Sham + ABX +Mel vs. Row 10: OVX + ABX + Mel,  p = 0.0004  Row 10: OVX + ABX vs. Row 10: OVX + ABX + Mel,  p > 0.9999 | Mean ± s.e.m.  Row 1:  Sham + ABX  17.16 ± 0.12  Sham + ABX + Me  17.20 ± 0.23  OVX + ABX  16.12 ± 0.17  OVX + ABX + Mel  16.77 ± 0.31    Row 2:  Sham + ABX  17.88 ± 0.14  Sham + ABX + Me  17.87 ± 0.13  OVX + ABX  17.17 ± 0.25  OVX + ABX + Mel  16.35 ± 0.15    Row 3:  Sham + ABX  18.58 ± 0.22  Sham + ABX + Mel  18.12 ± 0.15  OVX + ABX  17.54 ± 0.19  OVX + ABX + Mel  17.32 ± 0.13    Row 4:  Sham + ABX  18.62 ± 0.19  Sham + ABX + Mel  18.20 ± 0.17  OVX + ABX  18.33 ± 0.20  OVX + ABX + Mel  17.93 ± 0.15    Row 5:  Sham + ABX  18.73 ± 0.24  Sham + ABX + Mel  18.41 ± 0.19  OVX + ABX  19.79 ± 0.21  OVX + ABX + Mel  19.14 ± 0.16    Row 6:  Sham + ABX  18.79 ± 0.20  Sham + ABX + Me  18.54 ± 0.21  OVX + ABX  21.07 ± 0.25  OVX + ABX + Mel  20.57 ± 0.22    Row 7:  Sham + ABX  19.11 ± 0.21  Sham + ABX + Me  18.98 ± 0.24  OVX + ABX  21.59 ± 0.17  OVX + ABX + Mel  20.49 ± 0.24    Row 8:  Sham + ABX  19.29 ± 0.24  Sham + ABX + Mel  19.06 ± 0.23  OVX + ABX  21.66 ± 0.19  OVX + ABX + Mel  20.96 ± 0.20    Row 9:  Sham + ABX  19.11 ± 0.26  Sham + ABX + Me  19.23 ± 0.24  OVX + ABX  21.21 ± 0.19  OVX + ABX + Mel  21.43 ± 0.17    Row 10:  Sham + ABX  19.84 ± 0.24  Sham + ABX + Mel  19.43 ± 0.18  OVX + ABX  21.00 ± 0.19  OVX + ABX + Mel  20.90 ± 0.17 |
| **2E**: Weight on the 28th day  Sham  (n = 25)  Sham + Mel  (n = 9)  OVX  (n = 15)  OVX + Mel  (n = 14)  Sham + ABX  (n = 10)  Sham + Mel + ABX  (n = 10)  OVX + ABX  (n = 13)  OVX + Mel + ABX  (n = 14) | Three-way ANOVA  ABX：  F (1, 102) = 0.3525,  p = 0.5540  OVX：  F (1, 102) = 124.7,  p < 0.0001  Drug：  F (1, 102) = 0.4233,  p = 0.5168  ABX × OVX：  F (1, 102) = 0.2416,  p = 0.6241  ABX × Drug：  F (1, 102) = 2.000,  p = 0.1603  OVX × Drug：  F (1, 102) = 0.2196,  p = 0.6403  ABX × OVX × Drug：  F (1, 102) = 0.6273,  p = 0.4302 | | Bonferroni’s multiple comparisons test  Water : Sham vs. Water : Sham + Mel,  p > 0.9999  Water : Sham vs. Water : OVX,  p < 0.0001  Water : Sham vs. Water : OVX + Mel,  p < 0.0001  Water : Sham vs. ABX : Sham,  p > 0.9999  Water : Sham vs. ABX : Sham + Mel,  p > 0.9999  Water : Sham vs. ABX : OVX,  p < 0.0001  Water : Sham vs. ABX : OVX + Mel,  p < 0.0001  Water : Sham + Mel vs. Water : OVX,  p < 0.0001  Water : Sham + Mel vs. Water : OVX + Mel,  p < 0.0001  Water : Sham + Mel vs. ABX : Sham,  p > 0.9999  Water : Sham + Mel vs. ABX : Sham + Mel,  p > 0.9999  Water : Sham + Mel vs. ABX : OVX,  p < 0.0001  Water : Sham + Mel vs. ABX : OVX + Mel,  p < 0.0001  Water : OVX vs. Water : OVX + Mel,  p > 0.9999  Water : OVX vs. ABX : Sham,  p = 0.0013  Water : OVX vs. ABX : Sham + Mel,  p < 0.0001  Water : OVX vs. ABX : OVX,  p > 0.9999  Water : OVX vs. ABX : OVX + Mel,  p > 0.9999  Water : OVX + Mel vs. ABX : Sham,  p = 0.0007  Water : OVX + Mel vs. ABX : Sham + Mel,  p < 0.0001  Water : OVX + Mel vs. ABX : OVX,  p > 0.9999  Water : OVX + Mel vs. ABX : OVX + Mel,  p > 0.9999  ABX : Sham vs. ABX : Sham + Mel,  p > 0.9999  ABX : Sham vs. ABX : OVX,  p = 0.0006  ABX : Sham vs. ABX : OVX + Mel,  p = 0.0019  ABX : Sham + Mel vs. ABX : OVX,  p < 0.0001  ABX : Sham + Mel vs. ABX : OVX + Mel  p < 0.0001  ABX : OVX vs. ABX : OVX + Mel  p > 0.9999 | Mean ± s.e.m.  Sham  19.43 ± 0.09  Sham + Mel  19.57 ± 0.18  OVX  20.91 ± 0.18  OVX + Mel  20.97 ± 0.19  Sham + ABX  19.84 ± 0.24  Sham + Mel + ABX  19.43 ± 0.18  OVX + ABX  21.00 ± 0.19  OVX + Mel + ABX  20.90 ± 0.17 |
| **2F**: Weight gain  Sham  (n = 25)  Sham + Mel  (n = 9)  OVX  (n = 14)  OVX + Mel  (n = 14)  Sham + ABX  (n = 10)  Sham + Mel + ABX  (n = 10)  OVX + ABX  (n = 13)  OVX + Mel + ABX  (n = 14) | Three-way ANOVA  ABX :  F (1, 101) = 0.05695,  p = 0.8119  OVX :  F (1, 101) = 100.1,  p < 0.0001  Drug :  F (1, 101) = 4.753,  p = 0.0316  ABX × OVX :  F (1, 101) = 1.641,  p = 0.2031  ABX × Drug :  F (1, 101) = 1.232,  p = 0.2697  OVX × Drug :  F (1, 101) = 0.06285,  p = 0.8026  ABX × OVX × Drug :  F (1, 101) = 0.3260,  p = 0.5693 | | Bonferroni’s multiple comparisons test  Water : Sham vs. Water : Sham + Mel,  p > 0.9999  Water : Sham vs. Water : OVX,  p < 0.0001  Water : Sham vs. Water : OVX + Mel,  p = 0.0004  Water : Sham vs. ABX : Sham,  p > 0.9999  Water : Sham vs. ABX : Sham + Mel,  p > 0.9999  Water : Sham vs. ABX : OVX,  p < 0.0001  Water : Sham vs. ABX : OVX + Mel,  p = 0.0005  Water : Sham + Mel vs. Water : OVX,  p = 0.0004  Water : Sham + Mel vs. Water : OVX + Mel,  p = 0.0014  Water : Sham + Mel vs. ABX : Sham,  p > 0.9999  Water : Sham + Mel vs. ABX : Sham + Mel,  p > 0.9999  Water : Sham + Mel vs. ABX : OVX,  p < 0.0001  Water : Sham + Mel vs. ABX : OVX + Mel,  p = 0.0018  Water : OVX vs. Water : OVX + Mel,  p > 0.9999  Water : OVX vs. ABX : Sham,  p = 0.0011  Water : OVX vs. ABX : Sham + Mel,  p < 0.0001  Water : OVX vs. ABX : OVX,  p > 0.9999  Water : OVX vs. ABX : OVX + Mel,  p > 0.9999  Water : OVX + Mel vs. ABX : Sham,  p = 0.0041  Water : OVX + Mel vs. ABX : Sham + Mel,  p < 0.0001  Water : OVX + Mel vs. ABX : OVX,  p > 0.9999  Water : OVX + Mel vs. ABX : OVX + Mel,  p > 0.9999  ABX : Sham vs. ABX : Sham + Mel,  p > 0.9999  ABX : Sham vs. ABX : OVX,  p < 0.0001  ABX : Sham vs. ABX : OVX + Mel,  p = 0.0054  ABX : Sham + Mel vs. ABX : OVX,  p < 0.0001  ABX : Sham + Mel vs. ABX : OVX + Mel  p < 0.0001  ABX : OVX vs. ABX : OVX + Mel  p = 0.9760 | Mean ± s.e.m.  Sham  2.77 ± 0.14  Sham + Mel  2.52 ± 0.24  OVX  4.30 ± 0.21  OVX + Mel  4.16 ± 0.35  Sham + ABX  2.68 ± 0.18  Sham + Mel + ABX  2.23 ± 0.18  OVX + ABX  4.88 ± 0.20  OVX + Mel + ABX  4.13 ± 0.37 |
| **2G**: Behavioral test of SPT  Sham  (n = 24)  Sham + Mel  (n = 9)  OVX  (n = 14)  OVX + Mel  (n = 13)  Sham + ABX  (n = 10)  Sham + Mel + ABX  (n = 10)  OVX + ABX  (n = 13)  OVX + Mel + ABX  (n = 14) | Three-way ANOVA  ABX:  F (1, 99) = 18.54,  p < 0.0001  OVX:  F (1, 99) = 13.09,  p = 0.0005  Drug:  F (1, 99) = 8.413,  p = 0.0046  ABX × OVX:  F (1, 99) = 1.473,  p = 0.2278  ABX × Drug:  F (1, 99) = 13.76,  p = 0.0003  OVX × Drug:  F (1, 99) = 22.63,  p < 0.0001  ABX × OVX × Drug:  F (1, 99) = 17.71,  p < 0.0001 | | Bonferroni’s multiple comparisons test  Water : Sham vs. Water : Sham + Mel,  p > 0.9999  Water : Sham vs. Water : OVX,  p < 0.0001  Water : Sham vs. Water : OVX + Mel,  p > 0.9999  Water : Sham vs. ABX : Sham,  p = 0.0483  Water : Sham vs. ABX : Sham + Mel,  p = 0.0041  Water : Sham vs. ABX : OVX,  p < 0.0001  Water : Sham vs. ABX : OVX + Mel,  p < 0.0001  Water : Sham + Mel vs. Water : OVX,  p < 0.0001  Water : Sham + Mel vs. Water : OVX + Mel,  p > 0.9999  Water : Sham + Mel vs. ABX : Sham,  p > 0.9999  Water : Sham + Mel vs. ABX : Sham + Mel,  p = 0.8406  Water : Sham + Mel vs. ABX : OVX,  p = 0.0763  Water : Sham + Mel vs. ABX : OVX + Mel,  p = 0.0452  Water : OVX vs. Water : OVX + Mel,  p < 0.0001  Water : OVX vs. ABX : Sham,  p = 0.0074  Water : OVX vs. ABX : Sham + Mel,  p = 0.0654  Water : OVX vs. ABX : OVX,  p = 0.3698  Water : OVX vs. ABX : OVX + Mel,  p = 0.4685  Water : OVX + Mel vs. ABX : Sham,  p = 0.0088  Water : OVX + Mel vs. ABX : Sham + Mel,  p = 0.0008  Water : OVX + Mel vs. ABX : OVX,  p < 0.0001  Water : OVX + Mel vs. ABX : OVX + Mel,  p < 0.0001  ABX : Sham vs. ABX : Sham + Mel,  p > 0.9999  ABX : Sham vs. ABX : OVX,  p > 0.9999  ABX : Sham vs. ABX : OVX + Mel,  p > 0.9999  ABX : Sham + Mel vs. ABX : OVX,  p > 0.9999  ABX : Sham + Mel vs. ABX : OVX + Mel  p > 0.9999  ABX : OVX vs. ABX : OVX + Mel  p > 0.9999 | Mean ± s.e.m.  Sham  76.38 ± 2.19  Sham + Mel  69.96 ± 3.40  OVX  38.75 ± 5.15  OVX + Mel  81.23 ± 3.54  Sham + ABX  59.97 ± 4.25  Sham + Mel + ABX  56.26 ± 3.55  OVX + ABX  51.91 ± 3.14  OVX + Mel + ABX  51.20 ± 4.44 |
| **2H**: Behavioral test of TST  Sham  (n = 24)  Sham + Mel  (n = 9)  OVX  (n = 14)  OVX + Mel  (n = 14)  Sham + ABX  (n = 10)  Sham + Mel + ABX  (n = 10)  OVX + ABX  (n = 13)  OVX + Mel + ABX  (n = 15) | Three-way ANOVA  ABX:  F (1, 101) = 8.023,  p = 0.0056  OVX:  F (1, 101) = 2.302,  p = 0.1323  Drug:  F (1, 101) = 0.007526,  p = 0.9310  ABX × OVX:  F (1, 101) = 1.213,  p = 0.2734  ABX × Drug:  F (1, 101) = 8.291,  p = 0.0049  OVX × Drug:  F (1, 101) = 2.866,  p = 0.0936  ABX × OVX × Drug:  F (1, 101) = 4.349,  p= 0.0395 | | Bonferroni’s multiple comparisons test  Water : Sham vs. Water : Sham + Mel,  p > 0.9999  Water : Sham vs. Water : OVX,  p = 0.0069  Water : Sham vs. Water : OVX + Mel,  p > 0.9999  Water : Sham vs. ABX : Sham,  p > 0.9999  Water : Sham vs. ABX : Sham + Mel,  p = 0.0859  Water : Sham vs. ABX : OVX,  p > 0.9999  Water : Sham vs. ABX : OVX + Mel,  p = 0.0039  Water : Sham + Mel vs. Water : OVX,  p = 0.3611  Water : Sham + Mel vs. Water : OVX + Mel,  p > 0.9999  Water : Sham + Mel vs. ABX : Sham,  p > 0.9999  Water : Sham + Mel vs. ABX : Sham + Mel,  p > 0.9999  Water : Sham + Mel vs. ABX : OVX,  p > 0.9999  Water : Sham + Mel vs. ABX : OVX + Mel,  p = 0.2807  Water : OVX vs. Water : OVX + Mel,  p = 0.0211  Water : OVX vs. ABX : Sham,  p > 0.9999  Water : OVX vs. ABX : Sham + Mel,  p > 0.9999  Water : OVX vs. ABX : OVX,  p > 0.9999  Water : OVX vs. ABX : OVX + Mel,  p > 0.9999  Water : OVX + Mel vs. ABX : Sham,  p > 0.9999  Water : OVX + Mel vs. ABX : Sham + Mel,  p = 0.1518  Water : OVX + Mel vs. ABX : OVX,  p > 0.9999  Water : OVX + Mel vs. ABX : OVX + Mel,  p = 0.0138  ABX : Sham vs. ABX : Sham + Mel,  p > 0.9999  ABX : Sham vs. ABX : OVX,  p > 0.9999  ABX : Sham vs. ABX : OVX + Mel,  p > 0.9999  ABX : Sham + Mel vs. ABX : OVX,  p > 0.9999  ABX : Sham + Mel vs. ABX : OVX + Mel  p > 0.9999  ABX : OVX vs. ABX : OVX + Mel  p > 0.9999 | Mean ± s.e.m.  Sham  146.6 ± 7.0  Sham + Mel  155.1 ± 13.6  OVX  201.7 ± 12.1  OVX + Mel  145.1 ± 9.4  Sham + ABX  173.6 ± 17.4  Sham + Mel + ABX  195.8 ± 17.6  OVX + ABX  173.8 ± 10.8  OVX + Mel + ABX  202.7 ± 12.1 |
| **2I**: Behavioral test of FST  Sham  (n = 24)  Sham + Mel  (n = 9)  OVX  (n = 14)  OVX + Mel  (n = 14)  Sham + ABX  (n = 10)  Sham + Mel + ABX  (n = 10)  OVX + ABX  (n = 13)  OVX + Mel + ABX  (n = 14) | Three-way ANOVA  F (1, 100) = 31.70,  p < 0.0001  OVX:  F (1, 100) = 1.760,  p = 0.1877  Drug:  F (1, 100) = 2.628,  p = 0.1081  ABX × OVX:  F (1, 100) = 4.529,  p = 0.0358  ABX × Drug:  F (1, 100) = 3.990,  p = 0.0485  OVX × Drug:  F (1, 100) = 6.216,  p = 0.0143  ABX × OVX × Drug:  F (1, 100) = 1.692,  p = 0.1964 | | Bonferroni’s multiple comparisons test  Water : Sham vs. Water : Sham + Mel,  p > 0.9999  Water : Sham vs. Water : OVX,  p = 0.0010  Water : Sham vs. Water : OVX + Mel,  p > 0.9999  Water : Sham vs. ABX : Sham,  p = 0.0079  Water : Sham vs. ABX : Sham + Mel,  p = 0.0003  Water : Sham vs. ABX : OVX,  p = 0.0009  Water : Sham vs. ABX : OVX + Mel,  p = 0.0039  Water : Sham + Mel vs. Water : OVX,  p = 0.0350  Water : Sham + Mel vs. Water : OVX + Mel,  p > 0.9999  Water : Sham + Mel vs. ABX : Sham,  p = 0.0934  Water : Sham + Mel vs. ABX : Sham + Mel,  p = 0.0102  Water : Sham + Mel vs. ABX : OVX,  p = 0.0302  Water : Sham + Mel vs. ABX : OVX + Mel,  p = 0.0861  Water : OVX vs. Water : OVX + Mel,  p = 0.0043  Water : OVX vs. ABX : Sham,  p > 0.9999  Water : OVX vs. ABX : Sham + Mel,  p > 0.9999  Water : OVX vs. ABX : OVX,  p > 0.9999  Water : OVX vs. ABX : OVX + Mel,  p > 0.9999  Water : OVX + Mel vs. ABX : Sham,  p = 0.0194  Water : OVX + Mel vs. ABX : Sham + Mel,  p = 0.0013  Water : OVX + Mel vs. ABX : OVX,  p = 0.0038  Water : OVX + Mel vs. ABX : OVX + Mel,  p = 0.0135  ABX : Sham vs. ABX : Sham + Mel,  p > 0.9999  ABX : Sham vs. ABX : OVX,  p > 0.9999  ABX : Sham vs. ABX : OVX + Mel,  p > 0.9999  ABX : Sham + Mel vs. ABX : OVX,  p > 0.9999  ABX : Sham + Mel vs. ABX : OVX + Mel  p > 0.9999  ABX : OVX vs. ABX : OVX + Mel  p > 0.9999 | Mean ± s.e.m.  Sham  88.8 ± 6.9  Sham + Mel  90.1 ± 16.8  OVX  144.0 ± 13.1  OVX + Mel  87.5 ± 9.1  Sham + ABX  142.6 ± 10.9  Sham + Mel + ABX  154.6 ± 6.2  OVX + ABX  145.6 ± 12.1  OVX + Mel + ABX  139.4 ± 8.9 |
| **2J**: Behavioral test of OFT  Sham  (n = 24)  Sham + Mel  (n = 9)  OVX  (n = 14)  OVX + Mel  (n = 14)  Sham + ABX  (n = 10)  Sham + Mel + ABX  (n = 10)  OVX + ABX  (n = 13)  OVX + Mel + ABX  (n = 15) | Three-way ANOVA  ABX:  F (1, 101) = 1.937,  p = 0.1671  OVX:  F (1, 101) = 2.022,  p = 0.1581  Drug:  F (1, 101) = 0.6131,  p = 0.4355  ABX × OVX:  F (1, 101) = 0.03449,  p = 0.8530  ABX × Drug:  F (1, 101) = 0.2147,  p = 0.6441  OVX × Drug:  F (1, 101) = 0.4310,  p = 0.5130  ABX × OVX × Drug:  F (1, 101) = 0.8103,  p = 0.3702 | | Bonferroni’s multiple comparisons test  Water : Sham vs. Water : Sham + Mel,  p > 0.9999  Water : Sham vs. Water : OVX,  p > 0.9999  Water : Sham vs. Water : OVX + Mel,  p > 0.9999  Water : Sham vs. ABX : Sham,  p > 0.9999  Water : Sham vs. ABX : Sham + Mel,  p > 0.9999  Water : Sham vs. ABX : OVX,  p = 0.6867  Water : Sham vs. ABX : OVX + Mel,  p > 0.9999  Water : Sham + Mel vs. Water : OVX,  p > 0.9999  Water : Sham + Mel vs. Water : OVX + Mel,  p > 0.9999  Water : Sham + Mel vs. ABX : Sham,  p > 0.9999  Water : Sham + Mel vs. ABX : Sham + Mel,  p > 0.9999  Water : Sham + Mel vs. ABX : OVX,  p > 0.9999  Water : Sham + Mel vs. ABX : OVX + Mel,  p > 0.9999  Water : OVX vs. Water : OVX + Mel,  p > 0.9999  Water : OVX vs. ABX : Sham,  p > 0.9999  Water : OVX vs. ABX : Sham + Mel,  p > 0.9999  Water : OVX vs. ABX : OVX,  p > 0.9999  Water : OVX vs. ABX : OVX + Mel,  p > 0.9999  Water : OVX + Mel vs. ABX : Sham,  p > 0.9999  Water : OVX + Mel vs. ABX : Sham + Mel,  p > 0.9999  Water : OVX + Mel vs. ABX : OVX,  p > 0.9999  Water : OVX + Mel vs. ABX : OVX + Mel,  p > 0.9999  ABX : Sham vs. ABX : Sham + Mel,  p > 0.9999  ABX : Sham vs. ABX : OVX,  p > 0.9999  ABX : Sham vs. ABX : OVX + Mel,  p > 0.9999  ABX : Sham + Mel vs. ABX : OVX,  p > 0.9999  ABX : Sham + Mel vs. ABX : OVX + Mel  p > 0.9999  ABX : OVX vs. ABX : OVX + Mel  p > 0.9999 | Mean ± s.e.m.  Sham  49.169 ± 2.198  Sham + Mel  46.692 ± 4.477  OVX  42.832 ± 1.456  OVX + Mel  46.590 ± 2.872  Sham + ABX  43.280 ± 2.142  Sham + Mel + ABX  46.263 ± 3.313  OVX + ABX  41.292 ± 3.479  OVX + Mel + ABX  43.300 ±2.014 |
| **2K**: Behavioral test of OFT  Sham  (n = 24)  Sham + Mel  (n = 9)  OVX  (n = 14)  OVX + Mel  (n = 14)  Sham + ABX  (n = 10)  Sham + Mel + ABX  (n = 10)  OVX + ABX  (n = 13)  OVX + Mel + ABX  (n = 15) | Three-way ANOVA  ABX:  F (1, 101) = 0.2880,  p = 0.5927  OVX:  F (1, 101) = 3.008,  p = 0.0859  Drug:  F (1, 101) = 0.01713,  p = 0.8961  ABX × OVX:  F (1, 101) = 0.5685,  p = 0.4526  ABX × Drug:  F (1, 101) = 2.333,  p = 0.1297  OVX × Drug:  F (1, 101) = 0.2006,  p = 0.6552  ABX × OVX × Drug:  F (1, 101) = 1.739,  p = 0.1903 | | Bonferroni’s multiple comparisons test  Water : Sham vs. Water : Sham + Mel,  p > 0.9999  Water : Sham vs. Water : OVX,  p > 0.9999  Water : Sham vs. Water : OVX + Mel,  p > 0.9999  Water : Sham vs. ABX : Sham,  p > 0.9999  Water : Sham vs. ABX : Sham + Mel,  p > 0.9999  Water : Sham vs. ABX : OVX,  p > 0.9999  Water : Sham vs. ABX : OVX + Mel,  p = 0.7283  Water : Sham + Mel vs. Water : OVX,  p > 0.9999  Water : Sham + Mel vs. Water : OVX + Mel,  p > 0.9999  Water : Sham + Mel vs. ABX : Sham,  p > 0.9999  Water : Sham + Mel vs. ABX : Sham + Mel,  p > 0.9999  Water : Sham + Mel vs. ABX : OVX,  p > 0.9999  Water : Sham + Mel vs. ABX : OVX + Mel,  p > 0.9999  Water : OVX vs. Water : OVX + Mel,  p > 0.9999  Water : OVX vs. ABX : Sham,  p > 0.9999  Water : OVX vs. ABX : Sham + Mel,  p > 0.9999  Water : OVX vs. ABX : OVX,  p > 0.9999  Water : OVX vs. ABX : OVX + Mel,  p > 0.9999  Water : OVX + Mel vs. ABX : Sham,  p > 0.9999  Water : OVX + Mel vs. ABX : Sham + Mel,  p > 0.9999  Water : OVX + Mel vs. ABX : OVX,  p > 0.9999  Water : OVX + Mel vs. ABX : OVX + Mel,  p = 0.7925  ABX : Sham vs. ABX : Sham + Mel,  p > 0.9999  ABX : Sham vs. ABX : OVX,  p > 0.9999  ABX : Sham vs. ABX : OVX + Mel,  p > 0.9999  ABX : Sham + Mel vs. ABX : OVX,  p > 0.9999  ABX : Sham + Mel vs. ABX : OVX + Mel  p > 0.9999  ABX : OVX vs. ABX : OVX + Mel  p > 0.9999 | Mean ± s.e.m.  Sham  6.066 ± 0.431  Sham + Mel  5.930 ± 0.509  OVX  5.061 ± 0.396  OVX + Mel  6.218 ± 0.540  Sham + ABX  6.222 ± 0.562  Sham + Mel + ABX  5.933 ± 0.673  OVX + ABX  5.630 ± 0.471  OVX + Mel + ABX  4.704 ± 0.390 |
| **2L**: Colonic melatonin level  Sham  (n = 9)  Sham + Mel  (n = 5)  OVX  (n = 6)  OVX + Mel  (n = 6)  Sham + ABX  (n = 7)  Sham + Mel + ABX  (n = 7)  OVX + ABX  (n = 6)  OVX + Mel + ABX  (n = 6) | Three-way ANOVA  ABX:  F (1, 44) = 76.40,  p < 0.0001  OVX:  F (1, 44) = 11.62,  p = 0.0014  Drug:  F (1, 44) = 2.277,  p = 0.1385  ABX × OVX:  F (1, 44) = 7.406,  p = 0.0093  ABX × Drug:  F (1, 44) = 0.7017,  p = 0.4067  OVX × Drug:  F (1, 44) = 5.920,  p = 0.0191  ABX × OVX × Drug:  F (1, 44) = 4.818,  p = 0.0335 | | Bonferroni’s multiple comparisons test  Water : Sham vs. Water : Sham + Mel,  p > 0.9999  Water : Sham vs. Water : OVX,  p < 0.0001  Water : Sham vs. Water : OVX + Mel,  p > 0.9999  Water : Sham vs. ABX : Sham,  p < 0.0001  Water : Sham vs. ABX : Sham + Mel,  p < 0.0001  Water : Sham vs. ABX : OVX,  p < 0.0001  Water : Sham vs. ABX : OVX + Mel,  p < 0.0001  Water : Sham + Mel vs. Water : OVX,  p = 0.0082  Water : Sham + Mel vs. Water : OVX + Mel,  p > 0.9999  Water : Sham + Mel vs. ABX : Sham,  p = 0.0002  Water : Sham + Mel vs. ABX : Sham + Mel,  p = 0.0004  Water : Sham + Mel vs. ABX : OVX,  p < 0.0001  Water : Sham + Mel vs. ABX : OVX + Mel,  p = 0.0004  Water : OVX vs. Water : OVX + Mel,  p = 0.0410  Water : OVX vs. ABX : Sham,  p > 0.9999  Water : OVX vs. ABX : Sham + Mel,  p > 0.9999  Water : OVX vs. ABX : OVX,  p > 0.9999  Water : OVX vs. ABX : OVX + Mel,  p > 0.9999  Water : OVX + Mel vs. ABX : Sham,  p = 0.0011  Water : OVX + Mel vs. ABX : Sham + Mel,  p = 0.0023  Water : OVX + Mel vs. ABX : OVX,  p = 0.0004  Water : OVX + Mel vs. ABX : OVX + Mel,  p = 0.0019  ABX : Sham vs. ABX : Sham + Mel,  p > 0.9999  ABX : Sham vs. ABX : OVX,  p > 0.9999  ABX : Sham vs. ABX : OVX + Mel,  p > 0.9999  ABX : Sham + Mel vs. ABX : OVX,  p > 0.9999  ABX : Sham + Mel vs. ABX : OVX + Mel  p > 0.9999  ABX : OVX vs. ABX : OVX + Mel  p > 0.9999 | Mean ± s.e.m.  Sham  1.0000 ± 0.0353  Sham + Mel  0.9149 ± 0.0468  OVX  0.5986 ± 0.0578  OVX + Mel  0.8588 ± 0.0817  Sham + ABX  0.5217 ± 0.0334  Sham + Mel + ABX  0.5378 ± 0.0359  OVX + ABX  0.4871 ± 0.0459  OVX + Mel + ABX  0.5210 ± 0.0769 |
| **3B**: Behavioral test of SPT  Sham  (n = 10)  OVX  (n = 10)  OVX + Veh  (n = 10)  OVX + Adolescent-FMT  (n = 10) | One-way ANOVA  Treatment:  F (3, 36) = 22.40,  p < 0.0001 | | Bonferroni’s multiple comparisons test  Sham vs. OVX,  p < 0.0001  Sham vs. OVX + Veh,  p < 0.0001  Sham vs. OVX + Adolescent-FMT,  p > 0.9999  OVX vs. OVX + Veh,  p > 0.9999  OVX vs. OVX + Adolescent-FMT,  p < 0.0001  OVX + Veh vs. OVX + Adolescent-FMT,  p < 0.0001 | Mean ± s.e.m.  Sham  82.90 ± 2.17  OVX  51.72 ± 4.16  OVX + Veh  50.90 ± 4.34  OVX + Adolescent-FMT  81.09 ± 3.91 |
| **3C**: Behavioral test of TST  Sham  (n = 10)  OVX  (n = 10)  OVX + Veh  (n = 10)  OVX + Adolescent-FMT  (n = 10) | One-way ANOVA  Treatment:  F (3, 36) = 6.213,  p = 0.0016 | | Bonferroni’s multiple comparisons test  Sham vs. OVX,  p = 0.0018  Sham vs. OVX + Veh,  p = 0.0981  Sham vs. OVX + Adolescent-FMT,  p > 0.9999  OVX vs. OVX + Veh,  p = 0.8955  OVX vs. OVX + Adolescent-FMT,  p = 0.0243  OVX + Veh vs. OVX + Adolescent-FMT,  p = 0.7152 | Mean ± s.e.m.  Sham  119.1 ± 15.9  OVX  199.6 ± 15.8  OVX + Veh  169.9 ± 14.1  OVX + Adolescent-FMT  137.7 ± 10.7 |
| **3D**: Behavioral test of FST  Sham  (n = 10)  OVX  (n = 9)  OVX + Veh  (n = 9)  OVX + Adolescent-FMT  (n = 10) | One-way ANOVA  Treatment:  F (3, 34) = 6.433,  p = 0.0014 | | Bonferroni’s multiple comparisons test  Sham vs. OVX,  p = 0.0119  Sham vs. OVX + Veh,  p = 0.1009  Sham vs. OVX + Adolescent-FMT,  p > 0.9999  OVX vs. OVX + Veh,  p > 0.9999  OVX vs. OVX + Adolescent-FMT,  p = 0.0063  OVX + Veh vs. OVX + Adolescent-FMT,  p = 0.0574 | Mean ± s.e.m.  Sham  103.6 ± 13.0  OVX  152.3 ± 10.8  OVX + Veh  140.1 ± 8.3  OVX + Adolescent-FMT  100.2 ± 7.9 |
| **3E**: Behavioral test of OFT  Sham  (n = 10)  OVX  (n = 10)  OVX + Veh  (n = 10)  OVX + Adolescent-FMT  (n = 10) | One-way ANOVA  Treatment:  F (3, 36) = 1.254,  p = 0.3048 | | Bonferroni’s multiple comparisons test  Sham vs. OVX,  p = 0.8363  Sham vs. OVX + Veh,  p = 0.7770  Sham vs. OVX + Adolescent-FMT,  p > 0.9999  OVX vs. OVX + Veh,  p > 0.9999  OVX vs. OVX + Adolescent-FMT,  p > 0.9999  OVX + Veh vs. OVX + Adolescent-FMT,  p > 0.9999 | Mean ± s.e.m.  Sham  36.263 ± 3.110  OVX  30.766 ± 2.114  OVX + Veh  30.621 ± 2.753  OVX + Adolescent-FMT  34.909 ± 2.173 |
| **3F**: Behavioral test of OFT  Sham  (n = 10)  OVX  (n = 10)  OVX + Veh  (n = 10)  OVX + Adolescent-FMT  (n = 10) | One-way ANOVA  Treatment:  F (3, 36) = 1.697,  p = 0.1849 | | Bonferroni’s multiple comparisons test  Sham vs. OVX,  p = 0.3549  Sham vs. OVX + Veh,  p = 0.4650  Sham vs. OVX + Adolescent-FMT,  p > 0.9999  OVX vs. OVX + Veh,  p > 0.9999  OVX vs. OVX + Adolescent-FMT,  p > 0.9999  OVX + Veh vs. OVX + Adolescent-FMT,  p > 0.9999 | Mean ± s.e.m.  Sham  5.514 ± 0.690  OVX  3.897 ± 0.359  OVX + Veh  4.006 ± 0.603  OVX + Adolescent-FMT  4.871 ± 0.639 |
| **3G**: Colonic melatonin level  Sham  (n = 19)  OVX  (n = 14)  OVX + Veh  (n = 13)  OVX + Adolescent-FMT  (n = 13) | One-way ANOVA  Treatment:  F (3, 55) = 27.36,  p < 0.0001 | | Bonferroni’s multiple comparisons test  Sham vs. OVX,  p < 0.0001  Sham vs. OVX + Veh,  p < 0.0001  Sham vs. OVX + Adolescent-FMT,  p =0.0003  OVX vs. OVX + Veh,  p = 0.5088  OVX vs. OVX + Adolescent-FMT,  p = 0.0041  OVX + Veh vs. OVX + Adolescent-FMT,  p = 0.4537 | Mean ± s.e.m.  Sham  1.0000 ± 0.0473  OVX  0.4865 ± 0.0432  OVX + Veh  0.6027 ± 0.0519  OVX + Adolescent-FMT  0.7248 ±0.0253 |
| **3I**: Behavioral test of SPT  V-FMT  (n = 10)  Sham-FMT  (n = 10)  OVX-FMT  (n = 10)  OVX + Mel-FMT  (n = 11) | One-way ANOVA  Treatment:  F (3, 37) = 56.81,  p < 0.0001 | | Bonferroni’s multiple comparisons test  V-FMT vs. Sham-FMT,  p >0.9999  V-FMT vs. OVX-FMT,  p < 0.0001  V-FMT vs. OVX + Mel-FMT,  p > 0.9999  Sham-FMT vs. OVX-FMT,  p < 0.0001  Sham-FMT vs. OVX + Mel-FMT,  p > 0.9999  OVX-FMT vs. OVX + Mel-FMT,  p < 0.0001 | Mean ± s.e.m.  V-FMT  87.02 ± 2.75  Sham-FMT  84.32 ± 2.83  OVX-FMT  36.40 ± 4.57  OVX + Mel-FMT  88.62 ± 2.86 |
| **3J**: Behavioral test of TST  V-FMT  (n = 10)  Sham-FMT  (n = 10)  OVX-FMT  (n = 10)  OVX + Mel-FMT  (n = 11) | One-way ANOVA  Treatment:  F (3, 37) = 3.668,  p = 0.0207 | | Bonferroni’s multiple comparisons test  V-FMT vs. Sham-FMT,  p >0.9999  V-FMT vs. OVX-FMT,  p = 0.2453  V-FMT vs. OVX + Mel-FMT,  p > 0.9999  Sham-FMT vs. OVX-FMT,  p = 0.0449  Sham-FMT vs. OVX + Mel-FMT,  p > 0.9999  OVX-FMT vs. OVX + Mel-FMT,  p = 0.0354 | Mean ± s.e.m.  V-FMT  152.9 ± 11.9  Sham-FMT  140.8 ± 12.2  OVX-FMT  189.1 ± 10.9  OVX + Mel-FMT  140.3 ± 12.6 |
| **3K**: Behavioral test of FST  V-FMT  (n = 10)  Sham-FMT  (n = 10)  OVX-FMT  (n = 9)  OVX + Mel-FMT  (n = 11) | One-way ANOVA  Treatment:  F (3, 36) = 8.710,  p = 0.0002 | | Bonferroni’s multiple comparisons test  V-FMT vs. Sham-FMT,  p >0.9999  V-FMT vs. OVX-FMT,  p = 0.0006  V-FMT vs. OVX + Mel-FMT,  p > 0.9999  Sham-FMT vs. OVX-FMT,  p = 0.0166  Sham-FMT vs. OVX + Mel-FMT,  p > 0.9999  OVX-FMT vs. OVX + Mel-FMT,  p = 0.0003 | Mean ± s.e.m.  V-FMT  87.5 ± 8.2  Sham-FMT  101.0 ± 7.7  OVX-FMT  138.1 ± 7.6  OVX + Mel-FMT   \|  \| \| --- \|   86.4 ± 8.2 |
| **3L**: Behavioral test of OFT  V-FMT  (n = 10)  Sham-FMT  (n = 10)  OVX-FMT  (n = 10)  OVX + Mel-FMT  (n = 11) | One-way ANOVA  Treatment:  F (3, 37) = 0.5323,  p = 0.6630 | | Bonferroni’s multiple comparisons test  V-FMT vs. Sham-FMT,  p >0.9999  V-FMT vs. OVX-FMT,  p >0.9999  V-FMT vs. OVX + Mel-FMT,  p > 0.9999  Sham-FMT vs. OVX-FMT,  p >0.9999  Sham-FMT vs. OVX + Mel-FMT,  p > 0.9999  OVX-FMT vs. OVX + Mel-FMT,  p >0.9999 | Mean ± s.e.m.  V-FMT  44.250 ± 2.778  Sham-FMT  43.617 ± 2.180  OVX-FMT  40.263 ± 3.418  OVX + Mel-FMT   \|  \| \| --- \|   41.591 ± 1.307 |
| **3M**: Behavioral test of OFT  V-FMT  (n = 10)  Sham-FMT  (n = 10)  OVX-FMT  (n = 10)  OVX + Mel-FMT  (n = 11) | One-way ANOVA  Treatment:  F (3, 37) = 0.8509,  p = 0.4750 | | Bonferroni’s multiple comparisons test  V-FMT vs. Sham-FMT,  p >0.9999  V-FMT vs. OVX-FMT,  p >0.9999  V-FMT vs. OVX + Mel-FMT,  p > 0.9999  Sham-FMT vs. OVX-FMT,  p >0.9999  Sham-FMT vs. OVX + Mel-FMT,  p = 0.9171  OVX-FMT vs. OVX + Mel-FMT,  p >0.9999 | Mean ± s.e.m.  V-FMT  5.458 ± 0.714  Sham-FMT  5.719 ± 0.445  OVX-FMT  4.967 ± 0.643  OVX + Mel-FMT   \|  \| \| --- \|   4.619 ± 0.286 |
| **3N**: Serum estradiol level  V-FMT  (n = 7)  Sham-FMT  (n = 7)  OVX-FMT  (n = 8)  OVX + Mel-FMT  (n = 7) | One-way ANOVA  Treatment:  F (3, 25) = 0.6558,  p = 0.5869 | | Bonferroni’s multiple comparisons test  V-FMT vs. Sham-FMT,  p > 0.9999  V-FMT vs. OVX-FMT,  p > 0.9999  V-FMT vs. OVX + Mel-FMT,  p > 0.9999  Sham-FMT vs. OVX-FMT,  p > 0.9999  Sham-FMT vs. OVX + Mel-FMT,  p > 0.9999  OVX-FMT vs. OVX + Mel-FMT,  p > 0.9999 | Mean ± s.e.m.  V-FMT  1.0302 ± 0.0326  Sham-FMT  1.0000 ± 0.0518  OVX-FMT  1.0478 ± 0.0406  OVX + Mel-FMT   \|  \| \| --- \|   1.0762 ± 0.0227 |
| **5E**: Fecal *Alistipes Inops* abundance  Sham  (n = 5)  OVX  (n = 6)  OVX + Mel  (n = 5) | One-way ANOVA  Treatment:  F (2, 13) = 25.51,  p < 0.0001 | | Bonferroni’s multiple comparisons test  Sham vs. OVX,  p < 0.0001  Sham vs. OVX + Mel,  p = 0.0110  OVX vs. OVX + Mel,  p = 0.0130 | Mean ± s.e.m.  Sham  1.0000 ± 0.2288  OVX  13.3228 ± 1.5444  OVX + Mel  7.3731 ± 1.2853 |
| **5F**: *Alistipes Inops* growth curve  Control (n = 3)  1 mM Mel (n = 3)  2 mM Mel (n = 3)  4 mM Mel (n = 3)  8 mM Mel (n = 3) | Two-way ANOVA  Time × Drug:  F (52, 130) = 11579,  p < 0.0001  Time:  F (3.229, 32.29) = 45154,  p < 0.0001  Drug:  F (4, 10) = 75722,  p < 0.0001  Subject:  F (10, 130) = 2.674,  p = 0.0053 | | Bonferroni’s multiple comparisons test  Row 1  Control vs. 1 mM Mel, p = 0.0004  Control vs. 2 mM Mel, p = 0.0018  Control vs. 4 mM Mel, p = 0.0019  Control vs. 8 mM Mel, p = 0.0056    Row 2  Control vs. 1 mM Mel, p = 0.0019  Control vs. 2 mM Mel, p = 0.0115  Control vs. 4 mM Mel, p = 0.0070  Control vs. 8 mM Mel, p = 0.0067    Row 3  Control vs. 1 mM Mel, p < 0.0001  Control vs. 2 mM Mel, p < 0.0001  Control vs. 4 mM Mel, p < 0.0001  Control vs. 8 mM Mel, p < 0.0001    Row 4  Control vs. 1 mM Mel, p = 0.0005  Control vs. 2 mM Mel, p < 0.0001  Control vs. 4 mM Mel, p < 0.0001  Control vs. 8 mM Mel, p < 0.0001    Row 5  Control vs. 1 mM Mel, p = 0.0034  Control vs. 2 mM Mel, p = 0.0004  Control vs. 4 mM Mel, p = 0.0009  Control vs. 8 mM Mel, p = 0.0013    Row 6  Control vs. 1 mM Mel, p = 0.0011  Control vs. 2 mM Mel, p < 0.0001  Control vs. 4 mM Mel, p < 0.0001  Control vs. 8 mM Mel, p < 0.0001    Row 7  Control vs. 1 mM Mel, p < 0.0001  Control vs. 2 mM Mel, p < 0.0001  Control vs. 4 mM Mel, p < 0.0001  Control vs. 8 mM Mel, p < 0.0001    Row 8  Control vs. 1 mM Mel, p = 0.0042  Control vs. 2 mM Mel, p = 0.0016  Control vs. 4 mM Mel, p = 0.0015  Control vs. 8 mM Mel, p = 0.0014    Row 9  Control vs. 1 mM Mel, p = 0.0001  Control vs. 2 mM Mel, p = 0.0001  Control vs. 4 mM Mel, p < 0.0001  Control vs. 8 mM Mel, p < 0.0001    Row 10  Control vs. 1 mM Mel, p = 0.0017  Control vs. 2 mM Mel, p < 0.0001  Control vs. 4 mM Mel, p = 0.0007  Control vs. 8 mM Mel, p = 0.0007    Row 11  Control vs. 1 mM Mel, p = 0.0005  Control vs. 2 mM Mel, p < 0.0001  Control vs. 4 mM Mel, p < 0.0001  Control vs. 8 mM Mel, p < 0.0001    Row 12  Control vs. 1 mM Mel, p < 0.0001  Control vs. 2 mM Mel, p < 0.0001  Control vs. 4 mM Mel, p < 0.0001  Control vs. 8 mM Mel, p < 0.0001    Row 13  Control vs. 1 mM Mel, p = 0.0001  Control vs. 2 mM Mel, p < 0.0001  Control vs. 4 mM Mel, p < 0.0001  Control vs. 8 mM Mel, p < 0.0001    Row 14  Control vs. 1 mM Mel, p < 0.0001  Control vs. 2 mM Mel, p < 0.0001  Control vs. 4 mM Mel, p < 0.0001  Control vs. 8 mM Mel, p < 0.0001 | Mean ± s.e.m.  Row 1:  Control  0.036 ± 0.002  1 mM Mel  -0.031 ± 0.001  2 mM Mel  -0.030 ± 0.001  4 mM Mel  -0.029 ± 0.001  8 mM Mel  -0.021 ± 0.000    Row 2:  Control  0.048 ± 0.002  1 mM Mel  0.013 ± 0.001  2 mM Mel  0.003 ± 0.000  4 mM Mel  0.002 ± 0.000  8 mM Mel  0.001 ± 0.000    Row 3:  Control  0.105 ± 0.000  1 mM Mel  0.025 ± 0.000  2 mM Mel  0.005 ± 0.000  4 mM Mel  0.005 ± 0.000  8 mM Mel  0.002 ± 0.000    Row 4:  Control  0.135 ± 0.001  1 mM Mel  0.052 ± 0.001  2 mM Mel  0.005 ± 0.000  4 mM Mel  0.005 ± 0.000  8 mM Mel  0.004 ± 0.000    Row 5:  Control  0.186 ± 0.002  1 mM Mel  0.075 ± 0.000  2 mM Mel  0.005 ± 0.001  4 mM Mel  0.004 ± 0.000  8 mM Mel  0.005 ± 0.000    Row 6:  Control  0.241 ± 0.001  1 mM Mel  0.100 ± 0.003  2 mM Mel  0.011 ± 0.000  4 mM Mel  0.007 ± 0.000  8 mM Mel  0.005 ± 0.000    Row 7:  Control  0.415 ± 0.001  1 mM Mel  0.214 ± 0.002  2 mM Mel  0.046 ± 0.000  4 mM Mel  0.008 ± 0.001  8 mM Mel  0.008 ± 0.001    Row 8:  Control  0.718 ± 0.009  1 mM Mel  0.325 ± 0.001  2 mM Mel  0.094 ± 0.001  4 mM Mel  0.014 ± 0.001  8 mM Mel  0.015 ± 0.001    Row 9:  Control  1.153 ± 0.007  1 mM Mel  0.426 ± 0.002  2 mM Mel  0.149 ± 0.002  4 mM Mel  0.025 ± 0.002  8 mM Mel  0.022 ± 0.002    Row 10:  Control  1.420 ± 0.012  1 mM Mel  0.574 ± 0.002  2 mM Mel  0.195 ± 0.007  4 mM Mel  0.038 ± 0.001  8 mM Mel  0.023 ± 0.000    Row 11:  Control  1.614 ± 0.002  1 mM Mel  0.766 ± 0.008  2 mM Mel  0.231 ± 0.003  4 mM Mel  0.080 ± 0.002  8 mM Mel  0.031 ± 0.001    Row 12:  Control  1.709 ± 0.004  1 mM Mel  0.976 ± 0.008  2 mM Mel  0.273 ± 0.007  4 mM Mel  0.094 ± 0.001  8 mM Mel  0.047 ± 0.001    Row 13:  Control  1.726 ± 0.001  1 mM Mel  1.089 ± 0.003  2 mM Mel  0.338 ± 0.001  4 mM Mel  0.095 ± 0.001  8 mM Mel  0.056 ± 0.002    Row 14:  Control  1.738 ± 0.001  1 mM Mel  1.038 ± 0.003  2 mM Mel  0.338 ± 0.001  4 mM Mel  0.098 ± 0.001  8 mM Mel  0.071 ± 0.001 |
| **6A**: Fecal tryptophan level  Sham  (n = 5)  Sham + Mel  (n = 7)  OVX  (n = 6)  OVX + Mel  (n = 6) | Two-way ANOVA  Interaction:  F (1, 20) = 71.76,  p < 0.0001  Drug:  F (1, 20) = 63.68,  p < 0.0001  OVX:  F (1, 20) = 78.42,  p < 0.0001 | | Bonferroni’s multiple comparisons test  Vehicle : Sham vs. Vehicle : OVX,  p < 0.0001  Vehicle : Sham vs. Melatonin : Sham,  p > 0.9999  Vehicle : Sham vs. Melatonin : OVX,  p > 0.9999  Vehicle : OVX vs. Melatonin : Sham,  p < 0.0001  Vehicle : OVX vs. Melatonin : OVX,  p < 0.0001  Melatonin : Sham vs. Melatonin : OVX,  p > 0.9999 | Mean ± s.e.m.  Sham  1.0000 ± 0.0390  Sham + Mel  0.9888 ± 0.0126  OVX  0.6043 ± 0.0264  OVX + Mel  0.9800 ± 0.0112 |
| **6B**: Colonic tryptophan level  Sham  (n = 8)  Sham + Mel  (n = 7)  OVX  (n = 8)  OVX + Mel  (n = 7) | Two-way ANOVA  Interaction:  F (1, 26) = 5.459,  p = 0.0275  Drug:  F (1, 26) = 6.345,  p = 0.0183  OVX:  F (1, 26) = 5.127,  p = 0.0321 | | Bonferroni’s multiple comparisons test  Vehicle : Sham vs. Vehicle : OVX,  p = 0.0142  Vehicle : Sham vs. Melatonin : Sham,  p > 0.9999  Vehicle : Sham vs. Melatonin : OVX,  p > 0.9999  Vehicle : OVX vs. Melatonin : Sham,  p = 0.0137  Vehicle : OVX vs. Melatonin : OVX,  p = 0.0121  Melatonin : Sham vs. Melatonin : OVX,  p > 0.9999 | Mean ± s.e.m.  Sham  1.0000 ± 0.0696  Sham + Mel  1.0148 ± 0.0439  OVX  0.6278 ± 0.0563  OVX + Mel  1.0206 ± 0.1328 |
| **6C**: Serum tryptophan level  Sham  (n = 8)  Sham + Mel  (n = 7)  OVX  (n = 9)  OVX + Mel  (n = 6) | Two-way ANOVA  Interaction:  F (1, 26) = 0.1731,  p = 0.6808  Drug:  F (1, 26) = 31.41,  p < 0.0001  OVX:  F (1, 26) = 103.3,  p < 0.0001 | | Bonferroni’s multiple comparisons test  Vehicle : Sham vs. Vehicle : OVX,  p < 0.0001  Vehicle : Sham vs. Melatonin : Sham,  p = 0.0013  Vehicle : Sham vs. Melatonin : OVX,  p = 0.0264  Vehicle : OVX vs. Melatonin : Sham,  p < 0.0001  Vehicle : OVX vs. Melatonin : OVX,  p = 0.0072  Melatonin : Sham vs. Melatonin : OVX,  p < 0.0001 | Mean ± s.e.m.  Sham  1.0000 ± 0.0158  Sham + Mel  1.1245 ± 0.0263  OVX  0.7985 ± 0.0204  OVX + Mel  0.9057 ± 0.0169 |
| **6D**: Serum 5-HT level  Sham  (n = 8)  Sham + Mel  (n = 7)  OVX  (n = 9)  OVX + Mel  (n = 6) | Two-way ANOVA  Interaction:  F (1, 26) = 1.614,  p = 0.2151  Drug:  F (1, 26) = 10.53,  p = 0.0032  OVX:  F (1, 26) = 36.71,  p < 0.0001 | | Bonferroni’s multiple comparisons test  Vehicle : Sham vs. Vehicle : OVX,  p < 0.0001  Vehicle : Sham vs. Melatonin : Sham,  p > 0.9999  Vehicle : Sham vs. Melatonin : OVX,  p = 0.3914  Vehicle : OVX vs. Melatonin : Sham,  p < 0.0001  Vehicle : OVX vs. Melatonin : OVX,  p = 0.0236  Melatonin : Sham vs. Melatonin : OVX,  p = 0.0228 | Mean ± s.e.m.  Sham  1.0000 ± 0.0232  Sham + Mel  1.0390 ± 0.0171  OVX  0.8553 ± 0.0162  OVX + Mel  0.9444 ± 0.0209 |
| **6E**: NAc tryptophan level  Sham  (n = 12)  Sham + Mel  (n = 11)  OVX  (n = 10)  OVX + Mel  (n = 11) | Two-way ANOVA  Interaction:  F (1, 40) = 4.069,  p = 0.0504  Drug:  F (1, 40) = 4.435,  p = 0.0415  OVX:  F (1, 40) = 13.42,  p = 0.0007 | | Bonferroni’s multiple comparisons test  Vehicle : Sham vs. Vehicle : OVX,  p = 0.0016  Vehicle : Sham vs. Melatonin : Sham,  p > 0.9999  Vehicle : Sham vs. Melatonin : OVX,  p > 0.9999  Vehicle : OVX vs. Melatonin : Sham,  p = 0.0016  Vehicle : OVX vs. Melatonin : OVX,  p = 0.0412  Melatonin : Sham vs. Melatonin : OVX,  p > 0.9999 | Mean ± s.e.m.  Sham  1.0000 ± 0.0973  Sham + Mel  1.0066 ± 0.0593  OVX  0.5754 ± 0.0612  OVX + Mel  0.8836 ± 0.0657 |
| **6F**: NAc 5-HT level  Sham  (n = 11)  Sham + Mel  (n = 11)  OVX  (n = 10)  OVX + Mel  (n = 11) | Two-way ANOVA  Interaction:  F (1, 39) = 1.820,  p = 0.1851  Drug:  F (1, 39) = 9.523,  p = 0.0037  OVX:  F (1, 39) = 20.47,  p < 0.0001 | | Bonferroni’s multiple comparisons test  Vehicle : Sham vs. Vehicle : OVX,  p = 0.0012  Vehicle : Sham vs. Melatonin : Sham,  p > 0.9999  Vehicle : Sham vs. Melatonin : OVX,  p > 0.9999  Vehicle : OVX vs. Melatonin : Sham,  p < 0.0001  Vehicle : OVX vs. Melatonin : OVX,  p = 0.0216  Melatonin : Sham vs. Melatonin : OVX,  p = 0.1717 | Mean ± s.e.m.  Sham  1.0000 ± 0.0725  Sham + Mel  1.1246 ± 0.0924  OVX  0.5788 ± 0.0500  OVX + Mel  0.8968 ± 0.0611 |
| **6G**: Fecal tryptophan level  V-FMT  (n = 6)  Sham-FMT  (n = 6)  OVX-FMT  (n = 6)  OVX + Mel-FMT  (n = 6) | One-way ANOVA  Treatment:  F (3, 20) = 37.49,  p < 0.0001 | | Bonferroni’s multiple comparisons test  V-FMT vs. Sham-FMT,  p > 0.9999  V-FMT vs. OVX-FMT,  p < 0.0001  V-FMT vs. OVX + Mel-FMT,  p > 0.9999  Sham-FMT vs. OVX-FMT,  p < 0.0001  Sham-FMT vs. OVX + Mel-FMT,  p > 0.9999  OVX-FMT vs. OVX + Mel-FMT,  p < 0.0001 | Mean ± s.e.m.  V-FMT  0.9762 ± 0.0481  Sham-FMT  1.0000 ± 0.0205  OVX-FMT  0.6169 ± 0.0258  OVX + Mel-FMT  1.0355 ± 0.0258 |
| **6H**: Colonic tryptophan level  V-FMT  (n = 7)  Sham-FMT  (n = 7)  OVX-FMT  (n = 8)  OVX + Mel-FMT  (n = 7) | One-way ANOVA  Treatment:  F (3, 25) = 9.536,  p = 0.0002 | | Bonferroni’s multiple comparisons test  V-FMT vs. Sham-FMT,  p > 0.9999  V-FMT vs. OVX-FMT,  p = 0.0002  V-FMT vs. OVX + Mel-FMT,  p = 0.4275  Sham-FMT vs. OVX-FMT,  p = 0.0048  Sham-FMT vs. OVX + Mel-FMT,  p > 0.9999  OVX-FMT vs. OVX + Mel-FMT,  p = 0.0261 | Mean ± s.e.m.  V-FMT  1.1641 ± 0.1426  Sham-FMT  1.0000 ± 0.0923  OVX-FMT  0.5074 ± 0.0541  OVX + Mel-FMT  0.9126 ± 0.0661 |
| **6I**: Serum tryptophan level  V-FMT  (n = 7)  Sham-FMT  (n = 7)  OVX-FMT  (n = 8)  OVX + Mel-FMT  (n = 7) | One-way ANOVA  Treatment:  F (3, 25) = 49.48,  p < 0.0001 | | Bonferroni’s multiple comparisons test  V-FMT vs. Sham-FMT,  p > 0.9999  V-FMT vs. OVX-FMT,  p < 0.0001  V-FMT vs. OVX + Mel-FMT,  p > 0.9999  Sham-FMT vs. OVX-FMT,  p < 0.0001  Sham-FMT vs. OVX + Mel-FMT,  p = 0.3856  OVX-FMT vs. OVX + Mel-FMT,  p < 0.0001 | Mean ± s.e.m.  V-FMT  0.9639 ± 0.0115  Sham-FMT  1.0000 ± 0.0418  OVX-FMT  0.6542 ± 0.0075  OVX + Mel-FMT  0.9354 ± 0.0192 |
| **6J**: Serum 5-HT level  V-FMT  (n = 7)  Sham-FMT  (n = 7)  OVX-FMT  (n = 8)  OVX + Mel-FMT  (n = 7) | One-way ANOVA  Treatment:  F (3, 25) = 35.81,  p < 0.0001 | | Bonferroni’s multiple comparisons test  V-FMT vs. Sham-FMT,  p > 0.9999  V-FMT vs. OVX-FMT,  p < 0.0001  V-FMT vs. OVX + Mel-FMT,  p = 0.9296  Sham-FMT vs. OVX-FMT,  p < 0.0001  Sham-FMT vs. OVX + Mel-FMT,  p = 0.0868  OVX-FMT vs. OVX + Mel-FMT,  p < 0.0001 | Mean ± s.e.m.  V-FMT  1.0647 ± 0.0427  Sham-FMT  1.0000 ± 0.0421  OVX-FMT  0.6362 ± 0.0212  OVX + Mel-FMT  1.1464 ± 0.0476 |
| **6K**: NAc tryptophan level  V-FMT  (n = 5)  Sham-FMT  (n = 7)  OVX-FMT  (n = 8)  OVX + Mel-FMT  (n = 6) | One-way ANOVA  Treatment:  F (3, 22) = 12.69,  p < 0.0001 | | Bonferroni’s multiple comparisons test  V-FMT vs. Sham-FMT,  p > 0.9999  V-FMT vs. OVX-FMT,  p = 0.0221  V-FMT vs. OVX + Mel-FMT,  p = 0.1541  Sham-FMT vs. OVX-FMT,  p = 0.0612  Sham-FMT vs. OVX + Mel-FMT,  p = 0.0187  OVX-FMT vs. OVX + Mel-FMT,  p < 0.0001 | Mean ± s.e.m.  V-FMT  1.0611 ± 0.0569  Sham-FMT  1.0000 ± 0.0755  OVX-FMT  0.7762 ± 0.0504  OVX + Mel-FMT  1.2841 ± 0.0498 |
| **6L**: NAc 5-HT level  V-FMT  (n = 7)  Sham-FMT  (n = 6)  OVX-FMT  (n = 8)  OVX + Mel-FMT  (n = 7) | One-way ANOVA  Treatment:  F (3, 24) = 8.237,  p = 0.0006 | | Bonferroni’s multiple comparisons test  V-FMT vs. Sham-FMT,  p = 0.0702  V-FMT vs. OVX-FMT,  p = 0.2431  V-FMT vs. OVX + Mel-FMT,  p > 0.9999  Sham-FMT vs. OVX-FMT,  p = 0.0003  Sham-FMT vs. OVX + Mel-FMT,  p = 0.3420  OVX-FMT vs. OVX + Mel-FMT,  p = 0.0420 | Mean ± s.e.m.  V-FMT  0.7793 ± 0.0697  Sham-FMT  1.0000 ± 0.0449  OVX-FMT  0.6164 ± 0.0537  OVX + Mel-FMT  0.8383 ± 0.0439 |
| **7B**: Fecal *Alistipes Inops* abundance  per total 16S rDNA  Con (n = 7)  *Alis* (n = 5) | Unpaired t test  t = 5.406,  p = 0.0003 | |  | Mean ± s.e.m.  Con  0.4286 ± 0.2037  *Alis*  181.6360 ± 40.4768 |
| **7C**: Behavioral test of SPT  Con  (n = 17)  *Alis*  (n = 16)  *Alis* + Mel  (n = 16)  *Alis* + Trp  (n = 17)  *Alis* + Mel + Trp  (n = 19) | One-way ANOVA  Treatment:  F (4, 80) = 36.29,  p < 0.0001 | | Bonferroni’s multiple comparisons test  Con vs. *Alis*,  p < 0.0001  Con vs. *Alis* + Mel,  p > 0.9999  Con vs. *Alis* + Trp,  p = 0.0987  Con vs. *Alis* + Mel + Trp,  p = 0.0952  *Alis* vs*. Alis* + Mel,  p < 0.0001  *Alis* vs. *Alis* + Trp,  p < 0.0001  *Alis* vs. *Alis* + Mel + Trp,  p < 0.0001  *Alis* + Mel vs. *Alis* + Trp,  p = 0.0192  *Alis* + Mel vs. *Alis* + Mel + Trp,  p = 0.0177  *Alis* + Trp vs. *Alis* + Mel + Trp,  p > 0.9999 | Mean ± s.e.m.  Con  80.05 ± 2.06  *Alis*  44.12 ± 2.60  *Alis* + Mel  82.18 ± 1.69  *Alis* + Trp  70.88 ± 3.28  *Alis* + Mel + Trp  71.08 ± 2.29 |
| **7D**: Behavioral test of TST  Con  (n = 17)  *Alis*  (n = 14)  *Alis* + Mel  (n = 16)  *Alis* + Trp  (n = 17)  *Alis* + Mel + Trp  (n = 19) | One-way ANOVA  Treatment:  F (4, 78) = 9.348,  p < 0.0001 | | Bonferroni’s multiple comparisons test  Con vs. *Alis*,  p < 0.0001  Con vs. *Alis* + Mel,  p > 0.9999  Con vs. *Alis* + Trp,  p = 0.6054  Con vs. *Alis* + Mel + Trp,  p = 0.5214  *Alis* vs. *Alis* + Mel,  p = 0.0004  *Alis* vs. *Alis* + Trp,  p = 0.0010  *Alis* vs. *Alis* + Mel + Trp,  p = 0.0007  *Alis* + Mel vs. *Alis* + Trp,  p > 0.9999  *Alis* + Mel vs. *Alis* + Mel + Trp,  p > 0.9999  *Alis* + Trp vs. *Alis* + Mel + Trp,  p > 0.9999 | Mean ± s.e.m.  Con  107.3 ± 12.6  *Alis*  204.0 ±11.0  *Alis* + Mel  131.2 ± 9.6  *Alis* + Trp  136.9 ± 11.6  *Alis* + Mel + Trp  137.2 ± 10.4 |
| **7E**: Behavioral test of FST  Con  (n = 17)  *Alis*  (n = 14)  *Alis* + Mel  (n = 16)  *Alis* + Trp  (n = 17)  *Alis* + Mel + Trp  (n = 19) | One-way ANOVA  Treatment:  F (4, 78) = 9.404,  p < 0.0001 | | Bonferroni’s multiple comparisons test  Con vs. *Alis*,  p < 0.0001  Con vs. *Alis* + Mel,  p > 0.9999  Con vs. *Alis* + Trp,  p > 0.9999  Con vs. *Alis* + Mel + Trp,  p > 0.9999  *Alis* vs. *Alis +* Mel,  p = 0.0017  *Alis* vs. *Alis +* Trp,  p < 0.0001  *Alis* vs. *Alis* + Mel + Trp,  p < 0*.*0001  *Alis* + Mel vs. *Alis* + Trp,  p > 0.9999  *Alis* + Mel vs. *Alis* + Mel + Trp,  p > 0.9999  *Alis* + Trp vs. *Alis* + Mel + Trp,  p > 0.9999 | Mean ± s.e.m.  Con  89.2 ± 8.1  *Alis*  144.8 ± 8.1  *Alis* + Mel  100.9 ± 6.9  *Alis* + Trp  85.9 ± 9.0  *Alis* + Mel + Trp  91.4 ± 5.1 |
| **7F**: Behavioral test of OFT  Con  (n = 17)  *Alis*  (n = 16)  *Alis* + Mel  (n = 16)  *Alis* + Trp  (n = 17)  *Alis* + Mel + Trp  (n = 19) | One-way ANOVA  Treatment:  F (4, 80) = 0.7605,  p = 0.5540 | | Bonferroni’s multiple comparisons test  Con vs. *Alis*,  p > 0.9999  Con vs. *Alis* + Mel,  p > 0.9999  Con vs. *Alis* + Trp,  p > 0.9999  Con vs. *Alis* + Mel + Trp,  p > 0.9999  *Alis* vs. *Alis* + Mel,  p > 0.9999  *Alis* vs. *Alis* + Trp,  p > 0.9999  *Alis* vs. *Alis* + Mel + Trp,  p > 0.9999  *Alis* + Mel vs. *Alis* + Trp,  p > 0.9999  *Alis* + Mel vs. *Alis* + Mel + Trp,  p > 0.9999  *Alis* + Trp vs. *Alis* + Mel + Trp,  p > 0.9999 | Mean ± s.e.m.  Con  43.947 ± 2.659  *Alis*  44.033 ± 2.669  *Alis* + Mel  43.809 ± 2.674  *Alis* + Trp  46.044 ± 2.223  *Alis +* Mel + Trp  49.017 ± 2.866 |
| **7G**: Behavioral test of OFT  Con  (n = 17)  *Alis*  (n = 16)  *Alis* + Mel  (n = 16)  *Alis* + Trp  (n = 17)  *Alis* + Mel + Trp  (n = 19) | One-way ANOVA  Treatment:  F (4, 80) = 0.5880,  p = 0.6723 | | Bonferroni’s multiple comparisons test  Con vs. *Alis,*  p > 0.9999  Con vs. *Alis* + Mel,  p > 0.9999  Con vs. *Alis* + Trp,  p > 0.9999  Con vs. *Alis* + Mel + Trp,  p > 0.9999  *Alis* vs. *Alis* + Mel,  p > 0.9999  *Alis* vs. *Alis* + Trp,  p > 0.9999  *Alis* vs. *Alis* + Mel + Trp,  p > 0.9999  *Alis* + Mel vs. *Alis* + Trp,  p > 0.9999  *Alis* + Mel vs. *Alis* + Mel + Trp,  p > 0.9999  *Alis* + Trp vs. *Alis* + Mel + Trp,  p > 0.9999 | Mean ± s.e.m.  Con  4.508 ± 0.328  *Alis*  4.810 ± 0.534  *Alis* + Mel  5.124 ± 0.466  *Alis* + Trp  4.503 ± 0.310  *Alis* + Mel + Trp  5.107 ± 0.349 |
| **7H**: Serum estradiol level  Con  (n = 13)  *Alis*  (n = 11)  *Alis* + Mel  (n = 13)  *Alis* + Trp  (n = 10)  *Alis* + Mel + Trp  (n = 12) | One-way ANOVA  Treatment:  F (4, 54) = 2.829,  p = 0.0333 | | Bonferroni’s multiple comparisons test  Con vs. *Alis*,  p > 0.9999  Con vs. *Alis* + Mel,  p > 0.9999  Con vs. *Alis* + Trp,  p > 0.9999  Con vs. *Alis* + Mel + Trp,  p = 0.5779  *Alis* vs. *Alis* + Mel,  p > 0.9999  *Alis* vs. *Alis* + Trp,  p = 0.6699  *Alis* vs. *Alis* + Mel + Trp,  p = 0.0434  *Alis* + Mel vs. *Alis* + Trp,  p > 0.9999  *Alis* + Mel vs. *Alis* + Mel + Trp,  p = 0.1333  *Alis* + Trp vs. *Alis* + Mel + Trp,  p > 0.9999 | Mean ± s.e.m.  Con  1.0000 ± 0.0127  *Alis*  1.0483 ± 0.0454  *Alis* + Mel  1.0257 ± 0.0186  *Alis* + Trp  0.9638 ± 0.0462  *Alis* + Mel + Trp  0.9198 ± 0.0245 |
| **7I**: Fecal tryptophan level  Con  (n = 6)  *Alis*  (n = 5)  *Alis* + Mel  (n = 5)  *Alis* + Trp  (n = 5)  *Alis* + Mel + Trp  (n = 5) | One-way ANOVA  Treatment:  F (4, 21) = 21.70,  p < 0.0001 | | Bonferroni’s multiple comparisons test  Con vs. *Alis*,  p < 0.0001  Con vs. *Alis* + Mel,  p > 0.9999  Con vs. *Alis* + Trp,  p > 0.9999  Con vs. *Alis* + Mel + Trp,  p > 0.9999  *Alis* vs. *Alis* + Mel,  p < 0.0001  *Alis* vs. *Alis* + Trp,  p = 0.0003  *Alis* vs. *Alis* + Mel + Trp,  p < 0.0001  *Alis* + Mel vs. *Alis* + Trp,  p = 0.4175  *Alis* + Mel vs. *Alis* + Mel + Trp,  p > 0.9999  *Alis* + Trp vs. *Alis* + Mel + Trp,  p = 0.0982 | Mean ± s.e.m.  Con  1.0000 ± 0.0542  *Alis*  0.5875 ± 0.0155  *Alis* + Mel  1.0496 ± 0.0431  *Alis* + Trp  0.9165 ± 0.0472  *Alis* + Mel + Trp  1.0908 ± 0.0339 |
| **7J**: Colon tryptophan level  Con  (n = 6)  *Alis*  (n = 5)  *Alis* + Mel  (n = 7)  *Alis* + Trp  (n = 5)  *Alis* + Mel + Trp  (n = 6) | One-way ANOVA  Treatment:  F (4, 24) = 9.750,  p < 0.0001 | | Bonferroni’s multiple comparisons test  Con vs. *Alis*,  p = 0.0003  Con vs. *Alis* + Mel,  p > 0.9999  Con vs. *Alis* + Trp,  p > 0.9999  Con vs. *Alis* + Mel + Trp,  p > 0.9999  *Alis* vs. *Alis* + Mel,  p = 0.0009  *Alis* vs. *Alis* + Trp,  p = 0.0015  *Alis* vs. *Alis* + Mel + Trp,  p = 0.0001  *Alis* + Mel vs. *Alis* + Trp,  p > 0.9999  *Alis* + Mel vs. *Alis* + Mel + Trp,  p > 0.9999  *Alis* + Trp vs. *Alis* + Mel + Trp,  p > 0.9999 | Mean ± s.e.m.  Con  1.0000 ± 0.1334  *Alis*  0.2716 ± 0.0459  *Alis* + Mel  0.9135 ± 0.1055  *Alis* + Trp  0.9327 ± 0.0943  *Alis* + Mel + Trp  1.0497 ± 0.0505 |
| **7K**: Serum tryptophan level  Con  (n = 6)  *Alis*  (n = 5)  *Alis* + Mel  (n = 7)  *Alis* + Trp  (n = 5)  *Alis* + Mel + Trp  (n = 6) | One-way ANOVA  Treatment:  F (4, 24) = 65.44,  p < 0.0001 | | Bonferroni’s multiple comparisons test  Con vs. *Alis*,  p < 0.0001  Con vs. *Alis* + Mel,  p = 0.0318  Con vs. *Alis* + Trp,  p > 0.9999  Con vs. *Alis* + Mel + Trp,  P= 0.0021  *Alis* vs. *Alis* + Mel,  p < 0.0001  *Alis* vs. *Alis* + Trp,  p < 0.0001  *Alis* vs. *Alis* + Mel + Trp,  p < 0.0001  *Alis* + Mel vs. *Alis* + Trp,  p = 0.0738  *Alis* + Mel vs. *Alis* + Mel + Trp,  p > 0.9999  *Alis* + Trp vs. *Alis* + Mel + Trp,  p = 0.0055 | Mean ± s.e.m.  Con  1.0000 ± 0.0253  *Alis*  0.6130 ± 0.0129  *Alis* + Mel  1.1114 ± 0.0222  *Alis* + Trp  1.0067 ± 0.0149  *Alis* + Mel + Trp  1.1540 ± 0.0373 |
| **7L**: NAc tryptophan level  Con  (n = 6)  *Alis*  (n = 5)  *Alis* + Mel  (n = 7)  *Alis* + Trp  (n = 4)  *Alis* + Mel + Trp  (n = 5) | One-way ANOVA  Treatment:  F (4, 22) = 4.516,  p = 0.0082 | | Bonferroni’s multiple comparisons test  Con vs. *Alis,*  p = 0.0180  Con vs. *Alis* + Mel,  p > 0.9999  Con vs. *Alis* + Trp,  p > 0.9999  Con vs. *Alis* + Mel + Trp,  p > 0.9999  *Alis* vs. *Alis* + Mel,  p = 0.0153  *Alis* vs. *Alis* + Trp,  p > 0.9999  *Alis* vs. *Alis* + Mel + Trp,  p = 0.0469  *Alis* + Mel vs. *Alis* + Trp,  p > 0.9999  *Alis* + Mel vs. *Alis* + Mel + Trp,  p > 0.9999  *Alis* + Trp vs. *Alis* + Mel + Trp,  p > 0.9999 | Mean ± s.e.m.  Con  1.0000 ± 0.1022   \|  \| \| --- \|   *Alis*  0.4957 ± 0.0425  *Alis* + Mel  0.9926 ± 0.1036  *Alis* + Trp  0.7621 ± 0.0816  *Alis* + Mel + Trp  0.9626 ± 0.1286 |
| **7M**: Serum 5-HT  Level  Con  (n = 7)  *Alis*  (n = 6)  *Alis* + Mel  (n = 6)  *Alis* + Trp  (n = 5)  *Alis* + Mel + Trp  (n = 6) | One-way ANOVA  Treatment:  F (4, 25) = 87.50,  p < 0.0001 | | Bonferroni’s multiple comparisons test  Con vs. *Alis*,  p < 0.0001  Con vs. *Alis* + Mel,  p > 0.9999  Con vs. *Alis* + Trp,  p > 0.9999  Con vs. *Alis* + Mel + Trp,  p = 0.1193  *Alis* vs. *Alis* + Mel,  p < 0.0001  *Alis* vs. *Alis* + Trp,  p < 0.0001  *Alis* vs. *Alis* + Mel + Trp,  p < 0.0001  *Alis* + Mel vs. *Alis* + Trp,  p > 0.9999  *Alis* + Mel vs. *Alis* + Mel + Trp,  p = 0.0135  *Alis* + Trp vs. *Alis* + Mel + Trp,  p = 0.2724 | Mean ± s.e.m.  Con  1.0000 ± 0.0104   \|  \| \| --- \|   *Alis*  0.7640 ± 0.0172  *Alis* + Mel  1.0157 ± 0.0069  *Alis* + Trp  0.9976 ± 0.0054  *Alis* + Mel + Trp  0.9587 ± 0.0105 |
| **7N**: NAc 5-HT level  Con  (n = 6)  *Alis*  (n = 5)  *Alis* + Mel  (n = 7)  *Alis* + Trp  (n = 5)  *Alis* + Mel + Trp  (n = 6) | One-way ANOVA  Treatment:  F (4, 24) = 4.458,  p = 0.0078 | | Bonferroni’s multiple comparisons test  Con vs. *Alis*,  p = 0.0425  Con vs. *Alis* + Mel,  p > 0.9999  Con vs. *Alis* + Trp,  p > 0.9999  Con vs. *Alis* + Mel + Trp,  p > 0.9999  *Alis* vs. *Alis* + Mel,  p = 0.0117  *Alis* vs. *Alis* + Trp,  p = 0.0242  *Alis* vs. *Alis* + Mel + Trp,  p = 0.0260  *Alis* + Mel vs. *Alis* + Trp,  p > 0.9999  *Alis* + Mel vs. *Alis* + Mel + Trp,  p > 0.9999  *Alis* + Trp vs. *Alis* + Mel + Trp,  p > 0.9999 | Mean ± s.e.m.  Con  1.0000 ± 0.0858  *Alis*  0.4887 ± 0.0504  *Alis* + Mel  1.0651 ± 0.1124  *Alis* + Trp  1.0618 ± 0.1859  *Alis* + Mel + Trp  1.0325 ± 0.0860 |
| **S1B**: Behavioral test of OFT  Sham  (n = 11)  OVX  (n = 11)  OVX + Mel (0.05 mg/mL)  (n = 12)  OVX + Mel (0.1 mg/mL)  (n = 12)  OVX + Mel (0.2 mg/mL)  (n = 11)  OVX + Mel (0.4 mg/mL)  (n = 13) | One-way ANOVA  Treatment:  F (5, 64) = 6.049,  p = 0.0001 | | Bonferroni’s multiple comparisons test  Sham vs. OVX,  p = 0.9311  Sham vs. OVX + Mel (0.05 mg/mL),  p > 0.9999  Sham vs. OVX + Mel (0.1 mg/mL),  p > 0.9999  Sham vs. OVX + Mel (0.2 mg/mL),  p > 0.9999  Sham vs. OVX + Mel (0.4 mg/mL),  p = 0.0017  OVX vs. OVX + Mel (0.05 mg/mL),  p > 0.9999  OVX vs. OVX + Mel (0.1 mg/mL),  p > 0.9999  OVX vs. OVX + Mel (0.2 mg/mL),  p = 0.5907  OVX vs. OVX + Mel (0.4 mg/mL),  p = 0.5568  OVX + Mel (0.05 mg/mL) vs. OVX + Mel (0.1 mg/mL),  p > 0.9999  OVX + Mel (0.05 mg/mL) vs. OVX + Mel (0.2 mg/mL),  p > 0.9999  OVX + Mel (0.05 mg/mL) vs. OVX + Mel (0.4 mg/mL),  p = 0.0022  OVX + Mel (0.1 mg/mL) vs. OVX + Mel (0.2 mg/mL),  p > 0.9999  OVX + Mel (0.1 mg/mL) vs. OVX + Mel (0.4 mg/mL),  p = 0.0019  OVX + Mel (0.2 mg/mL) vs. OVX + Mel (0.4 mg/mL),  p = 0.0008 | Mean ± s.e.m.  Sham  55.277 ± 2.368  OVX  47.225 ± 3.023  OVX + Mel (0.05 mg/mL)  54.630 ± 3.031  OVX + Mel (0.1 mg/mL)  54.777 ± 3.097  OVX + Mel (0.2 mg/mL)  56.144 ± 3.752  OVX + Mel (0.4 mg/mL)  38.552 ± 2.088 |
| **S1C**: Behavioral test of SPT  Sham  (n = 8)  OVX  (n = 8)  OVX + Mel (0.05 mg/mL)  (n = 9)  OVX + Mel (0.1 mg/mL)  (n = 9)  OVX + Mel (0.2 mg/mL)  (n = 8) | One-way ANOVA  Treatment:  F (4, 37) = 23.60,  p < 0.0001 | | Bonferroni’s multiple comparisons test  Sham vs. OVX,  p < 0.0001  Sham vs. OVX + Mel (0.05 mg/mL),  p < 0.0001  Sham vs. OVX + Mel (0.1 mg/mL),  p < 0.0001  Sham vs. OVX + Mel (0.2 mg/mL),  p > 0.9999  OVX vs. OVX + Mel (0.05 mg/mL),  p > 0.9999  OVX vs. OVX + Mel (0.1 mg/mL),  p > 0.9999  OVX vs. OVX + Mel (0.2 mg/mL),  p < 0.0001  OVX + Mel (0.05 mg/mL) vs. OVX + Mel (0.1 mg/mL),  p > 0.9999  OVX + Mel (0.05 mg/mL) vs. OVX + Mel (0.2 mg/mL),  p < 0.0001  OVX + Mel (0.1 mg/mL) vs. OVX + Mel (0.2 mg/mL),  p < 0.0001 | Mean ± s.e.m.  Sham  80.93 ± 2.99  OVX  42.13 ± 3.10  OVX + Mel (0.05 mg/mL)  42.41 ± 6.42  OVX + Mel (0.1 mg/mL)  41.80 ± 4.06  OVX + Mel (0.2 mg/mL)  83.22 ± 4.25 |
| **S1D**: Behavioral test of TST  Sham  (n = 8)  OVX  (n = 8)  OVX + Mel (0.05 mg/mL)  (n = 9)  OVX + Mel (0.1 mg/mL)  (n = 7)  OVX + Mel (0.2 mg/mL)  (n = 8) | One-way ANOVA  Treatment:  F (4, 35) = 6.342,  p = 0.0006 | | Bonferroni’s multiple comparisons test  Sham vs. OVX,  p = 0.0397  Sham vs. OVX + Mel (0.05 mg/mL),  p = 0.9584  Sham vs. OVX + Mel (0.1 mg/mL),  p = 0.1113  Sham vs. OVX + Mel (0.2 mg/mL),  p > 0.9999  OVX vs. OVX + Mel (0.05 mg/mL),  p > 0.9999  OVX vs. OVX + Mel (0.1 mg/mL),  p > 0.9999  OVX vs. OVX + Mel (0.2 mg/mL),  p = 0.0018  OVX + Mel (0.05 mg/mL) vs. OVX + Mel (0.1 mg/mL),  p > 0.9999  OVX + Mel (0.05 mg/mL) vs. OVX + Mel (0.2 mg/mL),  p = 0.0737  OVX + Mel (0.1 mg/mL) vs. OVX + Mel (0.2 mg/mL),  p = 0.0065 | Mean ± s.e.m.  Sham  144.6 ± 15.8  OVX  205.5 ± 10.6  OVX + Mel (0.05 mg/mL)  177.4 ± 16.2  OVX + Mel (0.1 mg/mL)  199.3 ± 10.6  OVX + Mel (0.2 mg/mL)  122.8 ± 13.7   \|  \| \| --- \| \|  \| |
| **S1E**: OFT  Sham  (n = 8)  OVX  (n = 8)  OVX + Mel (0.05 mg/mL)  (n = 9)  OVX + Mel (0.1 mg/mL)  (n = 9)  OVX + Mel (0.2 mg/mL)  (n = 8) | One-way ANOVA  Treatment:  F (4, 37) = 1.181,  p = 0.3352 | | Bonferroni’s multiple comparisons test  Sham vs. OVX,  p > 0.9999  Sham vs. OVX + Mel (0.05 mg/mL),  p > 0.9999  Sham vs. OVX + Mel (0.1 mg/mL),  p > 0.9999  Sham vs. OVX + Mel (0.2 mg/mL),  p = 0.9193  OVX vs. OVX + Mel (0.05 mg/mL),  p > 0.9999  OVX vs. OVX + Mel (0.1 mg/mL),  p > 0.9999  OVX vs. OVX + Mel (0.2 mg/mL),  p > 0.9999  OVX + Mel (0.05 mg/mL) vs. OVX + Mel (0.1 mg/mL),  p > 0.9999  OVX + Mel (0.05 mg/mL) vs. OVX + Mel (0.2 mg/mL),  p > 0.9999  OVX + Mel (0.1 mg/mL) vs. OVX + Mel (0.2 mg/mL),  p > 0.9999 | Mean ± s.e.m.  Sham  7.247 ± 0.532  OVX  5.800 ± 0.699  OVX + Mel (0.05 mg/mL)  6.441 ± 0.768  OVX + Mel (0.1 mg/mL)  7.024 ± 0.770  OVX + Mel (0.2 mg/mL)  5.589 ± 0.366   \|  \| \| --- \| \|  \| |
| **S2B**: Colon claudin-1 mRNA expression  Sham  (n = 5)  OVX  (n = 4)  OVX + Mel  (n = 5) | One-way ANOVA  Treatment:  F (2, 11) = 0.123,  p = 0.8859 | | Bonferroni’s multiple comparisons test  Sham vs. OVX,  p > 0.9999  Sham vs. OVX + Mel,  p > 0.9999  OVX vs. OVX + Mel,  p > 0.9999 | Mean ± s.e.m.  Sham  1.0000 ± 0.0519  OVX  1.0121 ± 0.0824  OVX + Mel  0.9724 ± 0.0427   \|  \| \| --- \| \|  \| |
| **S2C**: Colon occluding mRNA expression  Sham  (n = 5)  OVX  (n = 4)  OVX + Mel  (n = 5) | One-way ANOVA  Treatment:  F (2, 11) = 0.1876,  p = 0.8315 | | Bonferroni’s multiple comparisons test  Sham vs. OVX,  p > 0.9999  Sham vs. OVX + Mel,  p > 0.9999  OVX vs. OVX + Mel,  p > 0.9999 | Mean ± s.e.m.  Sham  1.0000 ± 0.0928  OVX  1.0933 ± 0.1779  OVX + Mel  1.0640 ± 0.0561   \|  \| \| --- \| \|  \| |
| **S2D**: Colon ZO-1  mRNA expression  Sham  (n = 8)  OVX  (n = 8)  OVX + Mel  (n = 8) | One-way ANOVA  Treatment:  F (2, 21) = 2.017,  p = 0.1580 | | Bonferroni’s multiple comparisons test  Sham vs. OVX,  p = 0.6400  Sham vs. OVX + Mel,  p > 0.9999  OVX vs. OVX + Mel,  p = 0.1829 | Mean ± s.e.m.  Sham  1.0000 ± 0.0414  OVX  1.0992 ± 0.0736  OVX + Mel  0.9462 ± 0.0429   \|  \| \| --- \|  \|  \| \| --- \| \|  \| |
| **S2E**: Colon IL-1β  mRNA expression  Sham  (n = 10)  OVX  (n = 10)  OVX + Mel  (n = 9) | One-way ANOVA  Treatment:  F (2, 26) = 1.022,  p = 0.3740 | | Bonferroni’s multiple comparisons test  Sham vs. OVX,  p = 0.6277  Sham vs. OVX + Mel,  p > 0.9999  OVX vs. OVX + Mel,  p = 0.7589 | Mean ± s.e.m.  Sham  1.0000 ± 0.1070  OVX  0.8100 ± 0.0651  OVX + Mel  0.9872 ± 0.1404   \|  \| \| --- \|  \|  \| \| --- \| \|  \| |
| **S2F**: Colon IL-6  mRNA expression  Sham  (n = 10)  OVX  (n = 10)  OVX + Mel  (n = 9) | One-way ANOVA  Treatment:  F (2, 26) = 1.554,  P = 0.2304 | | Bonferroni’s multiple comparisons test  Sham vs. OVX,  p > 0.9999  Sham vs. OVX + Mel,  p = 0.5295  OVX vs. OVX + Mel,  p = 0.3304 | Mean ± s.e.m.  Sham  1.0000 ± 0.0681  OVX  1.0299 ± 0.0949  OVX + Mel  0.8430 ± 0.0692   \|  \| \| --- \|  \|  \| \| --- \| \|  \| |
| **S2G**: Colon TNF-α mRNA expression  Sham  (n = 10)  OVX  (n = 10)  OVX + Mel  (n = 9) | One-way ANOVA  Treatment:  F (2, 26) = 2.830,  p = 0.077 | | Bonferroni’s multiple comparisons test  Sham vs. OVX,  p = 0.1164  Sham vs. OVX + Mel,  p = 0.2063  OVX vs. OVX + Mel,  p > 0.9999 | Mean ± s.e.m.  Sham  1.0000 ± 0.0719  OVX  1.2359 ± 0.1002  OVX + Mel  1.2115 ± 0.0470   \|  \| \| --- \|  \|  \| \| --- \| \|  \| |
| **S3A**: mPFC tryptophan level  Sham  (n = 13)  Sham + Mel  (n = 11)  OVX  (n = 15)  OVX + Mel  (n = 12) | Two-way ANOVA  Interaction:  F (1, 47) = 0.07624,  p = 0.7837  Drug:  F (1, 47) = 0.7089,  p = 0.4041  OVX:  F (1, 47) = 5.851,  p = 0.0195 | | Bonferroni’s multiple comparisons test  Vehicle : Sham vs. Vehicle : OVX,  p = 0.3046  Vehicle : Sham vs. Melatonin : Sham,  p > 0.9999  Vehicle : Sham vs. Melatonin : OVX,  p > 0.9999  Vehicle : OVX vs. Melatonin : Sham,  p = 0.1500  Vehicle : OVX vs. Melatonin : OVX,  p > 0.9999  Melatonin : Sham vs. Melatonin : OVX,  p = 0.9272 | Mean ± s.e.m.  Sham  1.0000 ± 0.0601  Sham + Mel  1.0491 ± 0.1207  OVX  0.7662 ± 0.0736  OVX + Mel  0.8632 ± 0.0933 |
| **S3B**: mPFC 5-HT level  Sham  (n = 13)  Sham + Mel  (n = 11)  OVX  (n = 15)  OVX + Mel  (n = 12) | Two-way ANOVA  Interaction:  F (1, 47) = 0.5135,  p = 0.4772  Drug:  F (1, 47) = 1.880,  p = 0.1768  OVX:  F (1, 47) = 1.143,  p = 0.2906 | | Bonferroni’s multiple comparisons test  Vehicle : Sham vs. Vehicle : OVX,  p > 0.9999  Vehicle : Sham vs. Melatonin : Sham,  p > 0.9999  Vehicle : Sham vs. Melatonin : OVX,  p > 0.9999  Vehicle : OVX vs. Melatonin : Sham,  p = 0.5381  Vehicle : OVX vs. Melatonin : OVX,  p = 0.8118  Melatonin : Sham vs. Melatonin : OVX,  p > 0.9999 | Mean ± s.e.m.  Sham  1.0000 ± 0.0587  Sham + Mel  1.0663 ± 0.1245  OVX  0.8190 ± 0.0913  OVX + Mel  1.0307 ± 0.1256 |
| **S3C**: HIP tryptophan level  Sham  (n = 12)  Sham + Mel  (n = 10)  OVX  (n = 15)  OVX + Mel  (n = 12) | Two-way ANOVA  Interaction:  F (1, 45) = 0.00274,  p = 0.9585  Drug:  F (1, 45) = 0.03528,  p = 0.8518  OVX:  F (1, 45) = 9.552,  p = 0.0034 | | Bonferroni’s multiple comparisons test  Vehicle : Sham vs. Vehicle : OVX,  p = 0.1704  Vehicle : Sham vs. Melatonin : Sham,  p > 0.9999  Vehicle : Sham vs. Melatonin : OVX,  p = 0.1502  Vehicle : OVX vs. Melatonin : Sham,  p = 0.2757  Vehicle : OVX vs. Melatonin : OVX,  p > 0.9999  Melatonin : Sham vs. Melatonin : OVX,  p = 0.2378 | Mean ± s.e.m.  Sham  1.0000 ± 0.0592  Sham + Mel  0.9914 ± 0.0731  OVX  0.8076 ± 0.0543  OVX + Mel  0.7924 ± 0.0676 |
| **S3D**: HIP 5-HT level  Sham  (n = 13)  Sham + Mel  (n = 10)  OVX  (n = 15)  OVX + Mel  (n = 12) | Two-way ANOVA  Interaction:  F (1, 46) = 1.950,  p = 0.1693  Drug:  F (1, 46) = 0.02448,  p = 0.8764  OVX:  F (1, 46) = 3.556,  p = 0.0656 | | Bonferroni’s multiple comparisons test  Vehicle : Sham vs. Vehicle : OVX,  p > 0.9999  Vehicle : Sham vs. Melatonin : Sham,  p > 0.9999  Vehicle : Sham vs. Melatonin : OVX,  p = 0.9090  Vehicle : OVX vs. Melatonin : Sham,  p > 0.9999  Vehicle : OVX vs. Melatonin : OVX,  p > 0.9999  Melatonin : Sham vs. Melatonin : OVX,  p = 0.2011 | Mean ± s.e.m.  Sham  1.0000 ± 0.0826  Sham + Mel  1.0995 ± 0.0826  OVX  0.9607 ± 0.0880  OVX + Mel  0.8361 ± 0.0492 |
| **S4A**: Serum kynurenine level  Sham  (n = 9)  Sham + Mel  (n = 9)  OVX  (n = 10)  OVX + Mel  (n = 10) | Two-way ANOVA  Interaction:  F (1, 34) = 1.394,  p = 0.2460  Drug:  F (1, 34) = 0.1075,  p = 0.7450  OVX:  F (1, 34) = 4.311,  p = 0.0455 | | Bonferroni’s multiple comparisons test  Vehicle : Sham vs. Vehicle : OVX,  p = 0.1652  Vehicle : Sham vs. Melatonin : Sham,  p > 0.9999  Vehicle : Sham vs. Melatonin : OVX,  p > 0.9999  Vehicle : OVX vs. Melatonin : Sham,  p = 0.5894  Vehicle : OVX vs. Melatonin : OVX,  p > 0.9999  Melatonin : Sham vs. Melatonin : OVX,  p > 0.9999 | Mean ± s.e.m.  Sham  1.0000 ± 0.0353  Sham + Mel  0.9641 ± 0.0476  OVX  0.8629 ± 0.0468  OVX + Mel  0.9264 ± 0.0365 |
| **S4B**: mPFC kynurenine level  Sham  (n = 13)  Sham + Mel  (n = 9)  OVX  (n = 15)  OVX + Mel  (n = 12) | Two-way ANOVA  Interaction:  F (1, 45) = 2.466,  p = 0.1233  Drug:  F (1, 45) = 0.5838,  p = 0.4488  OVX:  F (1, 45) = 3.045,  p = 0.0878 | | Bonferroni’s multiple comparisons test  Vehicle : Sham vs. Vehicle : OVX,  p = 0.0869  Vehicle : Sham vs. Melatonin : Sham,  p > 0.9999  Vehicle : Sham vs. Melatonin : OVX,  p > 0.9999  Vehicle : OVX vs. Melatonin : Sham,  p = 0.5429  Vehicle : OVX vs. Melatonin : OVX,  p = 0.5192  Melatonin : Sham vs. Melatonin : OVX,  p > 0.9999 | Mean ± s.e.m.  Sham  1.0000 ± 0.0606  Sham + Mel  0.9369 ± 0.0841  OVX  0.7404 ± 0.0739  OVX + Mel  0.9232 ± 0.0885 |
| **S4C**: HIP kynurenine level  Sham  (n = 12)  Sham + Mel  (n = 9)  OVX  (n = 15)  OVX + Mel  (n = 12) | Two-way ANOVA  Interaction:  F (1, 44) = 0.7166,  p = 0.4019  Drug:  F (1, 44) = 0.3784,  p = 0.5416  OVX:  F (1, 44) = 0.5224,  p = 0.4736 | | Bonferroni’s multiple comparisons test  Vehicle : Sham vs. Vehicle : OVX,  p > 0.9999  Vehicle : Sham vs. Melatonin : Sham,  p > 0.9999  Vehicle : Sham vs. Melatonin : OVX,  p > 0.9999  Vehicle : OVX vs. Melatonin : Sham,  p > 0.9999  Vehicle : OVX vs. Melatonin : OVX,  p > 0.9999  Melatonin : Sham vs. Melatonin : OVX,  p > 0.9999 | Mean ± s.e.m.  Sham  1.0000 ± 0.0364  Sham + Mel  0.8969 ± 0.0748  OVX  0.8893 ± 0.0684  OVX + Mel  0.9056 ± 0.0862 |
| **S4D**: NAc kynurenine level  Sham  (n = 9)  Sham + Mel  (n = 11)  OVX  (n = 10)  OVX + Mel  (n = 10) | Two-way ANOVA  Interaction:  F (1, 36) = 0.07883,  p = 0.7805  Drug:  F (1, 36) = 0.7907,  p = 0.3798  OVX:  F (1, 36) = 0.01500,  p = 0.9032 | | Bonferroni’s multiple comparisons test  Vehicle : Sham vs. Vehicle : OVX,  p > 0.9999  Vehicle : Sham vs. Melatonin : Sham,  p > 0.9999  Vehicle : Sham vs. Melatonin : OVX,  p > 0.9999  Vehicle : OVX vs. Melatonin : Sham,  p > 0.9999  Vehicle : OVX vs. Melatonin : OVX,  p > 0.9999  Melatonin : Sham vs. Melatonin : OVX,  p > 0.9999 | Mean ± s.e.m.  Sham  1.0000 ± 0.0610  Sham + Mel  09622 ± 0.0624  OVX  1.0098 ± 0.0715  OVX + Mel  0.9372 ± 0.0496 |
| **S5A**: mPFC PSD95 expression  Sham  (n = 6)  Sham + Mel  (n = 6)  OVX  (n = 6)  OVX + Mel  (n = 6) | Two-way ANOVA  Interaction:  F (1, 20) = 0.7309,  p = 0.4027  Drug:  F (1, 20) = 2.184,  p = 0.1551  OVX:  F (1, 20) = 1.175,  p = 0.2912 | | Bonferroni’s multiple comparisons test  Vehicle : Sham vs. Vehicle : OVX,  p > 0.9999  Vehicle : Sham vs. Melatonin : Sham,  p > 0.9999  Vehicle : Sham vs. Melatonin : OVX,  p = 0.5106  Vehicle : OVX vs. Melatonin : Sham,  p > 0.9999  Vehicle : OVX vs. Melatonin : OVX,  p = 0.6881  Melatonin : Sham vs. Melatonin : OVX,  p > 0.9999 | Mean ± s.e.m.  Sham  1.0000 ± 0.1068  Sham + Mel  1.1041 ± 0.1918  OVX  1.0383 ± 0.1304  OVX + Mel  1.4284 ± 0.2160 |
| **S5A**: mPFC BDNF expression  Sham  (n = 6)  Sham + Mel  (n = 6)  OVX  (n = 6)  OVX + Mel  (n = 6) | Two-way ANOVA  Interaction:  F (1, 20) = 0.03528,  p = 0.8529  Drug:  F (1, 20) = 0.05425,  p = 0.8182  OVX:  F (1, 20) = 7.083,  p = 0.0150 | | Bonferroni’s multiple comparisons test  Vehicle : Sham vs. Vehicle : OVX,  p = 0.5736  Vehicle : Sham vs. Melatonin : Sham,  p > 0.9999  Vehicle : Sham vs. Melatonin : OVX,  p = 0.6083  Vehicle : OVX vs. Melatonin : Sham,  p = 0.3244  Vehicle : OVX vs. Melatonin : OVX,  p > 0.9999  Melatonin : Sham vs. Melatonin : OVX,  p = 0.3454 | Mean ± s.e.m.  Sham  1.0000 ± 0.0559  Sham + Mel  1.0510 ± 0.2018  OVX  0.7001 ± 0.1064  OVX + Mel  0.7056 ± 0.0600 |
| **S5B**: HIP PSD95 expression  Sham  (n = 6)  Sham + Mel  (n = 6)  OVX  (n = 6)  OVX + Mel  (n = 6) | Two-way ANOVA  Interaction:  F (1, 20) = 1.318,  p = 0.2644  Drug:  F (1, 20) = 5.218,  p = 0.0334  OVX:  F (1, 20) = 0.04286,  p = 0.8381 | | Bonferroni’s multiple comparisons test  Vehicle : Sham vs. Vehicle : OVX,  p > 0.9999  Vehicle : Sham vs. Melatonin : Sham,  p > 0.9999  Vehicle : Sham vs. Melatonin : OVX,  p = 0.5605  Vehicle : OVX vs. Melatonin : Sham,  p =0.9446  Vehicle : OVX vs. Melatonin : OVX,  p = 0.1487  Melatonin : Sham vs. Melatonin : OVX,  p > 0.9999 | Mean ± s.e.m.  Sham  1.0000 ± 0.0689  Sham + Mel  0.9006 ± 0.1339  OVX  1.0824 ± 0.0601  OVX + Mel  0.7820 ± 0.0659 |
| **S5B**: HIP BDNF expression  Sham  (n = 6)  Sham + Mel  (n = 6)  OVX  (n = 6)  OVX + Mel  (n = 6) | Two-way ANOVA  Interaction:  F (1, 20) = 0.2820,  p = 0.6012  Drug:  F (1, 20) = 0.1419,  p = 0.7103  OVX:  F (1, 20) = 0.2434,  p = 0.6271 | | Bonferroni’s multiple comparisons test  Vehicle : Sham vs. Vehicle : OVX,  p > 0.9999  Vehicle : Sham vs. Melatonin : Sham,  p > 0.9999  Vehicle : Sham vs. Melatonin : OVX,  p > 0.9999  Vehicle : OVX vs. Melatonin : Sham,  p > 0.9999  Vehicle : OVX vs. Melatonin : OVX,  p > 0.9999  Melatonin : Sham vs. Melatonin : OVX,  p > 0.9999 | Mean ± s.e.m.  Sham  1.0000 ± 0.1306  Sham + Mel  1.1519 ± 0.1916  OVX  1.0063 ± 0.1676  OVX + Mel  0.9805 ± 0.1734 |
| **S5D**: mPFC total density μm^-1^  Sham  (n = 9)  Sham + Mel  (n = 9)  OVX  (n = 9)  OVX + Mel  (n = 9) | | Two-way ANOVA  Interaction:  F (1, 32) = 0.9650,  p = 0.3333  Drug:  F (1, 32) = 2.190,  p = 0.1486  OVX:  F (1, 32) = 2.989,  p = 0.0935 | Bonferroni’s multiple comparisons test  Vehicle : Sham vs. Vehicle : OVX,  p = 0.3851  Vehicle : Sham vs. Melatonin : Sham,  p > 0.9999  Vehicle : Sham vs. Melatonin : OVX,  p > 0.9999  Vehicle : OVX vs. Melatonin : Sham,  p = 0.1809  Vehicle : OVX vs. Melatonin : OVX,  p = 0.5476  Melatonin : Sham vs. Melatonin : OVX,  p > 0.9999 | Mean ± s.e.m.  Sham  3.23 ± 0.22  Sham + Mel  3.38 ± 0.38  OVX  2.42 ± 0.20  OVX + Mel  3.16 ± 0.35 |
| **S5E**: mPFC stubby density μm^-1^  Sham  (n = 9)  Sham + Mel  (n = 9)  OVX  (n = 9)  OVX + Mel  (n = 9) | | Two-way ANOVA  Interaction:  F (1, 32) = 0.2628,  p = 0.6117  Drug:  F (1, 32) = 3.352,  p = 0.0765  OVX:  F (1, 32) = 1.550,  p = 0.2222 | Bonferroni’s multiple comparisons test  Vehicle : Sham vs. Vehicle : OVX,  p > 0.9999  Vehicle : Sham vs. Melatonin : Sham,  p = 0.6438  Vehicle : Sham vs. Melatonin : OVX,  p = 0.2229  Vehicle : OVX vs. Melatonin : Sham,  p > 0.9999  Vehicle : OVX vs. Melatonin : OVX,  p > 0.9999  Melatonin : Sham vs. Melatonin : OVX,  p > 0.9999 | Mean ± s.e.m.  Sham  0.27 ± 0.03  Sham + Mel  0.40 ± 0.07  OVX  0.37 ± 0.04  OVX + Mel  0.44 ± 0.07 |
| **S5F**: mPFC mushroom density μm^-1^  Sham  (n = 9)  Sham + Mel  (n = 9)  OVX  (n = 9)  OVX + Mel  (n = 9) | | Two-way ANOVA  Interaction:  F (1, 32) = 1.305,  p = 0.2618  Drug:  F (1, 32) = 0.1835,  p = 0.6712  OVX:  F (1, 32) = 0.2117,  p = 0.6486 | Bonferroni’s multiple comparisons test  Vehicle : Sham vs. Vehicle : OVX,  p > 0.9999  Vehicle : Sham vs. Melatonin : Sham,  p > 0.9999  Vehicle : Sham vs. Melatonin : OVX,  p > 0.9999  Vehicle : OVX vs. Melatonin : Sham,  p > 0.9999  Vehicle : OVX vs. Melatonin : OVX,  p > 0.9999  Melatonin : Sham vs. Melatonin : OVX,  p > 0.9999 | Mean ± s.e.m.  Sham  0.52 ± 0.07  Sham + Mel  0.47 ± 0.05  OVX  0.47 ± 0.07  OVX + Mel  0.58 ± 0.09 |
| **S5G**: mPFC thin density μm^-1^  Sham  (n = 9)  Sham + Mel  (n = 9)  OVX  (n = 9)  OVX + Mel  (n = 9) | | Two-way ANOVA  Interaction:  F (1, 32) = 0.9666,  p = 0.3329  Drug:  F (1, 32) = 1.642,  p = 0.2092  OVX:  F (1, 32) = 6.283,  p = 0.0175 | Bonferroni’s multiple comparisons test  Vehicle : Sham vs. Vehicle : OVX,  p = 0.1148  Vehicle : Sham vs. Melatonin : Sham,  p > 0.9999  Vehicle : Sham vs. Melatonin : OVX,  p > 0.9999  Vehicle : OVX vs. Melatonin : Sham,  p = 0.0695  Vehicle : OVX vs. Melatonin : OVX,  p = 0.7147  Melatonin : Sham vs. Melatonin : OVX,  p > 0.9999 | Mean ± s.e.m.  Sham  2.44 ± 0.20  Sham + Mel  2.51 ± 0.35  OVX  1.58 ± 0.15  OVX + Mel  2.14 ± 0.24 |
| **S5I**: HIP total density μm^-1^  Sham  (n = 9)  Sham + Mel  (n = 9)  OVX  (n = 9)  OVX + Mel  (n = 9) | | Two-way ANOVA  Interaction:  F (1, 32) = 0.8884  p = 0.3530  Drug:  F (1, 32) = 0.8375,  p = 0.3670  OVX:  F (1, 32) = 0.3662,  p = 0.5493 | Bonferroni’s multiple comparisons test  Vehicle : Sham vs. Vehicle : OVX,  p > 0.9999  Vehicle : Sham vs. Melatonin : Sham,  p > 0.9999  Vehicle : Sham vs. Melatonin : OVX,  p > 0.9999  Vehicle : OVX vs. Melatonin : Sham,  p > 0.9999  Vehicle : OVX vs. Melatonin : OVX,  p > 0.9999  Melatonin : Sham vs. Melatonin : OVX,  p > 0.9999 | Mean ± s.e.m.  Sham  4.84 ± 0.55  Sham + Mel  3.87 ± 0.49  OVX  4.03 ± 0.43  OVX + Mel  4.04 ± 0.62 |
| **S5J**: HIP stubby density μm^-1^  Sham  (n = 9)  Sham + Mel  (n = 9)  OVX  (n = 9)  OVX + Mel  (n = 9) | | Two-way ANOVA  Interaction:  F (1, 32) = 0.09074,  p = 0.7652  Drug:  F (1, 32) = 0.2360,  p = 0.6304  OVX:  F (1, 32) = 0.03777,  p = 0.8471 | Bonferroni’s multiple comparisons test  Vehicle : Sham vs. Vehicle : OVX,  p > 0.9999  Vehicle : Sham vs. Melatonin : Sham,  p > 0.9999  Vehicle : Sham vs. Melatonin : OVX,  p > 0.9999  Vehicle : OVX vs. Melatonin : Sham,  p > 0.9999  Vehicle : OVX vs. Melatonin : OVX,  p > 0.9999  Melatonin : Sham vs. Melatonin : OVX,  p > 0.9999 | Mean ± s.e.m.  Sham  0.63 ± 0.12  Sham + Mel  0.61 ± 0.11  OVX  0.69 ± 0.09  OVX + Mel  0.60 ± 0.13 |
| **S5K**: HIP mushroom density μm^-1^  Sham  (n = 9)  Sham + Mel  (n = 9)  OVX  (n = 9)  OVX + Mel  (n = 9) | | Two-way ANOVA  Interaction:  F (1, 32) = 0.1212,  p = 0.7300  Drug:  F (1, 32) = 0.3538,  p = 0.5561  OVX:  F (1, 32) = 1.314,  p = 0.2602 | Bonferroni’s multiple comparisons test  Vehicle : Sham vs. Vehicle : OVX,  p > 0.9999  Vehicle : Sham vs. Melatonin : Sham,  p > 0.9999  Vehicle : Sham vs. Melatonin : OVX,  p > 0.9999  Vehicle : OVX vs. Melatonin : Sham,  p > 0.9999  Vehicle : OVX vs. Melatonin : OVX,  p > 0.9999  Melatonin : Sham vs. Melatonin : OVX,  p > 0.9999 | Mean ± s.e.m.  Sham  0.59 ± 0.08  Sham + Mel  0.52 ± 0.09  OVX  0.48 ± 0.05  OVX + Mel  0.46 ± 0.08 |
| **S5L**: HIP thin density μm^-1^  Sham  (n = 9)  Sham + Mel  (n = 9)  OVX  (n = 9)  OVX + Mel  (n = 9) | | Two-way ANOVA  Interaction:  F (1, 32) = 1.108,  p = 0.3004  Drug:  F (1, 32) = 0.6328,  p = 0.4322  OVX:  F (1, 32) = 0.2899,  p = 0.5940 | Bonferroni’s multiple comparisons test  Vehicle : Sham vs. Vehicle : OVX,  p > 0.9999  Vehicle : Sham vs. Melatonin : Sham,  p > 0.9999  Vehicle : Sham vs. Melatonin : OVX,  p > 0.9999  Vehicle : OVX vs. Melatonin : Sham,  p > 0.9999  Vehicle : OVX vs. Melatonin : OVX,  p > 0.9999  Melatonin : Sham vs. Melatonin : OVX,  p > 0.9999 | Mean ± s.e.m.  Sham  3.62 ± 0.44  Sham + Mel  2.74 ± 0.48  OVX  2.86 ± 0.39  OVX + Mel  2.98 ± 0.59 |
| **S6A**: Serum I3C level  Con  (n = 13)  *Alis*  (n = 11)  *Alis* + Mel  (n = 13)  *Alis* + Trp  (n = 10)  *Alis* + Mel + Trp  (n = 12) | | One-way ANOVA  Treatment:  F (4, 54) = 3.908,  p = 0.0074 | Bonferroni’s multiple comparisons test  Con vs. *Alis*,  p = 0.0286  Con vs. *Alis* + Mel,  p > 0.9999  Con vs. *Alis* + Trp,  p = 0.6876  Con vs. *Alis* + Mel + Trp,  p > 0.9999  *Alis* vs. *Alis* + Mel,  p = 0.4830  *Alis* vs. *Alis* + Trp,  p > 0.9999  *Alis* vs. *Alis* + Mel + Trp,  p = 0.0117  *Alis* + Mel vs. *Alis* + Trp,  p > 0.9999  *Alis* + Mel vs. *Alis* + Mel + Trp,  p > 0.9999  *Alis* + Trp vs. *Alis* + Mel + Trp,  p > 0.3399 | Mean ± s.e.m.   \|  \| \| --- \|   Con  1.0000 ± 0.0223  *Alis*  0.8501 ± 0.0373  *Alis* + Mel  0.9470 ± 0.0373  *Alis* + Trp  0.9086 ± 0.0391  *Alis* + Mel + Trp  1.0176 ± 0.0342 |
